# Smart hybrid nanomaterials for chronic infections: microbiome-responsive and sustainable therapeutic platforms

**DOI:** 10.1186/s12951-025-03712-4

**Published:** 2025-11-03

**Authors:** Hina Singh, Sri Renukadevi Balusamy, Johan Sukweenadhi, Muthupandian Saravanan, Mohanprasanth Aruchamy, Ivan Mijakovic, Priyanka Singh

**Affiliations:** 1 Division of Biomedical Sciences, School of Medicine, University of California, Riverside, CA 92521 USA; 2 Department of Food Science and Biotechnology, Sejong University, Gwangjin-Gu, Seoul, 05006 Republic of Korea; 3 Faculty of Biotechnology, University of Surabaya, Raya Kalirungkut, Kalirungkut, Surabaya, 60293 Indonesia; 4 Prince Fahad bin Sultan Chair for Biomedical Research, University of Tabuk, Tabuk, 71491 Saudi Arabia; 5 AMR & Nanotherapeutics Lab, Department of Pharmacology, Saveetha Dental College and Hospitals, Saveetha Institute of Medical and Technical Sciences, Chennai, 600 077 India; 6 The Novo Nordisk Foundation Center for Biosustainability, Technical University of Denmark, Kongens Lyngby, 2800 Denmark; 7 Systems and Synthetic Biology Division, Department of Biology and Biological Engineering, Chalmers University of Technology, Gothenburg, 412 96 Sweden

**Keywords:** Chronic infections, Microbiome therapy, Sustainable nanomaterials, Smart hybrid nanomaterials, Artificial intelligence, Personalized medicine

## Abstract

Chronic wounds remain a persistent clinical challenge due to their complex pathology involving prolonged inflammation, microbial biofilms, and impaired tissue regeneration. Emerging research highlights the dynamic role of the human microbiome in modulating immune responses and influencing wound healing outcomes. Disruptions to this microbial balance, termed dysbiosis, not only exacerbate infection but also hinder therapeutic efficacy. Recent advances in smart and sustainable nanotechnology offer promising avenues to address these challenges. By integrating microbiome-sensitive mechanisms with targeted delivery systems, bio-based nanomaterials can overcome multidrug resistance, modulate inflammation, and accelerate healing. This review explores the molecular interplay between chronic infections and host immunity, highlights next-generation nanomaterials for regenerative applications, and proposes a sustainable paradigm that bridges clinical effectiveness with environmental responsibility.

**Graphical abstract:**

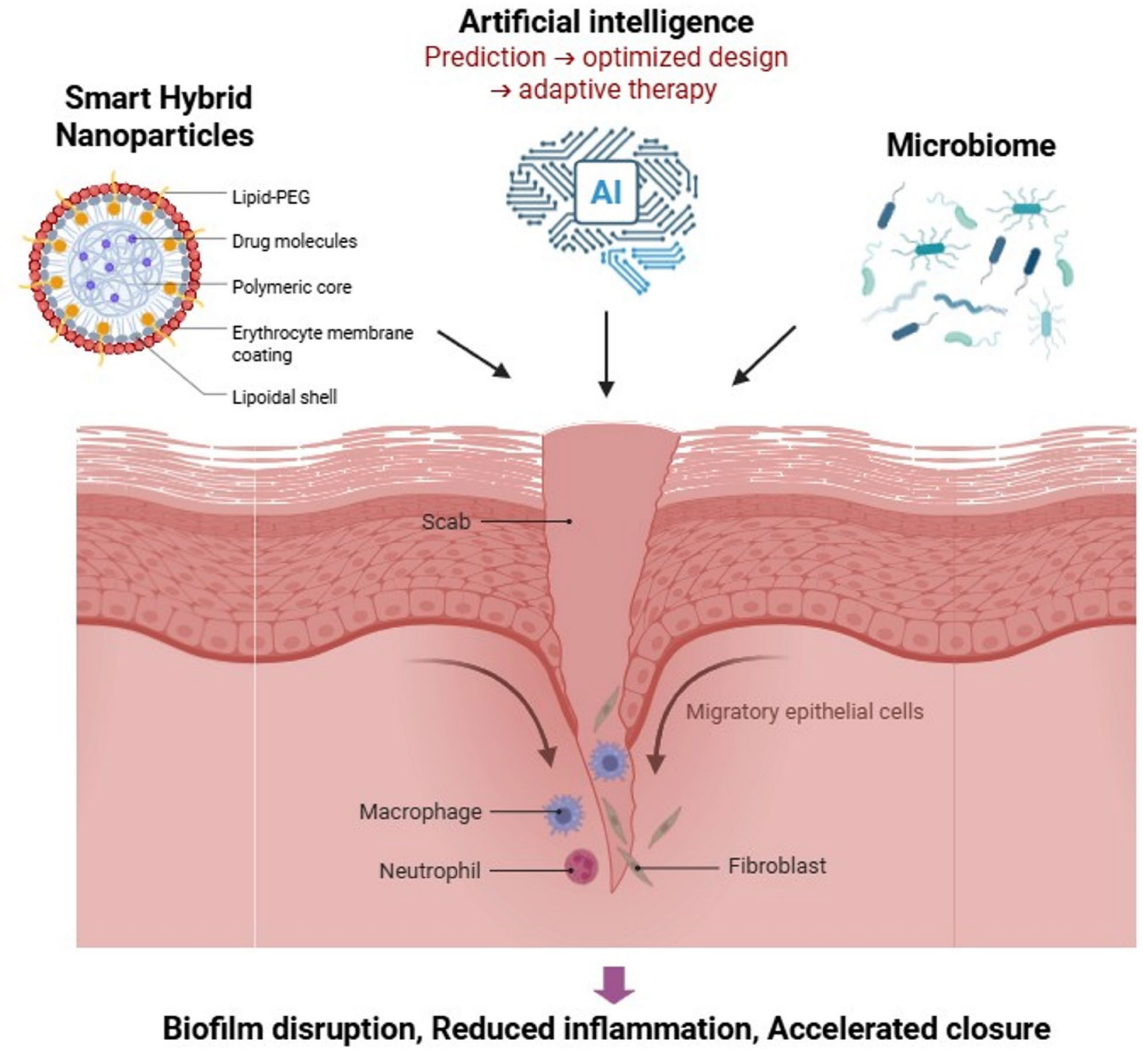

## Introduction

Chronic infections, characterized by persistent inflammation and microbial colonization that fail to resolve within expected timeframes, represent a serious and debilitating Health challenge. For example, approximately 6.3% of people with diabetes worldwide suffer from diabetic foot ulcers (DFUs), and an estimated 8 million people live with chronic wounds that often progress to severe complications such as sepsis or amputation [1]. These infections not only diminish quality of life but also impose a substantial socio-economic burden, with billions of dollars spent annually on treatment and disability support. A major concern is their strong association with antimicrobial resistance (AMR), which has rendered many conventional therapies ineffective and significantly increased mortality rates. AMR, largely driven by the misuse and overuse of antibiotics, has facilitated the rise of multidrug-resistant pathogens that evade both antimicrobial agents and host immune defenses [2]. The World Health Organization (WHO) estimates that AMR contributes to at least 700,000 deaths annually, a number projected to rise to 10 million by 2050 if not urgently addressed [3].

Chronic wounds exemplify this challenge, sharing pathophysiological features with systemic chronic infections, including persistent microbial colonization, immune evasion, and biofilm formation. Unlike acute wounds, which follow a well-defined healing cascade of re-epithelialization, fibroblast proliferation, and angiogenesis, chronic wounds remain trapped in a prolonged inflammatory phase characterized by microbial persistence and dysregulated immune responses [4–6]. This pathological state hinders epithelialization and vascularization, enabling biofilm-forming pathogens such as *Staphylococcus aureus* and *Pseudomonas aeruginosa* to dominate the wound microenvironment [7]. Central to the persistence of chronic infections is the role of the microbiome, a critical regulator of health and disease. Dysbiosis, or microbial imbalance, exacerbates chronic inflammation and disrupts tissue regeneration. In chronic wounds and mucosal infections, a dysbiotic microbial environment supports the growth of virulent, biofilm-producing organisms while suppressing beneficial commensals. Restoring microbial balance has therefore emerged as a promising therapeutic goal. Microbiome-aware therapies including probiotics, prebiotics, and phage-based interventions seek to modulate the host–microbe interface, suppress harmful pathogens, and enhance beneficial microbial communities [8]. These interventions are informed by a deeper understanding of host–pathogen interactions and represent a shift toward therapies that not only eradicate infection but also restore immune homeostasis and epithelial integrity.

Complementing these biologically informed therapies is the development of smart hybrid nanomaterials, engineered to respond to specific biological stimuli such as pH shifts, oxidative stress, and enzymatic activity. These materials enable precise drug delivery and effective disruption of biofilms in infected tissues [9]. By combining antimicrobial and regenerative functions, smart nanomaterials directly address the dual challenges of infection control and impaired healing limitations that conventional therapies often fail to overcome due to incomplete drug release, cytotoxicity, or poor efficacy against biofilm-associated infections.

Sustainability has emerged as a central theme in modern biomedical innovation. While traditional synthetic nanomaterials demonstrate strong therapeutic efficacy, their persistence and toxicity raise significant environmental concerns. In contrast, green nanomaterials derived from renewable resources such as chitosan, cellulose, and marine polysaccharides provide safer, green alternatives. These green platforms combine excellent biocompatibility with antimicrobial efficacy, showing potent activity against resistant pathogens while minimizing ecological harm. Thus, they align therapeutic development with global sustainability goals [10, 11].

Artificial intelligence (AI) has further transformed the design and optimization of advanced therapeutic platforms. By harnessing large datasets and predictive algorithms, AI enables the discovery and fine-tuning of nanomaterials tailored to specific biological environments [12]. Machine learning (ML) models are particularly powerful in predicting nanomaterial–cell interactions, thereby accelerating the development of personalized therapies [13]. Beyond design, AI also supports real-time monitoring of therapeutic efficacy and dynamic adjustment of treatments, ensuring that interventions remain adaptive and effective throughout infection [14, 15]. Together, the convergence of microbiome-centered therapies, smart hybrid nanomaterials, and AI-driven innovations represents a paradigm shift in the management of chronic infections. These interdisciplinary approaches promise clinically relevant, scalable, and environmentally sustainable solutions. In this review, we redefine the scope by focusing specifically on microbiome-sensitive and green smart nanomaterials, highlighting their mechanistic interactions with chronic wound microenvironments. We critically synthesize advances in microbiome-targeted therapies, sustainable nanomaterials, hybrid systems with dual antimicrobial and regenerative functions, and AI-driven design strategies. Finally, we discuss translational gaps including scalability, regulatory hurdles, and patient-specific variability that must be addressed for successful clinical implementation.

## Microbiome and chronic wound infections: mechanisms and interventions

Building upon the clinical challenges outlined in the introduction, this section explores the central role of the microbiome in chronic infections and its disruption in wound healing. Prolonged inflammation and microbial colonization underlie chronic infections such as diabetic ulcers and other non-healing wounds. These conditions are persistent and difficult to treat, largely due to microbial persistence and dysregulated immune responses. One of the most prevalent forms, diabetic ulcers, primarily affects individuals with poorly managed diabetes. These ulcers, typically on the legs and feet, exhibit delayed healing due to compromised blood supply, impaired immune function, and neuropathy. Infections in such wounds significantly hinder recovery and can escalate to severe complications such as sepsis or amputation [16]. Understanding how microbial populations influence chronic conditions requires examining their impact on the wound environment, immune modulation, and infection persistence.

### Acute vs. chronic wound microbiota

Acute and chronic wounds have fundamentally different healing processes, immunological responses, and underlying causes. Cuts, burns, or surgical incisions are examples of acute wounds that usually occur after an abrupt trauma or damage. These wounds typically heal in a predictable, ordered fashion, with tissue healing aided by a well-coordinated immune response. The four overlapping phases of wound healing are hemostasis, inflammation, proliferation, and remodeling. During the acute phase, blood vessels constrict to minimize bleeding, and clot forms. Inflammation follows, with neutrophils and macrophages playing key roles in clearing pathogens and damaged tissue. This is followed by proliferation, where new tissue and blood vessels form, and eventually, tissue remodeling occurs, restoring the wound site to its normal structure and function. In acute wounds, the immune response is generally well-regulated, allowing for a relatively quick resolution of inflammation and the restoration of tissue integrity.

In contrast, chronic wounds are often defined by prolonged inflammation and impaired healing, typically lasting for weeks or months. These wounds fail to progress through the normal healing phases and are characterized by a persistent inflammatory environment. In chronic wounds, there is excessive secretion of inflammatory mediators, such as cytokines and matrix metalloproteinases (MMPs), which degrade growth factors and the extracellular matrix (ECM), further impairing healing. Additionally, macrophages in chronic wounds often fail to switch from a pro-inflammatory phenotype to a pro-healing phenotype, resulting in a feed-forward loop of inflammation that prevents resolution. While acute wounds generally heal through an orchestrated immune response that supports tissue repair, chronic wounds are marked by immune dysregulation and an inability to transition from inflammation to healing [17]. Chronic wounds are more difficult to treat and often require advanced, targeted therapies. The prolonged nature of chronic wound healing also poses challenges in terms of infection control and microbial management, as biofilms and persistent pathogens complicate the healing process. Chronic wounds often harbor polymicrobial communities dominated by pathogens (e.g., *S. aureus*, *P. aeruginosa*, *Enterococcus faecalis*, *E. coli*), reflecting dysbiosis that drives inflammation and delayed healing [17].

### Microbial dysbiosis and biofilm formation in chronic wounds

**Fig. 1:**
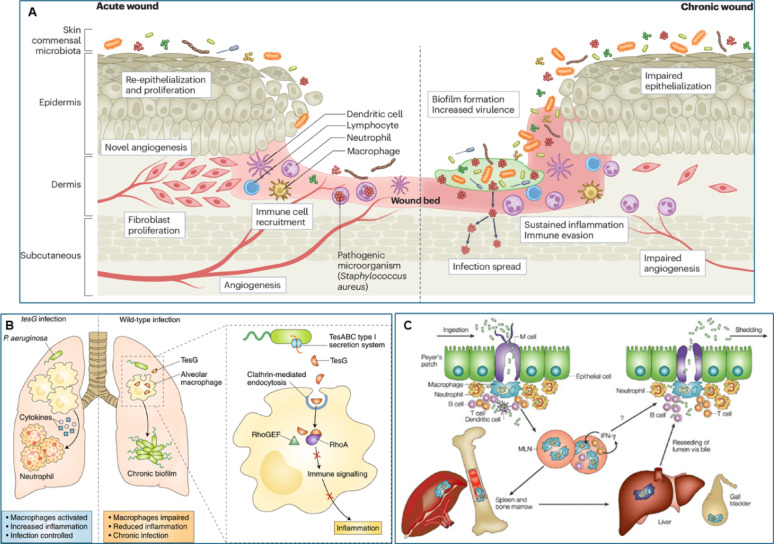
Pathophysiological mechanisms underlying chronic wounds and infections, illustrating immune evasion, biofilm persistence, and shared pathways across wound and systemic infections. (**A**) The bifurcation between acute and chronic wound healing cascades; (**B**) *P. aeruginosa* TesG-mediated inhibition of macrophage RhoA signaling, driving biofilm formation; (**C**) illustrates *Salmonella enterica* invasion and systemic dissemination through Peyer’s patches, establishing chronic reservoirs (This figure adapted from [4–6])

When the microbiome’s equilibrium is disrupted (dysbiosis), opportunistic and pathogenic bacteria such as *P aeruginosa* and *S. aureus* dominate, often forming resilient biofilms. In pulmonary infections, *P. aeruginosa* secretes TesG via the TesABC Type I secretion system, which impairs alveolar macrophage activity by inhibiting RhoA-mediated immune signaling, thereby promoting biofilm formation and infection chronicity (Fig. 1). The pathophysiology of chronic wounds shares striking parallels with systemic chronic infections. Infections with TesG-deficient strains trigger robust macrophage activation, higher cytokine production, and neutrophil recruitment, leading to more effective infection control. A similar pattern is observed in gastrointestinal infections, where pathogens such as *Salmonella enterica* invade Peyer’s patches in the intestinal epithelium, penetrate immune cells like macrophages and dendritic cells, and disseminate to mesenteric lymph nodes, spleen, and bone marrow. These bacteria establish chronic reservoirs that evade immune responses, including interferon-gamma (IFN-γ) signaling, and continuously reseed the intestinal lumen via bile, perpetuating cycles of infection and shedding. Chronic wounds show reduced microbial diversity, sustaining inflammation and increasing susceptibility to infection. This imbalance also alters local immune surveillance and signaling, favoring pathogen persistence and biofilm formation. Furthermore, unbalanced microbiota may worsen systemic diseases such as diabetes, further complicating healing. Developing novel therapeutic approaches targeted at re-establishing microbial balance and facilitating efficient wound healing requires an understanding of the connection between persistent infection and microbiome imbalance [18].

#### Microbial biofilms in wound environments

Rather than serving as mere microbial aggregates, biofilms in chronic wounds function as highly organized communities that confer multidrug resistance, metabolic adaptation, and immune evasion. Within the biofilm matrix, pathogens such as *P. aeruginosa* and *S. aureus* adopt slow-growth phenotypes and alter gene expression, increasing tolerance to both antibiotics and host defenses. This microenvironment protects resident microbes from phagocytic clearance and facilitates horizontal gene transfer, amplifying resistance traits. The persistence of biofilms sustains a low-level but chronic inflammatory state, releasing proteases and toxins that degrade ECM and growth factors, thereby stalling the proliferative phase of wound repair. The recalcitrant nature of biofilm-associated infections highlights the need for next-generation therapeutic interventions, including nanomaterial-based systems capable of disrupting biofilm integrity while simultaneously delivering antimicrobials.

#### Immune dysfunction and hyperglycemia

Diabetic ulcers are a common and serious complication of diabetes, particularly in individuals with poorly controlled blood glucose levels. They primarily develop on the feet and legs due to a combination of factors, including neuropathy (nerve damage), poor circulation, and impaired immune function. These ulcers are often classified as chronic wounds because they are slow to heal and may persist for months or even years without proper treatment. The development of DFU begins with a mechanical injury or pressure on the foot, often due to neuropathy, which impairs the ability to sense pain and prevent tissue damage. In individuals with diabetes, particularly those with poorly controlled blood sugar levels, this injury is more prone to infection. The high glucose environment provides a rich nutrient source for bacteria, enabling their rapid colonization. As a result, the wound becomes an ideal site for microbial invasion. Common pathogens are often introduced to the wound through direct contact or from endogenous sources. Upon injury, the body’s innate immune system attempts to defend against microbial invasion. Immune cells, such as neutrophils and macrophages, are rapidly recruited to the wound site in response to inflammatory signals. These cells attempt to clear pathogens through phagocytosis, the process by which the immune cells engulf and destroy invading microorganisms. Neutrophils are typically the first responders and attempt to neutralize bacteria by releasing ROS and antimicrobial peptides. Macrophages, in addition to clearing debris, release cytokines and growth factors to coordinate further immune responses and promote tissue repair. However, in diabetic individuals, this immune response is often compromised because of hyperglycemia, which impairs neutrophil function, including phagocytosis and migration, leading to ineffective pathogen clearance [19]. While the immune system attempts to eliminate the bacteria, the pathogens residing in the wound employ various mechanisms to evade detection and destruction. One of the most significant strategies used by many wound pathogens, such as S. *aureus and P. aeruginosa*, is biofilm formation [20]. These biofilms provide physical protection against immune cells and antimicrobial agents, making bacteria much more resistant to both innate immune responses and antibiotic treatment. Furthermore, bacteria within biofilms exhibit increased gene expression for resistance factors, such as those for antibiotics (e.g., mecA in *S. aureus* for methicillin resistance). Biofilms can also hinder the penetration of immune cells into the deeper layers of the wound, allowing the infection to persist and further complicating healing [21].

**Fig. 2:**
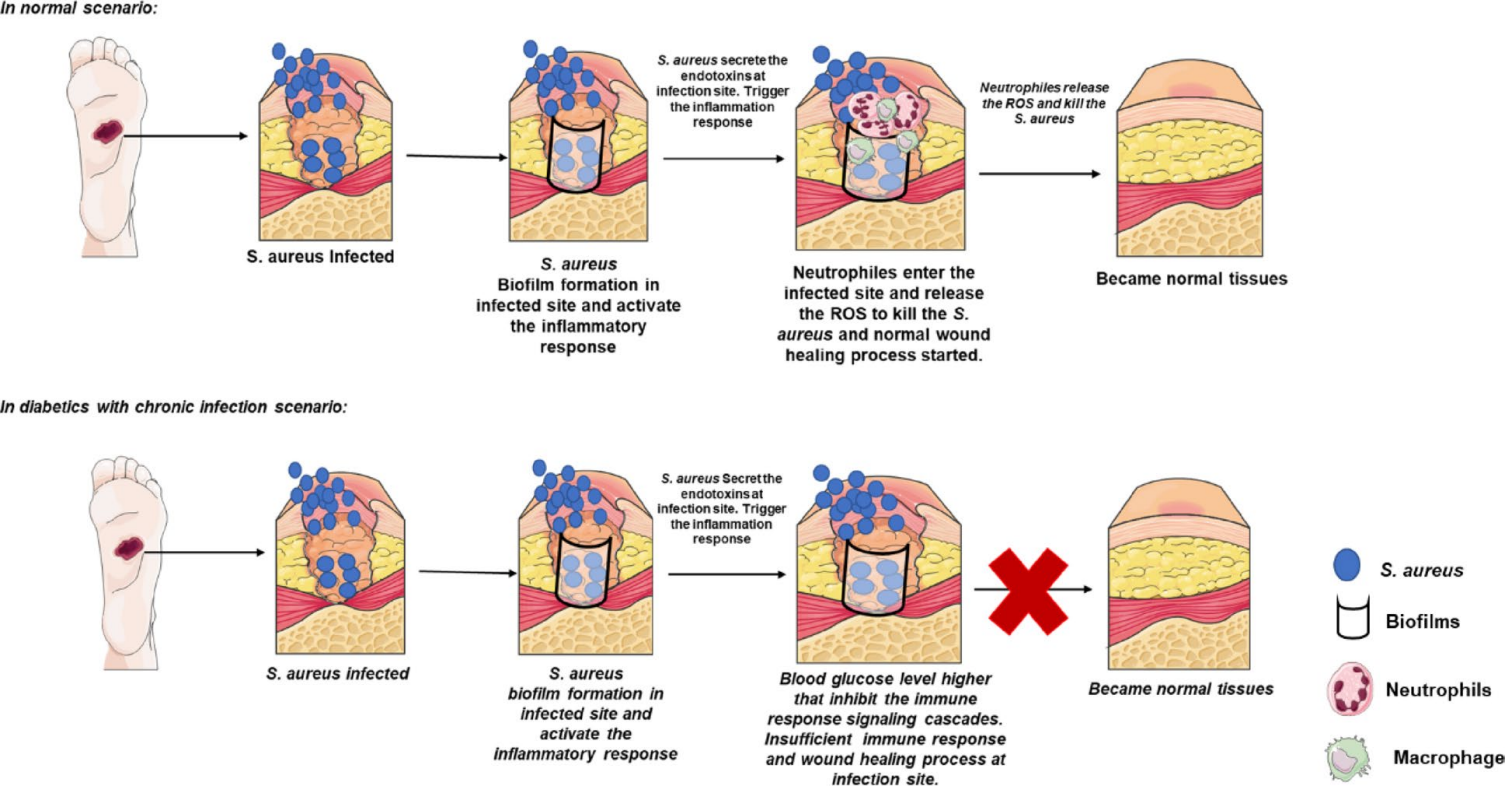
Schematic comparison of wound healing in normal versus diabetic conditions. In normal wounds, immune cells effectively clear bacteria, enabling timely tissue repair and regeneration. In diabetic wounds, persistent hyperglycemia disrupts immune signaling, enhances *S. aureus* proliferation, delays neutrophil-mediated clearance, and ultimately impairs healing

As the infection progresses, the interaction between the pathogens and host cells becomes more complex. Pathogens such as *S. aureus* utilize surface proteins (e.g., ClfA and fibronectin-binding proteins) to adhere to host cells and tissues, which is a critical step in colonization. They also secrete toxins, including hemolysins and enterotoxins, which damage host cells, facilitate tissue destruction, and trigger further inflammatory responses. Similarly, *P. aeruginosa* produces exotoxins (e.g., exotoxin A) that disrupt host cell function, leading to cell death and promoting the spread of infection [22]. In DFU, the immune response is often ineffective in containing this invasion. The presence of high glucose levels further exacerbates this interaction, as glucose serves as a nutrient for bacteria and promotes the release of inflammatory mediators, which, in turn, damage surrounding tissues, as depicted in Fig. 2. Additionally, the impaired vascularization seen in diabetes results in reduced oxygen and nutrient delivery to the wound site, preventing effective immune cell function and aiding pathogen survival [23].

As outlined above in DFUs, chronic hyperglycemia impairs innate immune responses and vascular function, leading to inefficient pathogen clearance and prolonged inflammation. This dysfunction creates a permissive environment for biofilm-forming pathogens such as *S. aureus* and *P. aeruginosa*, ultimately stalling wound healing progression [24].

#### Inflammasomes

Wound healing is a dynamic, multi-phase process encompassing inflammation, proliferation, granulation tissue formation, and remodeling, in which the early inflammatory phase is essential for initiating tissue repair. Inflammasomes, particularly the NLRP3 inflammasome, play a central role in regulating this phase by sensing both pathogen-associated molecular patterns (PAMPs) and damage-associated molecular patterns (DAMPs) released during injury, such as those generated in thermal burns. Upon activation, NLRP3 processes pro-IL-1β and pro-IL-18 into their active forms, triggering a robust inflammatory cascade that recruits immune cells to the wound site. Clinical and experimental burn models demonstrate that NLRP3 expression is markedly elevated in the acute phase, especially in deeper dermal layers, and is associated with increased IL-1β and IL-18 levels. Functional studies reveal that NLRP3 deficiency impairs wound closure, characterized by reduced collagen deposition, poor keratinization, and diminished macrophage infiltration. NLRP3 also influences macrophage polarization, ensuring timely transition from pro-inflammatory to pro-repair phenotypes. Pharmacological inhibition of NLRP3 with glyburide mimics the knockout phenotype when administered immediately post-burn, underscoring the necessity of acute phase inflammasome activation; however, delayed inhibition after the inflammatory peak preserves normal healing. These findings establish NLRP3 as a critical regulator of burn wound repair, coordinating inflammatory mediator production, macrophage recruitment, and phenotypic switching to ensure timely progression from inflammation to tissue regeneration [25]. Upon tissue disruption, danger-associated molecular patterns (DAMPs) Such as extracellular ATP, high-mobility group box 1 (HMGB1), and uric acid crystals are released from damaged cells [26, 27], while PAMPs from the wound microbiome including lipopolysaccharide (LPS) from Gram-negative bacteria, lipoteichoic acid from Gram-positive bacteria, and bacterial DNA-are detected by pattern recognition receptors (PRRs) such as NLRP3, AIM2, or NLRC4 [28]. This recognition triggers oligomerization of the sensor protein, recruitment of the adaptor protein ASC through pyrin–pyrin domain interactions, and subsequent activation of pro-caspase-1. Activated caspase-1 cleaves pro–IL-1β and pro–IL-18 into their mature, bioactive forms, which are secreted to promote local inflammation [29]. In the initial inflammatory phase, IL-1β facilitates neutrophil infiltration and macrophage activation, whereas IL-18 supports angiogenesis and epithelial cell proliferation [30].

In the remodeling phase, resolution of inflammasome activation is essential to avoid fibrosis and hypertrophic scarring. Here, microbiome-derived short-chain fatty acids (SCFAs) and anti-inflammatory metabolites can suppress NLRP3 activity by inhibiting ROS production and modulating histone acetylation, promoting tissue homeostasis [31]. Thus, inflammasome regulation in wound healing is a dynamic interplay between host molecular sensors and microbial signals, where temporal control combined with a balanced microbiome determines whether wounds progress toward regeneration or chronic inflammation.

#### TLRs signaling modulations

Toll-like receptors (TLRs) are pivotal pattern recognition receptors that detect microbial components and host-derived danger-associated molecular patterns (DAMPs) at the wound site, initiating innate immune responses essential for infection control and tissue repair. In wound infections, TLR signaling is activated by PAMPs such as lipopolysaccharides (TLR4 ligands), lipoteichoic acid (TLR2 ligands), flagellin (TLR5 ligand), and unmethylated CpG DNA (TLR9 ligand) derived from invading bacteria, fungi, or viruses [32]. Upon ligand binding, TLRs recruit adaptor proteins primarily MyD88 or TRIF which activate downstream kinases including IRAKs and TRAF6, leading to the stimulation of transcription factors NF-κB, AP-1, and IRF family members [33]. This results in the transcriptional upregulation of pro-inflammatory cytokines (IL-1β, TNF-α, IL-6) [32], chemokines (CXCL8/IL-8, CCL2), antimicrobial peptides, and co-stimulatory molecules that enhance immune cell recruitment and activation [34]. In a balanced response, this acute inflammation clears pathogens and facilitates the transition to the proliferative phase of wound healing. However, dysregulated TLR signaling either excessive or prolonged can exacerbate tissue injury, sustain inflammation, and impair healing, contributing to chronic wounds such as diabetic ulcers [35]. Conversely, insufficient TLR activation compromises pathogen clearance and predisposes wounds to persistent infection. Modulation of TLR pathways, through agonists to boost antimicrobial defense or antagonists to dampen excessive inflammation, has emerged as a therapeutic strategy. For example, TLR2/TLR4 antagonists can reduce overactive inflammation in infected wounds, while topical TLR agonists can enhance pathogen recognition and immune activation in immunocompromised conditions [36]. Additionally, wound-associated microbiota influence TLR signaling dynamics by producing metabolites and structural components that either activate or inhibit specific TLRs, shaping the local immune microenvironment [37]. Thus, precise temporal and spatial modulation of TLR signaling is critical in wound infections to achieve an optimal balance between effective microbial clearance and controlled inflammation for successful tissue repair.

#### Phagocyte dysfunction and metabolic reprogramming

Phagocyte reprogramming in wound infection refers to the dynamic alteration of innate immune cell phenotypes in response to the evolving microenvironment of an infected wound. In the early phase of infection, neutrophils and monocyte-derived macrophages are rapidly recruited to the wound bed through chemokine gradients such as CCL2, CXCL8, and CXCL10 [38]. Initially, these phagocytes adopt a pro-inflammatory, microbicidal program characterized by elevated production of reactive oxygen species (ROS), nitric oxide (NO), proteolytic enzymes, and pro-inflammatory cytokines like IL-1β, IL-6, and TNF-α, enabling efficient pathogen clearance [39]. Pattern recognition receptors (PRRs), including Toll-like receptors (TLRs) and NOD-like receptors (NLRs), sense microbial components and damage-associated molecular patterns (DAMPs), triggering activation of signaling cascades such as NF-κB, MAPK, and inflammasomes (e.g., NLRP3), which further amplify the inflammatory response [40]. As the infection resolves, persistent inflammation can be detrimental to tissue repair, and phagocytes undergo a functional reprogramming toward an anti-inflammatory, pro-resolving phenotype. This transition involves upregulation of markers such as CD206, Arg1, and IL-10, enhanced efferocytosis of apoptotic cells, and secretion of growth factors like VEGF, TGF-β, and FGF2, which promote angiogenesis, fibroblast activation, and ECM remodeling [41]. In chronic or biofilm-associated infections, however, microbial persistence and metabolic cues from the wound microbiome can skew phagocyte polarization toward a dysfunctional state either sustaining a prolonged M1-like inflammatory profile that damages tissue or inducing an immunosuppressed M2-like state that allows microbial survival [42]. Thus, successful wound healing in infection relies on a tightly regulated sequence of phagocyte activation, polarization, and metabolic adaptation, ensuring efficient pathogen elimination while enabling timely transition to tissue repair.

#### Pathogen-derived toxins and immune disruption

Pathogens that dominate chronic wounds, particularly those embedded in biofilms, play a central role in exacerbating tissue damage and maintaining a persistent inflammatory state. These microorganisms, including *S. aureus*,* P. aeruginosa*,* and S. pyogenes*, release a variety of toxins and inflammatory mediators that not only damage host tissues but also dysregulate the immune response, preventing proper wound healing. For instance, *S. aureus* produces alpha-toxin, a pore-forming toxin that inserts itself into the membranes of host cells, causing cell lysis and contributing to tissue destruction. Pore formation results in the disruption of cellular integrity, leading to cytotoxicity and the release of intracellular contents that further promote inflammation. In addition to alpha-toxin, *S. aureus* secretes a range of exoproteins and proteases, such as V8 protease and hyaluronidase, that degrade ECM components and other host tissues. These enzymes break down proteins like collagen, fibronectin, and elastin, which are essential for wound healing and tissue integrity. The degradation of these structures impairs the wound’s ability to regenerate and repair itself, further prolonging the inflammatory phase and preventing the transition to healing [43]. Moreover, the release of these toxins and enzymes stimulates the production of pro-inflammatory cytokines, such as TNF-α, IL-1β, and IL-6. These cytokines act as signaling molecules that recruit additional immune cells to the site of infection, including neutrophils and macrophages, to control the disease. However, in the context of dysbiosis, this immune response is often exaggerated and prolonged, leading to chronic inflammation as reported [44]. The sustained production of these inflammatory mediators, rather than resolving the infection, results in tissue damage and the perpetuation of an inflammatory cycle. The dysregulated immune response in chronic wounds is a consequence of an imbalance between pro-inflammatory and anti-inflammatory signals. In a typical, acute wound healing scenario, inflammation is a transient process that is resolved as part of the healing cascade, allowing tissue regeneration. However, in chronic wounds, particularly those associated with dysbiosis and biofilm formation, this inflammation becomes persistent. The overproduction of inflammatory cytokines and the continuous recruitment of immune cells, coupled with the tissue damage caused by bacterial toxins, prevent effective immune clearance of the pathogens, impair tissue repair processes, and delay the healing of the wound. The prolonged inflammatory state in chronic wounds also suppresses the function of key immune cells, including neutrophils and macrophages, by altering their cytokine profiles and impairing their ability to phagocytize and clear bacteria [45]. As a result, the wound remains in a prolonged inflammatory phase, which prevents the transition to the proliferative phase, characterized by tissue growth and wound closure. The chronic presence of biofilm-forming pathogens, combined with the sustained release of toxins and inflammatory mediators, creates an environment where the immune system is not able to resolve the infection effectively, leading to delayed wound healing, recurrent infections, and further tissue damage. Shown in Table 1.

**Table 1:** The toxins and exoproteins secreted by microorganisms in chronic wounds

Pathogenic microorganisms	Toxins/Exoproteins	Effect	Reference
*S. aureus*	Alpha-toxin	Forms pores in host cell membranes, causing cell lysis and tissue destruction.	[43]
	Exoproteins (e.g., V8 protease, hyaluronidase)	Degrades host ECM components (collagen, fibronectin), contributing to tissue breakdown.	[46]
	Enterotoxins (e.g., SEA, SEB)	Superantigens that activate T-cells nonspecifically, causing systemic inflammation and immune dysregulation.	[47]
	Protein A	Binds to the Fc region of IgG, inhibiting immune responses and promoting persistence of infection.	[48]
*P. aeruginosa*	Exotoxin A	Inhibits protein synthesis in host cells by ADP-ribosylation of elongation factor 2, leading to cell death.	
	Pyocyanin	Generates ROS that damage host tissues and immune cells, impairing the immune response.	[49]
	Elastase, Protease, Alkaline protease	Degrades host proteins, including elastin and collagen, promoting tissue damage and immune evasion.	[50]
	LPS	riggers inflammation by activating immune cells and promoting the release of pro-inflammatory cytokines.	[51]
*Streptococcus pyogenes*	Streptolysin O and S	Pore-forming toxins that cause cell lysis and contribute to tissue damage.	[52]
	Exotoxins (e.g., SPE-A, SPE-B)	Superantigens that cause systemic inflammation and immune system activation, leading to further tissue injury.	[53]
*Enterococcus faecalis*	Gelatinase and Hyaluronidase	Breaks down host ECM and promotes bacterial invasion.	[54]
*Escherichia coli*	Hemolysin (HlyA)	Causes cell membrane disruption by forming pores, leading to cell lysis.	[55]
	LPS	Promotes inflammation and immune cell activation through the TLR4 receptor.	[56]
*Clostridium perfringens*	Alpha-toxin	Phospholipase enzyme that breaks down host cell membranes, leading to tissue necrosis.	[57]
	Theta-toxin	Forms pores in host cell membranes, contributing to tissue damage and bacterial survival.	[58]
*Klebsiella pneumoniae*	K1 capsular polysaccharide	Inhibits phagocytosis by immune cells and enhances bacterial survival.	[59]
*Proteus mirabilis*	Urease	Raises the pH in the wound environment, favoring bacterial survival and promoting tissue damage.	[60]
*Bacteroides fragilis*	Bacteriocins	Inhibit host immune function and contribute to tissue degradation.	[61]
*Acinetobacter baumannii*	Outer membrane vesicles (OMVs)	Facilitate immune evasion by delivering virulence factors, including proteases and toxins, to host cells.	[62]
	Beta-lactamase	Inactivates beta-lactam antibiotics, contributing to antibiotic resistance.	[63]
*Fusobacterium necrophorum*	Leukotoxin	Kills immune cells, particularly neutrophils and macrophages, impairing immune responses and promoting infection.	[64]
*Prevotella spp.*	Proinflammatory proteases	Degrade host proteins and stimulate the production of inflammatory cytokines, perpetuating chronic inflammation.	[65]
*Stenotrophomonas maltophilia*	Metalloproteases	Degrade host tissue proteins and promote bacterial colonization in chronic wounds.	[66]
*Rhodococcus equi*	Virulence-associated proteins (VapA, VapB)	Modulate host immune response and promote bacterial survival within macrophages.	[67]
*Vibrio vulnificus*	Cytolysins (e.g., VvhA)	Disrupts host cell membranes, contributing to tissue necrosis and enhancing bacterial spread.	[68]

### DFU: pathogenesis and Microbiome interactions

DFUs are a leading example of chronic wounds, arising from neuropathy, impaired vascularization, and persistent hyperglycemia. High glucose levels not only impair neutrophil and macrophage function but also provide a nutrient-rich environment for pathogens, creating a permissive niche for infection. As a result, DFUs frequently harbor polymicrobial biofilms dominated by *S. aureus*, *P. aeruginosa*, *Enterococcus faecalis*, and *E. coli*, which protect pathogens from both immune clearance and antibiotics. Dysbiosis is a hallmark of DFUs. Beneficial commensals are depleted, while opportunistic species expand, altering local immune surveillance and perpetuating chronic inflammation. The disrupted microbiome interacts with host immunity through mechanisms such as impaired TLR signaling, persistent inflammasome activation, and excessive protease release, all of which degrade extracellular matrix and hinder re-epithelialization. Therapeutically, these insights underscore the need for microbiome-responsive strategies. Emerging nanoplatforms aim to selectively disrupt pathogenic biofilms while sparing or even supporting beneficial microbes. Phage-based nanocarriers, probiotic-loaded hydrogels, and antimicrobial peptides integrated into smart biomaterials represent promising avenues to restore microbial balance, suppress infection, and promote tissue regeneration in DFUs [69].

### Smart nanomaterials for microbiome-targeted therapy

Nanomaterials possess unique features such as high surface area-to-volume ratio, tunable surface chemistry, and controlled release properties, making them powerful tools in infection control and microbiome-targeted therapies. Their nanoscale interactions enable precise drug delivery, selective eradication of pathogens, and modulation of microbial communities capabilities that conventional antibiotics often lack [70]. In the context of chronic wounds and dysbiosis, smart nanomaterials provide novel opportunities to both disrupt biofilms and restore microbial balance, thereby promoting tissue regeneration.

Gold nanoparticles (AuNPs) exemplify this potential due to their biocompatibility and facile surface functionalization. Functionalized AuNPs can be conjugated with antimicrobial peptides or antibodies to selectively target biofilm-forming pathogens such as *Staphylococcus aureus* and *Pseudomonas aeruginosa*, reducing off-target cytotoxicity while sparing commensal microbes [71]. Similarly, liposomes lipid bilayer vesicles widely used in drug delivery can encapsulate antibiotics or quorum-sensing inhibitors, protecting them from degradation and enabling site-specific release in infected wound niches. Liposomal formulations have also been explored for delivering probiotics or microbiome-modulating metabolites, underscoring their dual role in infection control and microbiome restoration.

Carbon-based nanomaterials, including carbon dots (C-dots), graphene derivatives, and multiwalled carbon nanotubes (MWCNTs), add another layer of functionality. C-dots generate ROS under photoactivation, enabling photodynamic disruption of biofilms while minimizing systemic effects [72, 73]. Graphene oxide and MWCNTs have shown promise in wound healing applications by regulating inflammatory signaling and reducing colonization by pathogenic bacteria in diabetic ulcers. Their tunable surfaces also allow for selective functionalization to target microbial virulence pathways rather than causing broad-spectrum killing, an approach that aligns with microbiome-sensitive therapy [74]. At the same time, graphene has shown promise in biosensing and diagnostic applications due to its excellent electronic properties [75].

Metallic nanoparticles including silver, zinc oxide (ZnO), titanium dioxide (TiO₂), and copper nanoparticles remain central to nanomedicine owing to their intrinsic antimicrobial activity. Silver nanoparticles (AgNPs) act through multiple mechanisms: releasing Ag⁺ ions that disrupt bacterial membranes, generating ROS, and binding thiol groups in enzymes to block essential metabolic processes [76]. ZnO and TiO₂ nanoparticles induce oxidative stress and membrane damage, while copper nanoparticles release Cu²⁺ ions that impair amino acid synthesis and damage nucleic acids [77, 78]. Importantly, these nanoparticles are especially effective against biofilm-associated pathogens in chronic wounds, where conventional antibiotics often fail. By carefully controlling surface modifications and dosages, metallic nanomaterials can be tuned to reduce collateral damage to commensal bacteria, supporting microbiome preservation alongside pathogen clearance [79].

Beyond their antimicrobial roles, smart nanomaterials are increasingly being designed to reprogram host–microbiome interactions. Functionalized nanoparticles can bind LPS to modulate TLR4 signaling, thereby dampening excessive inflammation while promoting M2 macrophage polarization and tissue repair. Beyond their antimicrobial roles, smart nanomaterials are increasingly being designed to reprogram host–microbiome interactions. Functionalized nanoparticles can bind LPS to modulate TLR4 signaling, thereby dampening excessive inflammation while promoting M2 macrophage polarization and tissue repair. Similarly, nanoparticle-induced ROS can activate NLRP3 inflammasomes, enhancing phagocytic clearance of pathogens. Another promising strategy involves engineering nanoparticles to interfere with bacterial quorum sensing, thereby reducing virulence factor expression without causing widespread microbial eradication. These immunomodulatory and microbiome-sensitive strategies represent a paradigm shift moving from indiscriminate pathogen killing to restoring ecological balance and promoting healing. Taken together, the integration of metallic, carbon-based, and lipid nanoplatforms with immune-modulatory designs highlights the versatility of smart nanomaterials in addressing chronic infections.

#### Mechanisms of selective nanotherapy

The concept of microbiome-friendly nanomaterials is built on the principle of selectivity, where the nanomaterial is designed to specifically interact with pathogenic bacteria while avoiding or minimally affecting beneficial bacteria. Several strategies have been explored to achieve this selectivity, as shown in Table 2:

**Table 2:** Selectivity of nanomaterials targeting harmful bacteria

Strategy	Mechanisms of action	Targeted bacteria	Nanomaterial	Advantage	Reference	
Surface Functionalization with Targeting Ligands	Functionalizing nanomaterials with specific ligands (e.g., antibodies, peptides) that bind to surface markers unique to pathogenic bacteria.	*S. aureus*, *P. aeruginosa*	AuNPs	High specificity, reduces collateral damage to beneficial bacteria	[80]	
pH-Sensitive Nanomaterials	Nanomaterials activated in acidic environments, typical of infection sites, allowing for controlled release of antimicrobial agents.	*Helicobacter pylori*, *and P. aeruginosa*	Chitosan NPs, polymeric NPs	Targeted release at infection sites, minimal impact on healthy microbiome	[81]	
Bacteriophage-based nanomaterials	Incorporating bacteriophages into nanomaterials to target specific pathogenic bacteria, leaving beneficial bacteria unaffected.	MRSA, *E. coli*, *Salmonella spp.*	Phage-functionalized NPs,	Selective targeting of pathogenic bacteria, reduces resistance development	[82]	
Enzyme-responsive nanomaterials	Nanomaterials that release antimicrobial agents upon interaction with bacterial-specific enzymes.	*Clostridium difficile*, *S. aureus*	Lipid-based NPs, chitosan NPs	Enzyme-triggered activation, reduced systemic effects	[83]	
Antibody-functionalized nanomaterials	Nanomaterials conjugated with antibodies to bind to and neutralize pathogenic bacteria specifically.	*Salmonella spp.*, *E. coli*	Polymeric NPs, carbon-based nanomaterials	High specificity, targeted pathogen neutralization	[84]	
Magnetic NPs	Magnetic nanomaterials that can be targeted at infected tissues using an external magnetic field, selectively concentrating at infection sites.	*S. aureus*, *E. coli*	Iron oxide NPs (Fe_3_O_4_-NPs) magnetic nanocomposites	Enhanced targeting using magnetic fields, minimal side effects	[85]	
AgNPs with peptide conjugation	AgNPs are conjugated with antimicrobial peptides to enhance selective toxicity toward pathogenic bacteria.	*S. aureus*, *E. coli*, *P. aeruginosa*	AgNPs, peptide-functionalized NPs	Synergistic antibacterial effect, selectivity due to peptide targeting	[86]	
Surface charge modification	Modifying the surface charge of NPs to selectively interact with the membranes of pathogenic bacteria while avoiding beneficial bacteria	*P. aeruginosa*	polymeric NPs, silica NPs	Increased selectivity based on charge interactions, less impact on beneficial microbiota	[87]	

**Fig. 3:**
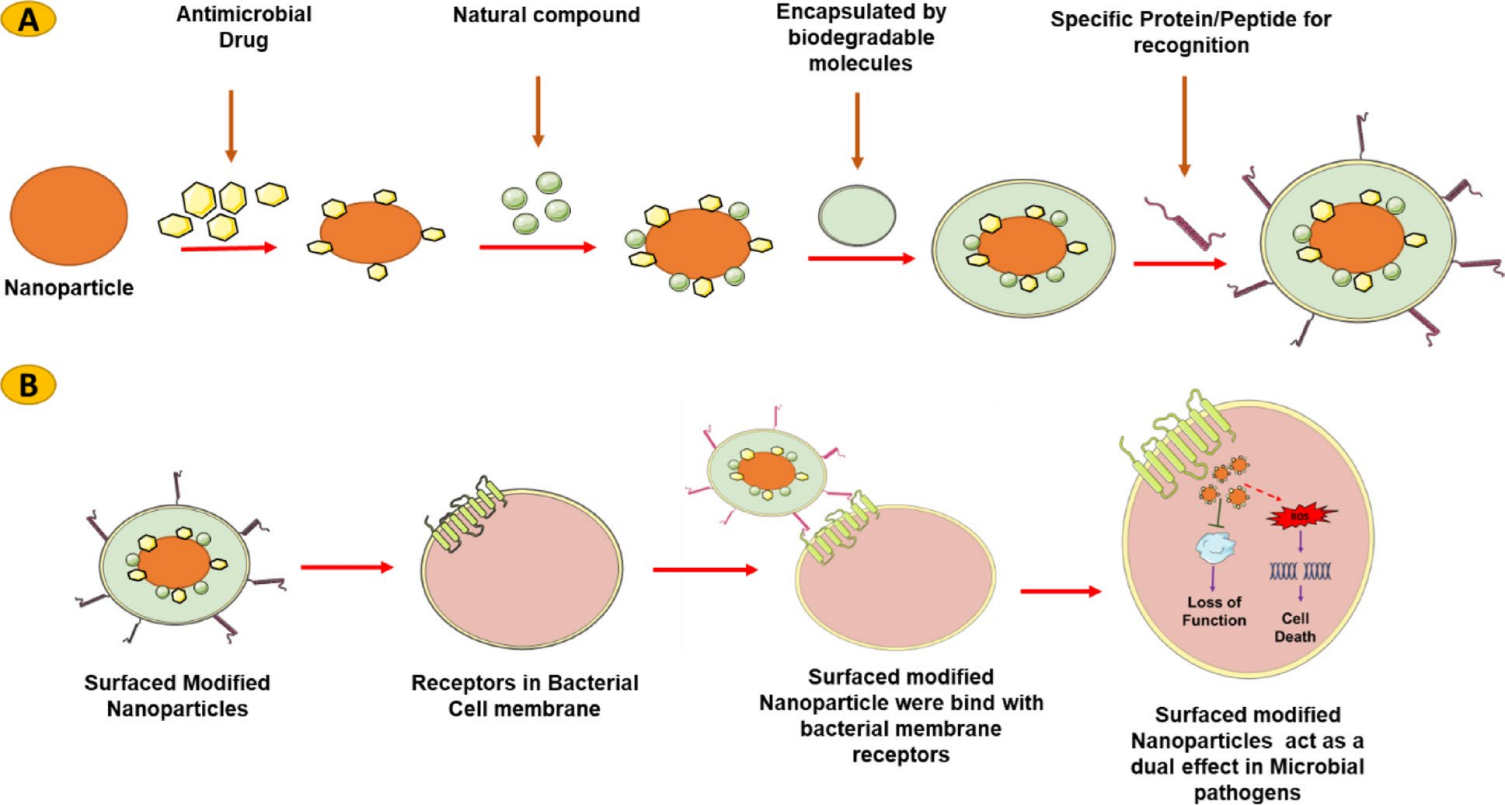
**A**) Surface functionalization of synthesized NPs with various active compounds. The surface of NPs was modified by incorporate antimicrobial drugs and natural compounds, enhancing their antimicrobial activity. These NPs were encapsulated and bridged with natural biomolecules, functionalized with proteins or peptides, enabling them to recognize pathogen-specific receptors. **B**) Mechanisms of surface-modified NPs in combating drug-resistant pathogens. Proteins or peptides on the NPs facilitate their entry into the cell membrane. Active compounds, including antibiotics and natural compounds, inhibit virulence factors and essential proteins in pathogens, leading to the loss of their functionality. Metal or metal oxide-based NPs are highly reactive and induce ROS, mediating cell death

One of the most effective approaches to ensuring selectivity is functionalizing nanomaterials with specific ligands that bind only to certain bacterial species or strains. These ligands can be proteins, peptides, antibodies, or other biomolecules that have a high affinity for surface markers or receptors that are uniquely expressed by harmful bacteria, as shown in Fig. 3. For instance, pathogenic bacteria often express specific surface proteins, LPS, or carbohydrates that are absent in beneficial microbiota. By conjugating nanomaterials with ligands that recognize these unique bacterial markers, researchers can ensure that the nanomaterials selectively bind to and disrupt harmful bacteria without affecting healthy microorganisms. For example, biotinylated *anti-S. aureus* polyclonal antibody-functionalized NPs can target specific pathogens such as *S. aureus* or *E. coli* by binding to surface antigens that are overexpressed on these bacteria but not commensal organisms. This strategy offers a high degree of specificity and minimizes the impact on the microbiome [88].

#### pH-responsive and enzyme-responsive platforms

On the other hand, pH-sensitive nanomaterial- The microenvironment of pathogenic infections often differs significantly from that of the surrounding tissue. Infections typically occur in sites where the pH is lower (e.g., inflamed tissues, abscesses), whereas healthy tissues tend to have a more pH in neutral. Nanomaterials can be engineered to be pH-sensitive, allowing them to release antimicrobial agents or become active only in acidic environments [89]. This approach ensures that the nanomaterial will be activated primarily in the vicinity of pathogenic bacteria, which thrive in such environments and will remain inactive in the more neutral pH of healthy tissues, pH-responsive NPs are commonly made using materials like chitosan, which undergoes protonation in acidic conditions, leading to a release of encapsulated drugs or antimicrobial agents at infection sites. This selective activation ensures that the nanomaterials target pathogens without significantly affecting the microbiome in other parts of the body.

Some nanomaterials are specifically engineered to be activated by enzymes that are overexpressed in pathogenic bacteria. Certain enzymes are uniquely associated with bacterial cell walls or metabolic pathways and are absent or expressed at negligible levels in healthy microbiota. By designing nanomaterials responsive to these enzymes, it is possible to achieve selective activation and targeted antimicrobial delivery. In such enzyme-triggered release systems, the nanomaterials are coated or functionalized with substrates that can be selectively cleaved by bacterial enzymes. This cleavage event initiates the release of the encapsulated antimicrobial payload, ensuring activation only in the presence of pathogenic microorganisms. For instance, Li and co-workers reported supramolecular gelatinase-responsive NPs (Van⊂SGNPs@RBC) as an effective antibacterial platform against Gram-positive and gelatinase-positive pathogenic bacteria. In this system, vancomycin is encapsulated within gelatin-based NPs and camouflaged with red blood cell membranes to enhance biocompatibility and immune evasion. Upon reaching the infection site, the overexpressed bacterial gelatinase degrades the gelatin core, triggering the controlled release of vancomycin and enabling site-specific antibacterial action [90]. Another example involves esterase-degradable antimicrobial nanosponges, which are engineered by polymer-stabilizing self-assembled cationic poly(oxanorborneneimide) (PONI) scaffolds bearing guanidine, maleimide, and tetraethyleneglycol monomethyl ether (GMT) groups around essential-oil cores, followed by crosslinking with dithiol-disulfide (DTDS). The resulting nanosponge network is susceptible to degradation by endogenous biomolecules, including glutathione and esterase enzymes, enabling a triggered structural breakdown that releases the antimicrobial payload at the infection site. These nanocomposites demonstrate potent activity against both Gram-negative and Gram-positive bacterial biofilms, while exhibiting no detectable cytotoxicity toward mammalian fibroblast cells [91].

#### Bacteriophage mediates combat multi drug resistance pathogens

A promising approach for developing microbiome-friendly nanomaterials involves the use of bacteriophages viruses that specifically infect bacteria. Bacteriophages are inherently selective, targeting specific bacterial strains while leaving the beneficial microbiota largely unaffected. Incorporating bacteriophages into nanomaterials, such as functionalized NPs, enables particular antimicrobial activity without disrupting commensal microorganisms. Phage-display technology has been utilized to engineer nanomaterials capable of delivering bacteriophages directly to pathogenic bacteria, thereby increasing therapeutic efficiency and minimizing collateral damage to beneficial microbes [92].

A receptor-guided complementarity group (CG) strategy has been proposed to design broad-spectrum phage cocktails by combining phages that target distinct bacterial surface receptors, such as the type IV pilus, O-specific antigen/lipopolysaccharide (OSA/LPS), or flagella. This approach reduces the risk of simultaneous resistance development. In *P. aeruginosa*, a triple-phage cocktail (KIM-C1) comprising phages from distinct CGs achieved ≥ 96% killing efficacy against diverse clinical isolates, including biofilm-associated strains, and demonstrated enhanced antibacterial activity when combined with aztreonam in both in vitro assays and murine wound infection models. Similarly, in *S. aureus*, a CG-informed cocktail (KIM-C4) administered with vancomycin exhibited broad-spectrum activity. Both species-specific cocktails could be co-administered to effectively treat polymicrobial infections without antagonistic interactions [93]. In another study, Kunisch et al. reported that evolved lytic phages FJK.R9-30 and MK.R3-15, developed through a 30-passage in vitro biofilm evolution assay against MDR *P. aeruginosa*, displayed an expanded host range and enhanced antibiofilm activity [94]. The resulting two-phage cocktail effectively suppressed planktonic bacterial growth for up to seven days and significantly reduced biofilm biomass without the emergence of dual resistance. Interestingly, resistance to one phage increased susceptibility to the other, while resistant mutants exhibited reduced virulence and impaired biofilm formation. Additionally, Lu et al. 2021 demonstrated that the phage-derived endolysin LysP108 exhibited potent antibacterial activity against Gram-positive bacteria both in vitro and in vivo. Notably, combining LysP108 with antibiotics enhanced its therapeutic effect, suggesting a promising strategy to address the growing problem of drug resistance in Gram-positive pathogens [95].

### Microbiome as a dynamic bio-indicator

Recent advancements in microbiome research have demonstrated that disruptions to this microbial balance, are associated with various diseases, including gastrointestinal disorders, autoimmune diseases, metabolic conditions, and even mental health issues. With the increasing understanding of the microbiome’s role in health and disease, there has been growing interest in leveraging microbiome profiles as bio-indicators to optimize therapeutic strategies. By feeding real-time microbiome data into AI systems, healthcare practitioners could dynamically adjust and personalize treatment regimens, improving therapeutic outcomes and reducing adverse effects.

The microbiome is not just a static collection of microbes; it is a dynamic ecosystem that can shift in response to environmental factors, diet, stress, and the use of medications like antibiotics. These shifts in microbial composition can influence how the body responds to treatments, such as chemotherapy, antibiotics, and immunotherapy. For instance, a healthy, balanced microbiome can enhance immune responses and help mitigate the side effects of treatments, while an imbalanced microbiome may exacerbate the toxicity or reduce the efficacy of the same therapies. Therefore, understanding and monitoring an individual’s microbiome profile can offer deep insights into their overall health and their likely responses to various treatments [96].

**Fig. 4:**
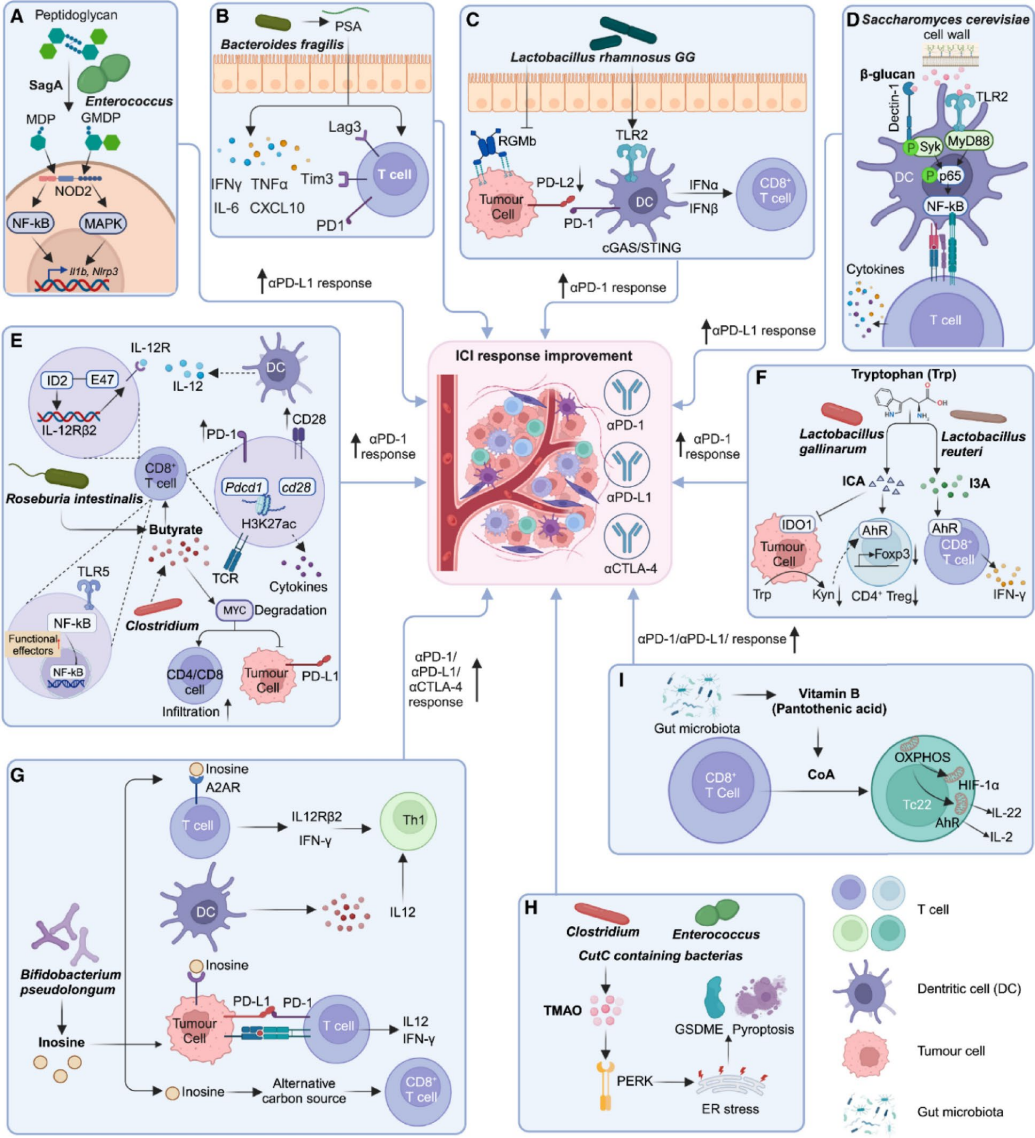
Mechanistic insights into how the gut microbiome and its metabolites modulate cancer immunotherapy outcomes. Specific microbes (*Enterococcus*, *Bacteroides fragilis*, *Lactobacillus rhamnosus GG*, *Saccharomyces cerevisiae*, *Roseburia intestinalis*, *Clostridium*, *Lactobacillus gallinarum, L. reuteri*, *Bifidobacterium pseudolongum*, etc.) and their metabolites (butyrate, inosine, indole derivatives, TMAO, vitamin B5) regulate dendritic cell activation, T-cell function, and PD-1/PD-L1 signaling, thereby enhancing immune checkpoint inhibitor (ICI) responses (anti-PD-1, anti-PD-L1, anti-CTLA-4). Key mechanisms include: (**A**) Enterococci SagA-derived MDP/GMDP activating NF-κB/MAPK; (**B**) B. fragilis PSA promoting cytokine secretion and immune checkpoints; (**C**) L. rhamnosus GG inducing IFNs and reducing PD-L2/RGMb; (**D**) yeast β-glucan activating DCs via Dectin-1/Syk & TLR2/MyD88; (**E**) butyrate modulating IL-12, TCR signaling, NF-κB, MYC, and PD-L1; (**F**) L. gallinarum ICA inhibiting IDO1/Kyn–AhR–Treg axis, L. reuteri I3A driving IFN-γ+ CD8+ T cells via AhR; (**G**) B. pseudolongum inosine activating A2AR, promoting Th1 differentiation, and supporting CD8+ metabolism and neoantigen recognition; (**H**) microbial TMAO inducing ER stress and pyroptosis via PERK–GSDME; (**I**) vitamin B5 enhancing OXPHOS to drive CD8+ Tc22 differentiation. Adapted from [105]

Advances in metagenomic sequencing have allowed researchers to catalog the microorganisms present in the human microbiome at unprecedented depth, making it possible to map the microbial fingerprints associated with different health states and diseases. These microbial signatures can be used to not only predict disease risk but also forecast the likelihood of treatment success, offering a new paradigm in precision medicine. For example, a patient with cancer might have their microbiome analyzed to determine which microbial communities are present and how they correlate with immune function or drug metabolism. Preclinical studies have demonstrated that specific gut microbial populations significantly impact the effectiveness of therapies for chronic infections and immune regulation. For example, mice with depleted gut microbiota-achieved through germ-free housing or antibiotic treatment exhibited reduced responses to CTLA-4 blockade immunotherapy. Remarkably, the immune response could be restored by reintroducing specific bacterial strains such as *Bacteroides fragilis*, either alone or in combination with *Bacteroides thetaiotaomicron* or *Burkholderia cepacian* [97]. Further evidence underscores the role of bacterial strains isolated from immune-treated tumors, such as *Bifidobacterium pseudolongum*, *Lactobacillus johnsonii*, and *Olsenella* species, which enhanced the efficacy of immune checkpoint inhibitors (ICIs) in preclinical models. Among these, *Bifidobacterium* strains improved immune cell function and promoted tumor infiltration, demonstrating their ability to augment therapeutic responses [98, 99]. Additionally, strains like *Lactobacillus paracasei* SH2020 and *Lactobacillus kefiranofaciens* ZW18 have been shown to enhance the outcomes of immunotherapies further,, suggesting their potential role in addressing chronic infections and inflammatory conditions (Fig. 4) [100, 101]. Emerging evidence indicates that specific microbial species, such as *Akkermansia muciniphila* and *Faecalibacterium prausnitzii*, influence tissue repair by modulating the immune microenvironment. These bacteria not only aid in controlling infection but also support tissue regeneration by reducing inflammation and promoting cell proliferation. Leveraging these microbial properties through advanced therapeutic platforms could significantly enhance regenerative processes in chronic wound healing and other tissue-related disorders.

Microbial metabolites have emerged as critical modulators of immune responses, influencing therapeutic outcomes. For instance, trimethylamine N-oxide (TMAO), a metabolite derived from gut microbial activity, has demonstrated the ability to stimulate immune activation and enhance the efficacy of immune checkpoint blockade (ICB) in mouse models of pancreatic cancer. butyrate, a short-chain fatty acid produced by *Roseburia intestinalis*, has been associated with improved anti-PD-1 efficacy against colorectal cancer (CRC) in mouse models [102]. Similarly, other members of the *Clostridiales* order, such as *Eubacterium hallii*, *Faecalibacterium prausnitzii*, and *Anaerostipes caccae*, have demonstrated the ability to boost CD8 + T cell activation and facilitate their infiltration into tumors, thereby enhancing the therapeutic response in solid tumors [103]. Additionally, indole-3-carboxylic acid (ICA), a metabolite derived from *Lactobacillus gallinarum*, has been shown to improve anti-PD-1 efficacy in mouse models with different microsatellite instability (MSI) statuses [104]. These findings highlight the critical role of microbial metabolites in modulating immune responses and improving the efficacy of checkpoint blockade therapies. Such findings underscore the significance of microbial metabolites as potent bioactive molecules that can be harnessed to boost immunotherapeutic strategies and support tissue regeneration in chronic diseases.

The next frontier in microbiome research is real-time microbiome profiling. With the advent of portable sequencing technologies, wearable devices, and high-throughput sequencing platforms, it is now possible to monitor an individual’s microbiome in real-time. These technologies could continuously track fluctuations in microbial populations, offering valuable feedback on the state of the microbiome and how it is responding to external stimuli such as medication, dietary changes, or environmental factors. Real-time data collection can be handy in chronic disease management, where constant adjustments to treatment are often required. For example, in the case of patients undergoing cancer treatment, real-time microbiome monitoring can provide feedback on how chemotherapy or immunotherapy is impacting the gut microbiome. If the treatment causes a detrimental shift in microbial composition, leading to adverse effects like gastrointestinal distress or immune suppression, healthcare providers could intervene by adjusting the treatment regimen or introducing probiotic interventions to restore microbial balance. Such dynamic adjustments, driven by real-time microbiome data, offer the potential to optimize therapeutic outcomes and minimize the adverse effects of treatment.

Critically, though dysbiosis worsens chronic inflammation by promoting pathogen dominance and biofilm tenacity, emerging data indicate that recapitulating microbial balance through targeted interventions may curtail AMR; yet, translating in vitro microbiome modulation to in vivo success remains challenging due to inter-individual differences in microbial assemblages. Moreover, the interaction between hyperglycemia-driven immune impairment and hypoxic niches highlights a vicious cycle for pathogen recalcitrance, yet prevalent therapies fail to account for this multifactorial etiology, requiring integrated strategies that target both microbial and host determinants for durable outcomes.

## Smart nanomaterials in infection control

**Fig. 5:**
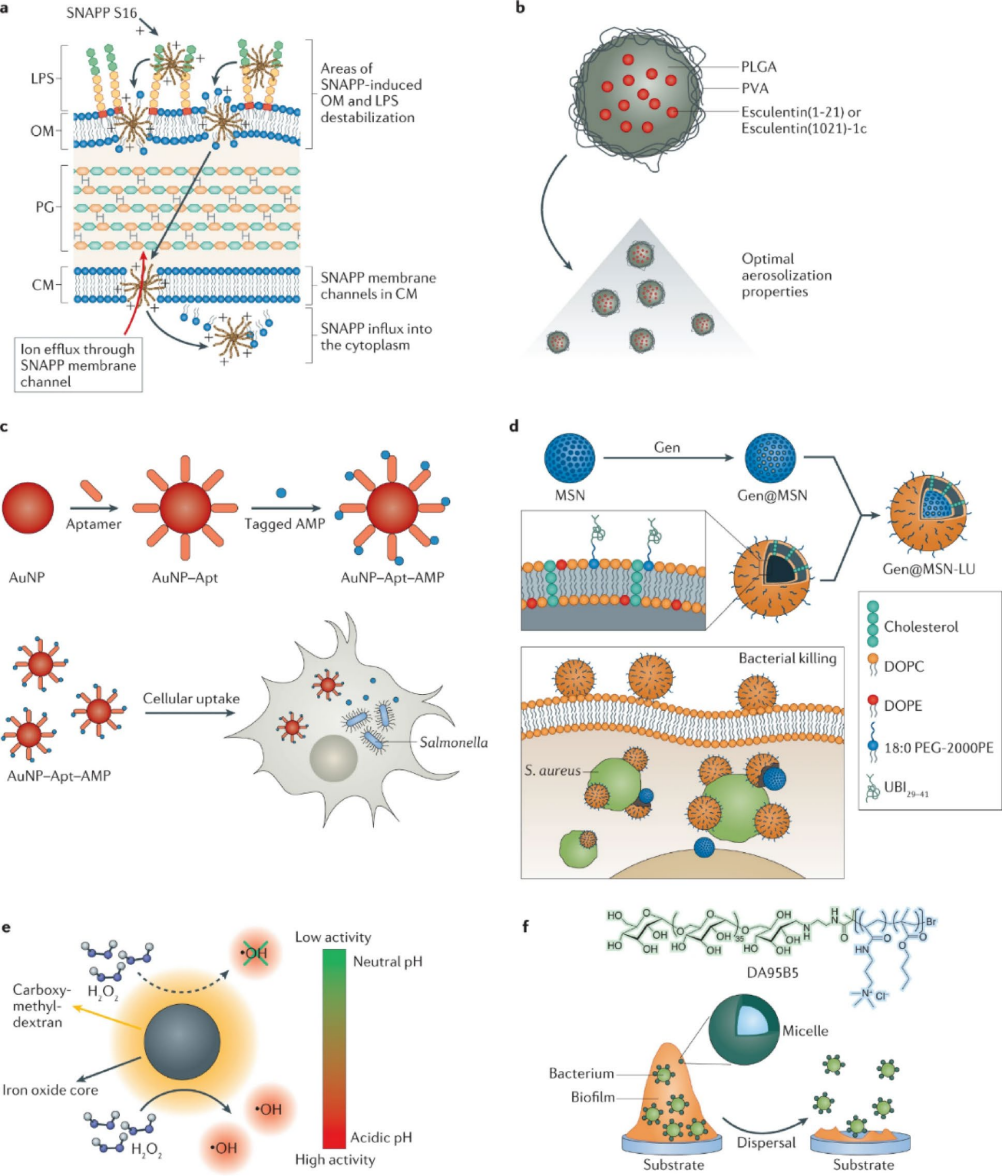
Nanomaterial-based strategies can be used to combat planktonic bacterial infections. These include SNAPP-induced membrane destabilization, polymeric NP delivery, aptamer–AMP conjugates, silica-based antibiotic carriers, ROS-generating iron oxide nanozymes, and micellar biofilm dispersal systems. This figure adapted from [107]

Sustainability has also emerged as a central theme in modern biomedical innovation that are biocompatible, biodegradable, and environmentally responsible, minimizing ecological harm while maintaining therapeutic efficacy. In sharp contrast to the environmental disadvantages of conventional synthetic metal-based alternatives, this section promotes smart green nanomaterials as environmentally friendly platforms that not only fight infections but also Support microbial balance and tissue regeneration, in Light of the microbiome disruptions discussed in the previous section regarding chronic wounds. Given the central role of microbiota in the persistence and recurrence of infections, there is a growing need for therapeutic platforms that not only offer Antimicrobial efficacy but also minimize toxicity and environmental burden. Nanotechnology has enabled the development of nanomaterials, typically 1–100 nm in size, which offer high surface area, tunable reactivity, and efficient drug delivery capabilities. Green nanomaterials, in particular, present a sustainable alternative to synthetic counterparts due to their biodegradability, biocompatibility, and eco-friendliness. Unlike conventional antibiotics, these nanomaterials often target pathogens via non-traditional mechanisms such as membrane disruption, oxidative stress, and metal ion release, making them effective even against multidrug-resistant organisms. As illustrated in Fig. 5, diverse nanomaterial-based strategies have been developed to combat planktonic bacterial infections, ranging from SNAPP-induced membrane destabilization and polymeric nanoparticle drug carriers to aptamer–antimicrobial peptide conjugates, ROS-generating nanozymes, and micellar systems that disperse biofilms [106, 107]. Key elements of sustainable materials include: (i) biodegradability to prevent environmental accumulation, (ii) biocompatibility for safe interaction with biological tissues, (iii) green synthesis using renewable resources, and (iv) low toxicity and minimal bioaccumulation in ecosystems.

Among these, chitosan a naturally derived polycationic biopolymer from chitin, has attracted attention for its potent antibacterial and antifungal properties. Its positively charged surface allows interaction with the negatively charged bacterial membranes, leading to membrane destabilization and cell lysis. These properties, combined with its mucoadhesive nature, make it suitable for wound healing, tissue engineering, and drug delivery applications [108]. Similarly, nanocellulose (NC), isolated from plant and bacterial sources, exhibits exceptional mechanical strength, hydrophilicity, and biocompatibility. NC scaffolds and hydrogels not only promote wound Healing and patient comfort but also serve as platforms for 3D bioprinting due to their high surface area and porosity [109]. Additionally, materials derived from algae, like fucoidan, have been shown to possess intrinsic antibacterial and antiviral properties.

Green nanomaterials can be synthesized via biological or “green” routes using microorganisms such as bacteria, fungi, algae, and plant extracts, which act as reducing and stabilizing agents [110, 111]. Silver nanoparticles (AgNPs) derived from plant extracts are considered sustainable because they are synthesized using biological reducing agents such as phytochemicals rather than hazardous chemicals [112]. This green synthesis approach reduces energy consumption, avoids toxic byproducts, and enhances biocompatibility and antimicrobial efficacy. Fucoidan, an algal polysaccharide, is particularly noted for its antiviral and antibacterial activity and is being explored for use in anti-infective coatings [113].

Despite their therapeutic potential, conventional metal-based nanomaterials (e.g., silver, gold, titanium dioxide) are considered environmentally unsustainable due to their persistence, poor biodegradability, and potential toxicity [114]. These materials can accumulate in aquatic and terrestrial ecosystems, disrupt microbial communities, and interfere with nutrient cycling [115, 116]. These materials are difficult to degrade naturally and may accumulate in aquatic or terrestrial ecosystems, posing risks to microbial communities, plants, and animals. These concerns are supported by recent research. For example, it has been demonstrated that AgNPs can accumulate in aquatic environments and disrupt microbial nitrogen cycling, with bioaccumulation factors (BCF) in algae exceeding 100 [117]. Meanwhile, titanium dioxide NPs have been shown to exhibit long-term ecotoxicity by causing oxidative stress in soil microbes, which can result in a 50% reduction in enzymatic activity [116]. Chronic exposure to non-biodegradable carbon nanotubes has been associated with pulmonary inflammation in humans through activation of the NLRP3 inflammasome [118]. The nanomaterils disposal through medical waste or biosolids can lead to contamination of soil and water, affect nutrient cycling, and disrupt ecological balances. For example, AgNPs can accumulate in aquatic ecosystems, impacting essential microbial communities, while titanium dioxide NPs are known to leach into water systems, contributing to long-term pollution [119, 120].

In terrestrial systems, non-biodegradable nanomaterials can disrupt the enzymatic functions of beneficial soil microbes and interfere with plant-mycorrhizal interactions. Carbon nanomaterials like graphene oxide have been shown to affect nitrogen fixation and organic matter decomposition [121, 122]. Metal oxides such as ZnO and CuO impair microbial enzymes vital for nutrient cycling, while AgNPs may reduce the population of beneficial bacteria in wastewater treatment, compromising efficiency [117]. The impact extends to aquatic life as well ZnO and CuO NPs release metal ions (Zn²⁺ and Cu²⁺), disrupting homeostasis in aquatic organisms. ZnONPs affect zebrafish embryonic development, and AgNPs have been shown to reduce ovarian size in Drosophila [123]. Furthermore that Zinc oxide NPs (ZnONPs) can negatively impact zebrafish embryos, resulting in abnormal development and increased death rates [124]. In another study exposure of AgNPs to Drosophila reduced ovary size and fecundity, fertility and gender ratios of the offspring were unaffected [125]. Earthworms exposed to TiO₂ NPs lose reproductive rhythm, a critical ecological function [126]. Additionally, carbon-based NPs may shorten lifespans in pollinators, affecting plant biodiversity [117].

### Bioaccumulation and biomagnification

**Fig. 6:**
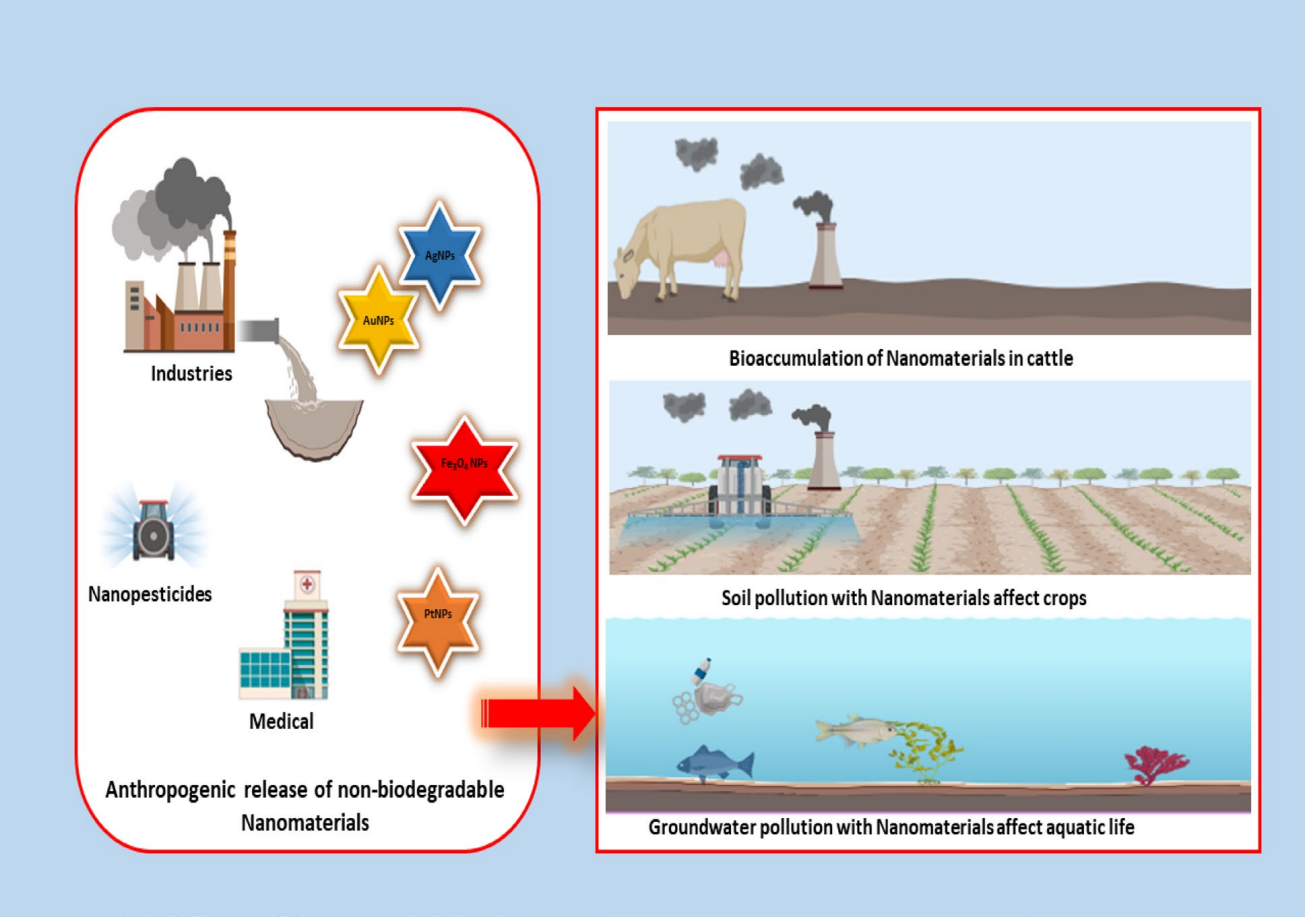
Environmental concerns associated with the anthropogenic release of non-biodegradable nanomaterials. Industrial discharge, nanopesticides, and medical applications contribute to the release of NPs (AgNPs, AuNPs, Fe₃O₄ NPs, PtNPs) into the environment. These pollutants bioaccumulate in cattle, contaminate soil and crops, and leach into groundwater, ultimately affecting aquatic life

Non-biodegradable NPs can accumulate in species’ bodies, causing biomagnification in the food chain as shown in Fig. 6. The increasing buildup of these chemicals complicates top predatory animals’ health and reproductive adaptability. Bioaccumulation is harmful not only to animals but also to humans who consume contaminated food sources. It has been claimed that NPs may cross the placenta and result in poor fetal development. Experiments conducted on several animal embryogenesis models, including rodents and zebrafish, validated the transference of NPs (TiO_2_, Au, SiO_2_, carbon, and quantum dots) from mother to fetus via the placenta [127]. Nanomaterials, particularly silver and titanium dioxide NPs, have been shown to accumulate in the liver and kidneys of fish, negatively affecting their physiologic functioning and increasing the mortality rate. Furthermore, research has shown that bioaccumulated nanomaterials may alter endocrine action in higher trophic-level species, leading to reproductive failures and population reductions. Consumption of contaminated seafood may pose serious health concerns to people, including neurotoxicity and weakened immune responses. The transmission of nanomaterials across many trophic levels may exacerbate their toxicity, raising significant issues about ecosystem health and human safety [128].

### Toxicological risks and human health implications of non-biodegradable nanomaterials

**Fig. 7:**
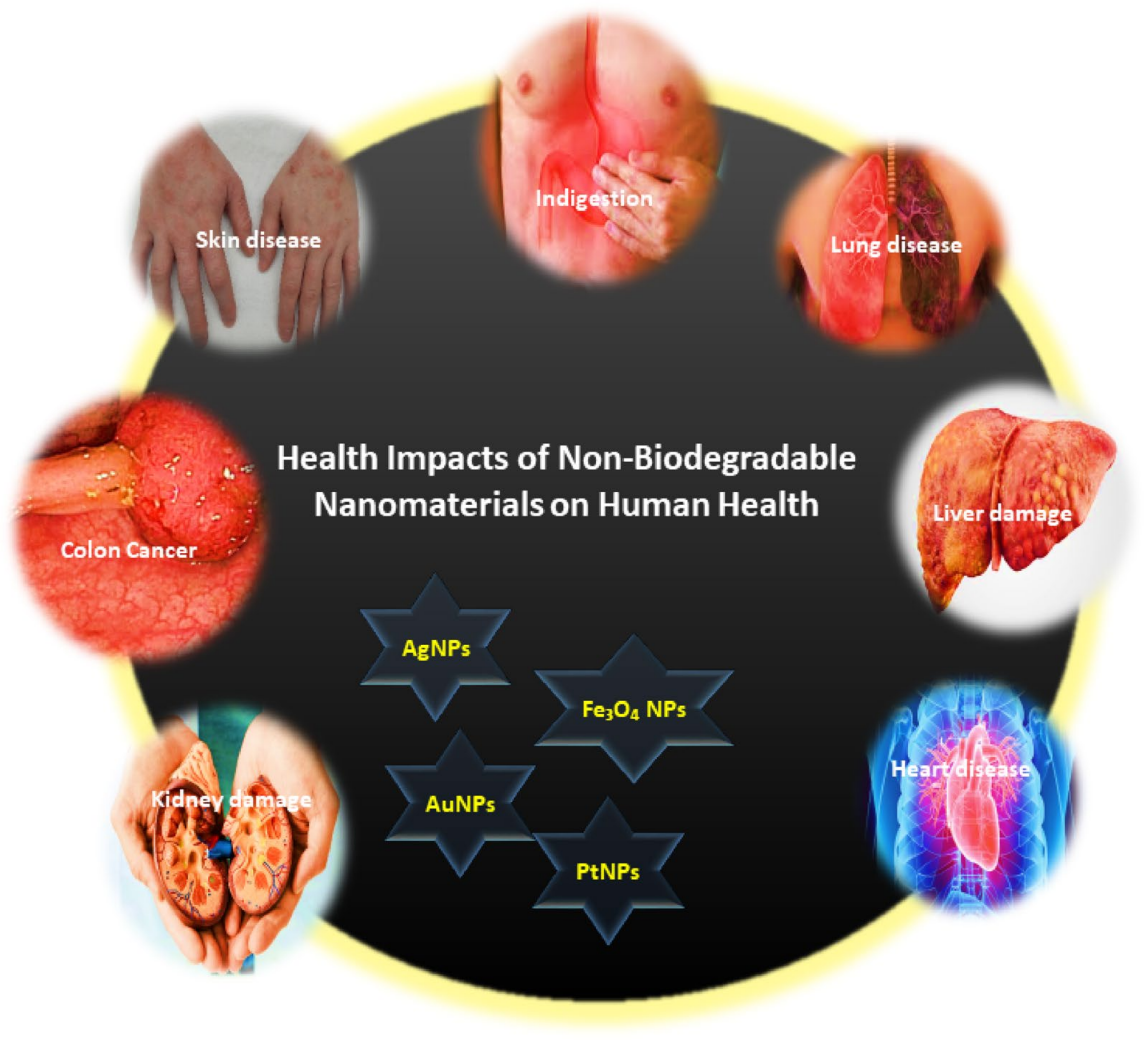
Health impacts of non-biodegradable nanomaterials (AgNPs, AuNPs, Fe₃O₄ NPs, PtNPs) on human health. Exposure to these NPs has been associated with multiple toxicities, including skin disease, indigestion, lung disease, liver damage, heart disease, kidney damage, and colon cancer

Non-biodegradable nanomaterials like AgNPs, AuNPs, PtNPs, and Fe₃O₄ NPs are widely used in industrial, medical, and agricultural settings. However, their persistence in the environment raises serious toxicological concerns, such as lung toxicity (inflammation, fibrosis, and respiratory dysfunction), skin toxicity (allergic reactions and dermatitis), liver damage (oxidative stress and lipid peroxidation), kidney toxicity (nephrotoxicity and filtration interference), and cardiovascular problems (endothelial dysfunction and arterial plaque formation). As shown in Fig. 7. These NPs can cause harm through mechanisms such as oxidative stress, bioaccumulation, and DNA damage, ultimately leading to chronic illnesses and cancer. Prolonged exposure by inhalation, ingestion, or skin contact heightens these dangers. To lessen toxicity, mitigation techniques may include stronger restrictions, improved waste management, and biodegradable alternatives [118, 129].

Additionally, studies have demonstrated that bioaccumulated nanomaterials may change the way hormones function at higher trophic levels. According to Verma et al. (2017), zebrafish embryos may be adversely affected by zinc oxide NPs (ZnONPs), leading to aberrant development and a higher mortality rate [124]. Another study found that while AgNPs exposure decreased ovary size and fecundity in Drosophila, it had no effect on the offspring’s fertility or gender ratios. An essential ecological function, the reproductive rhythm, is lost in earthworms exposed to TiO₂ NPs. Furthermore, pollinator lifespans may be shortened by carbon-based NPs, which would impact plant biodiversity. These effects highlight how urgent it is to use green substitutes, such as nanocellulose scaffolds, which break down innocuously and support microbial homeostasis in chronic wound settings, encouraging infection control innovation without endangering ecosystems [124].

### Green innovations

**Fig. 8:**
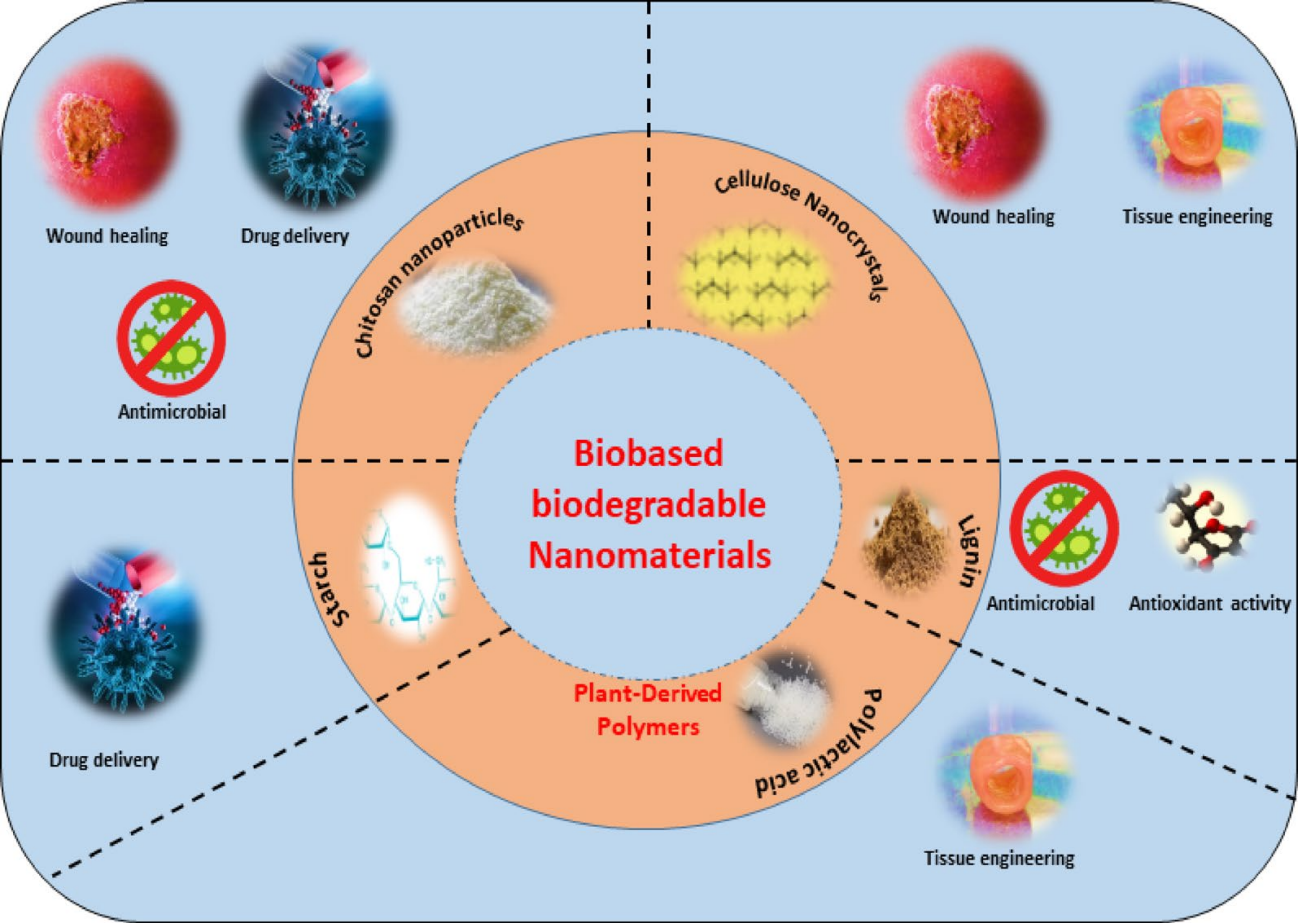
Applications of green biodegradable nanomaterials: This figure shows how different green nanomaterials like chitosan, cellulose nanocrystals, starch, lignin, and polylactic acid are used in medicine. These materials support wound healing, drug delivery, antimicrobial treatment, antioxidant protection, and tissue engineering, making them valuable for safe and sustainable healthcare solutions

To mitigate the environmental and health challenges posed by traditional nanomaterials, attention is shifting toward green nanomaterials derived from sustainable sources. These materials including chitosan, cellulose nanocrystals (CNCs), and plant-derived polymers offer intrinsic biocompatibility, lower toxicity, and often stimulate host tissue regeneration [130]. Figure 8 shows the various applications of green biodegradable nanomaterials.

#### Chitosan NPs for infection control

Chitosan, composed of β-(1–4) N-acetyl glucosamine and D-glucosamine repeating units, possesses native amine groups that are protonated at physiological pH. This cationic nature enables strong mucoadhesive interactions and contributes to its potent antimicrobial activity. Chitosan-based NPs can adhere to mucus membranes, facilitating prolonged and localized drug release, and are widely utilized in wound healing, ocular therapy, periodontal and cardiac repair, and orthopedic tissue regeneration [131]. Its biocompatible and biodegradable properties have led to its application in wound healing, ocular, periodontal, cardiac, bone, and cartilage tissue regeneration. Multiple delivery routes including oral, transdermal, vaginal, ocular, and nasal have been explored to optimize the therapeutic use of chitosan NPs. The antibacterial mechanism of chitosan involves electrostatic interaction with negatively charged bacterial membranes, leading to disruption of membrane integrity and bacterial cell death. Chitosan NPs exhibit strong efficacy against both Gram-positive and Gram-negative strains. AgNPs chitosan composites further enhance antibacterial potency, showing significant inhibitory effects against *E. coli* and *S. aureus* [132, 133]. In regenerative medicine, injectable chitosan-based hydrogels have supported bone tissue formation. A study by Li et al. (2021) demonstrated excellent in vivo regeneration when synovial mesenchymal stem cells (MSCs) were delivered via hybrid chitosan–polycaprolactone (PCL) scaffolds [134, 135].

#### Cellulose nanocrystals in tissue regeneration

Cellulose nanocrystals (CNCs), rod-shaped NPs derived from plant fibers such as wood pulp, bamboo, and cotton, are characterized by high stiffness, mechanical strength, and surface functionality [136, 137]. Their abundance of hydroxyl groups enables scaffold formation and facilitates cell adhesion via electrostatic and hydrogen bonding interactions. CNCs have been effectively incorporated into hydrogel matrices to support cellular differentiation and proliferation, functioning as excellent ECM mimetics [138]. In tissue engineering, CNC-based constructs have demonstrated non-toxic behavior and strong regenerative potential in wound healing, cartilage repair, and osteogenesis [139]. In vivo murine studies further support their use, revealing superior tissue integration and structural support during regeneration [140].

#### Plant-derived polymers for sustainable nanomaterials

Plant-derived polymers offer an environmentally friendly and non-toxic approach to nanomaterial synthesis. Among the most studied are starch, lignin, and polylactic acid (PLA), which have been applied in tissue scaffolds, drug delivery systems, and biomedical devices [141]. Starch is a hydrophilic polysaccharide composed of amylose and amylopectin, offering biocompatibility and cost-effectiveness. Starch-based hydrogels, when cross-linked with other polymers, show structural stability and environmental responsiveness. In one example, Saracoglu et al. (2023) developed starch-based nanogels cross-linked with PEGDE to deliver vancomycin effectively against oral mucositis pathogens [142]. Electrospun scaffolds combining starch and poly(3-hydroxybutyrate) (PHB) exhibit enhanced tensile strength and biodegradability, supporting MG-63 cell viability and bone regeneration [143]. These scaffolds demonstrated up to 15.5 MPa tensile strength and were bead-free, indicating quality fiber formation. Lignin, a renewable polyphenolic polymer, has gained attention for its antioxidant and antibacterial effects. Lignin-based nanomaterials including hydrogels and 3D bioprinting scaffolds have shown low cytotoxicity, high mechanical stability, and wide applicability in tissue engineering [144]. Lanka et al. (2018) showed that glycinated kraft lignin hydrogels cross-linked with carbodiimide achieved favorable mechanical and biocompatibility profiles. PLA, a thermoplastic polymer synthesized from corn and vegetable waste, exhibits high rigidity, biodegradability, and human tissue compatibility [145]. PLA-based scaffolds have shown excellent mechanical properties for musculoskeletal applications, offering tunable degradation rates suitable for regenerative strategies [146].

### Sustainable healing paradigm

The concept of *sustainable healing* integrates effective clinical outcomes with environmental stewardship. It addresses the negative ecological footprint of modern medicine including hazardous waste, pharmaceutical runoff, and high energy and water usage and promotes therapies that reduce environmental impact while supporting patient well-being [147, 148]. Green nanomaterials lie at the heart of this transition. Derived from natural polymers like chitosan, lignin, and cellulose, these materials are engineered to be biodegradable, non-toxic, and biocompatible. For example, chitosan-derived NPs (CDNPs), fabricated through ion gel and solvent evaporation techniques, have demonstrated high cytotoxicity against breast, lung, and colon cancer cells, with minimal impact on healthy cells. Computational modeling further validated their favorable pharmacokinetics and delivery properties [149]. Green-synthesized ZnONPs are sustainable due to their green production using plant or microbial reducing agents, which eliminates the need for toxic chemicals. These NPs demonstrated superior antibiofilm activity and wound healing efficacy compared to conventional antibiotics, while also reducing environmental toxicity. NPs When tested against MRSA-ATCC430, ZnONPs at 65.5 µg/mL inhibited ~ 51% bacterial adhesion and outperformed vancomycin in antibiofilm assays. Cellulose nanofiber hydrogels, applied in porcine punch-biopsy models, achieved ~ 70% wound closure within 14 days significantly better than conventional carboxymethyl cellulose dressings highlighting their regenerative superiority [150]. These nanomaterials are typically synthesized using plant or microbial reducing agents, avoiding toxic chemicals like sodium borohydride or hydrazine that are used in traditional methods [130, 151]. This green synthesis pathway reduces production energy requirements and toxic byproducts. Beyond material innovation, sustainable healing also encompasses institutional practices reducing hospital plastic waste, optimizing energy and water usage, and adopting telemedicine to cut carbon emissions. By prioritizing these smart green nanomaterials, we can achieve effective infection control in chronic wounds while restoring microbial balance and accelerating healing, paving the way for environmentally responsible therapies. By aligning therapeutic design with ecological principles, sustainable healing represents a transformative paradigm that prioritizes both planetary health and medical efficacy. The integration of green nanomaterials into clinical practice has the potential to redefine infection management and regenerative therapy for a sustainable future. To provide a consolidated overview, Table 3 compares the antimicrobial, biofilm-inhibitory, cytocompatibility, and wound-healing features of nanomaterials and hybrid systems.

**Table 3:**
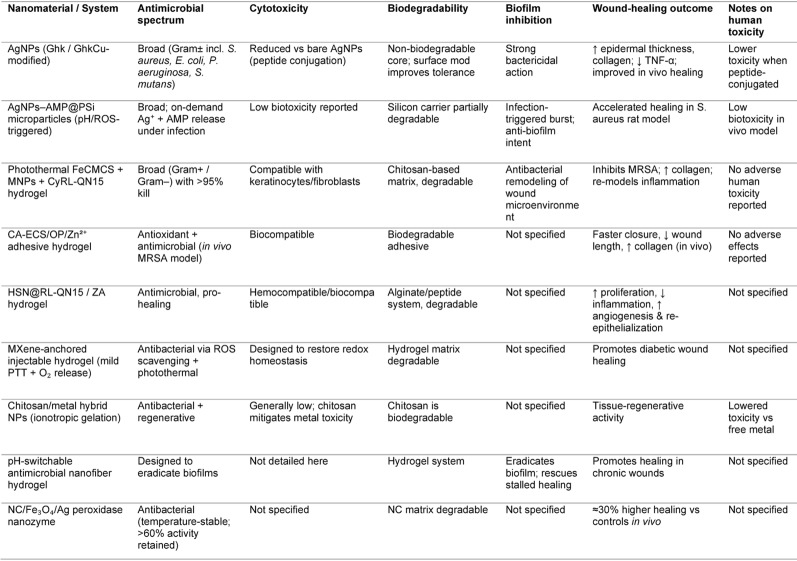
Comparative properties of nanomaterials/systems for chronic infection and wound healing

## Dynamic hybrid nanomaterials for dual antimicrobial and regenerative therapy

**Fig. 9:**
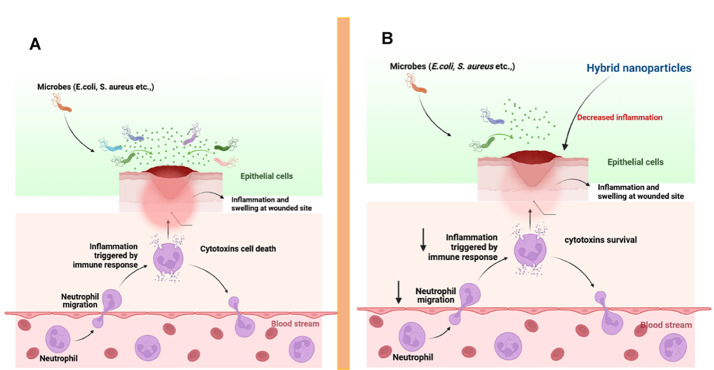
Schematic representation of chronic wound infection and hybrid nanoparticle intervention. **A**: microbial pathogens (e.g., *E. coli*,* S. aureus*) initiate inflammation, neutrophil migration, and cytotoxin release, worsening tissue injury. **B**: hybrid NPs reduce inflammation, promote epithelial repair, and enhance bacterial clearance, collectively supporting wound healing

Although NPs offer promising alternatives, the risk of “nanoresistance” and toxicity from inorganic NPs used alone has highlighted the need for safer and smarter drug delivery systems. Hybrid nanomaterials, typically formed by integrating inorganic NPs (e.g., AgNPs, ZnO) with natural polymers (e.g., chitosan, gelatin, cellulose), are specifically designed to retain the strong antibacterial efficacy of metals while reducing their toxicity through controlled release, improved stability, and enhanced biocompatibility. Engineering hybrid nanomaterials that respond to local physiological cues such as pH, temperature, enzymatic activity, or oxidative stress can significantly enhance the precision and efficacy of antibacterial therapies. In chronic wounds, the pathological microenvironment typically presents with acidic pH, elevated enzymes, ROS overproduction, and bacterial biofilms that hinder healing. Smart nanoplatforms can be programmed to release their therapeutic payloads in response to these cues, ensuring localized activity and minimal systemic toxicity. Figure 9 illustrates how hybrid NPs intervene in this cycle dampening immune-driven inflammation while supporting epithelial repair and reducing bacterial survival at the wound site. This dual antimicrobial and regenerative action underscores their therapeutic superiority for chronic wound management [152]. The following subsections outline key smart material strategies and functional outcomes, highlighting how hybrid nanomaterials through pH-responsive systems, peptide or polysaccharide conjugation, multifunctional hydrogels, antibiotic resistance modulation, and enzyme-based nanozymes translate these dual actions into clinically relevant wound-healing solutions.

### Smart material mechanisms and functional outcomes

#### pH-responsive hydrogels for targeted drug release

To address chronic infections and drug-resistant pathogens, stimulus-responsive smart hybrid nanomaterials have emerged as promising therapeutic systems. These materials overcome limitations associated with traditional nanoparticle-based delivery such as weak tissue penetration, incomplete drug release, and off-target effects by responding to disease-specific microenvironmental cues. Infected and wounded tissues often exhibit altered conditions such as lower pH, elevated ROS, higher local temperature, and enzyme overexpression. Smart hydrogels exploit these physiological differences to trigger localized drug release at the site of infection or tissue damage, enhancing therapeutic precision and minimizing damage to healthy tissues. For example, an octapeptide (IKFQFHFD)-based biocompatible hydrogel demonstrated acidic pH-responsive release at pH 5.5, leading to effective biofilm eradication and activation of wound healing via membrane and cell wall disruption mechanisms [153]. Another study developed pH/ROS dual-responsive metformin-released-enhanced self-healing bifunctional polyethylene glycol-co-poly(glycerol sebacic acid)/dihydrocaffeic acid and L-arginine co-grafted chitosan hydrogel to enhance wound healing through decreased inflammation and enhanced angiogenesis in a rat type II diabetic model. This study also confirmed the wound-healing ability of graphene oxide (GO)/metformin (Met) hydrogels in vivo, indicating their wound healing-promoting effect in diabetic wounds [154]. A new strategy was developed to design pH-responsive hydrogel dressing H-A/SA/P-ZIF (HASPZ), which showed antibacterial and wound healing properties but also has dual pH responsiveness to avoid overuse of medication while effectively treating second-degree burns [155]. Chitosan/metal hybrid NPs synthesized via ionotropic gelation exhibited combined antibacterial and tissue regenerative capabilities, suggesting translational promise [156].

#### Peptide- and polysaccharide-conjugated metal NPs for enhanced antimicrobial and healing effects

Similar to other NPs, AgNPs also exhibit their antimicrobial properties but their toxicity limits their applications. The tripeptides glycyl-L-histidyl-L-lysine (Ghk) and copper peptide (GhkCu) are native to human plasma or saliva and promote wound healing and angiogenesis. When these tripeptides conjugated with AgNPs enhanced synergistic antibacterial, anti-inflammatory and wound healing properties with least toxic effects towards four gram positive and negative strains such as *S. aureus*, *S. mutans*, *E. coli*, and *P. aeruginosa* respectively [157]. Conversely, the use of polysaccharides or oligosaccharides from marine algae connected with AgNPs and antimicrobial peptides (AMR), odorranain A (OA) known as AGO-AgNPs-OA exhibited stronger antimicrobial activity than AGO-AgNPs indicating their good biocompatibility and significant wound healing ability [158]. The development of a photothermal antibacterial composite hydrogel composed of Fe²⁺ cross-linked carboxymethyl chitosan (FeCMCS), melanin NPs (MNPs), and the pro-healing peptide CyRL-QN15 has shown excellent efficacy. This system enhanced keratinocyte and fibroblast proliferation and migration, while exhibiting strong antibacterial activity (> 95% kill) against both gram-positive and gram-negative bacteria. In vivo, the hydrogel inhibited MRSA, promoted collagen deposition, and remodeled the inflammatory microenvironment key processes for chronic wound healing [159]. To increase porosity, hemocompatibility, biocompatibility as well as antimicrobial activity, prohealing peptide RL-QN15 was loaded into hollow silica NPs (HSN) and combined with zinc alginate (ZA) to obtain HSN@RL-QN15/ZA hydrogel. The synthesized hydrogel further enhanced skin cell proliferation, keratinocyte scratch repair, reduced inflammation, improved angiogenesis, and re-epithelialization and tissue formation thus enhancing chronic wound healing [160].

#### Multifunctional hydrogels with adhesive, self-healing, and photothermal properties

**Fig. 10:**
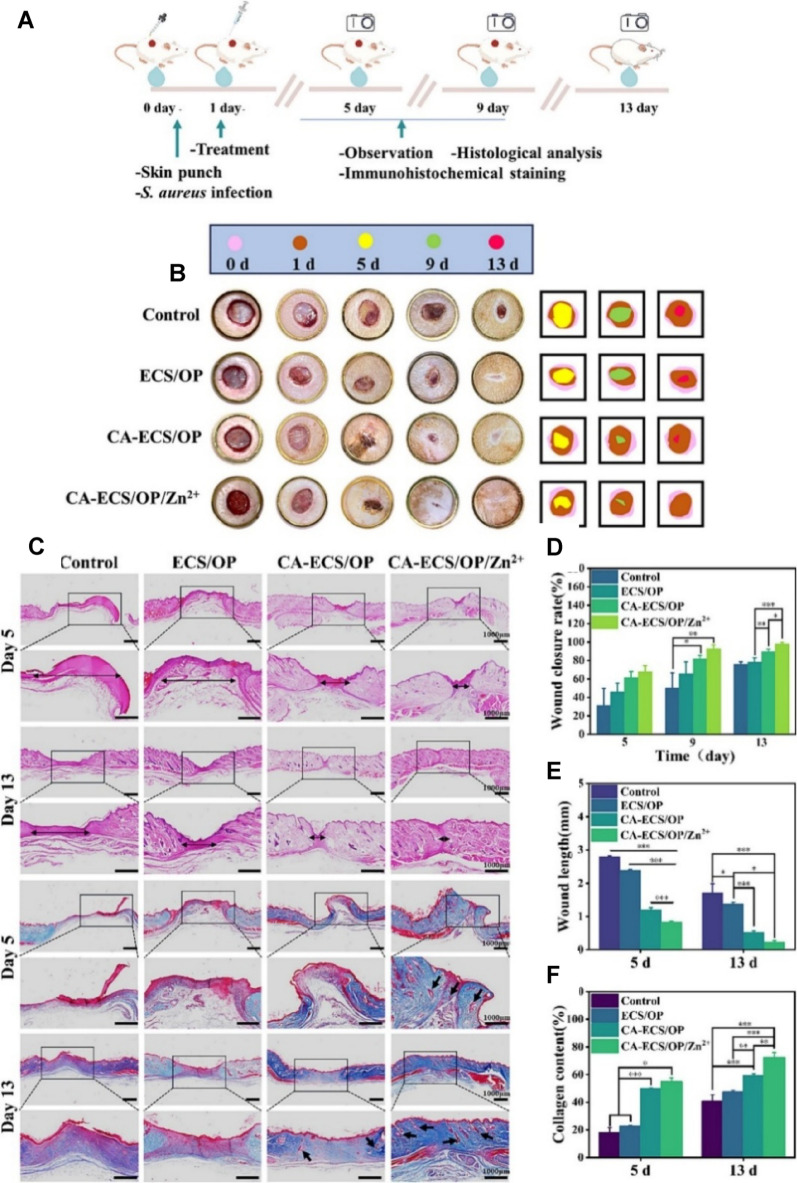
In vivo MRSA-infected wound healing evaluation of the adhesive hydrogel Schematic of the experimental workflow in rat models (**A**), representative wound images and wound closure progression (**B**), histological analysis of skin tissues on Days 5 and 13 (**C**), and quantitative assessment of wound closure rate (**D**), wound length (**E**), and collagen deposition (**F**). The CA-ECS/OP/Zn²⁺ hydrogel demonstrated accelerated wound closure, reduced wound length, and enhanced collagen deposition compared with controls, confirming its superior healing efficacy. This figure adapted from [161]

In another study, a multifunctional wound dressing was developed using a polysaccharide/metal/polyphenol double-crosslinked hydrogel (CA-ECS/OP/Zn²⁺). This platform exhibited strong adhesion, injectability, and enhanced mechanical strength while maintaining biodegradability and biocompatibility. In addition, the hydrogel showed potent antioxidant and antimicrobial properties, collectively supporting its role as a versatile wound-healing material. Its superior performance was validated in vivo against MRSA-infected wounds, where it accelerated closure, reduced wound length, and promoted collagen deposition (Fig. 10).

**Fig. 11:**
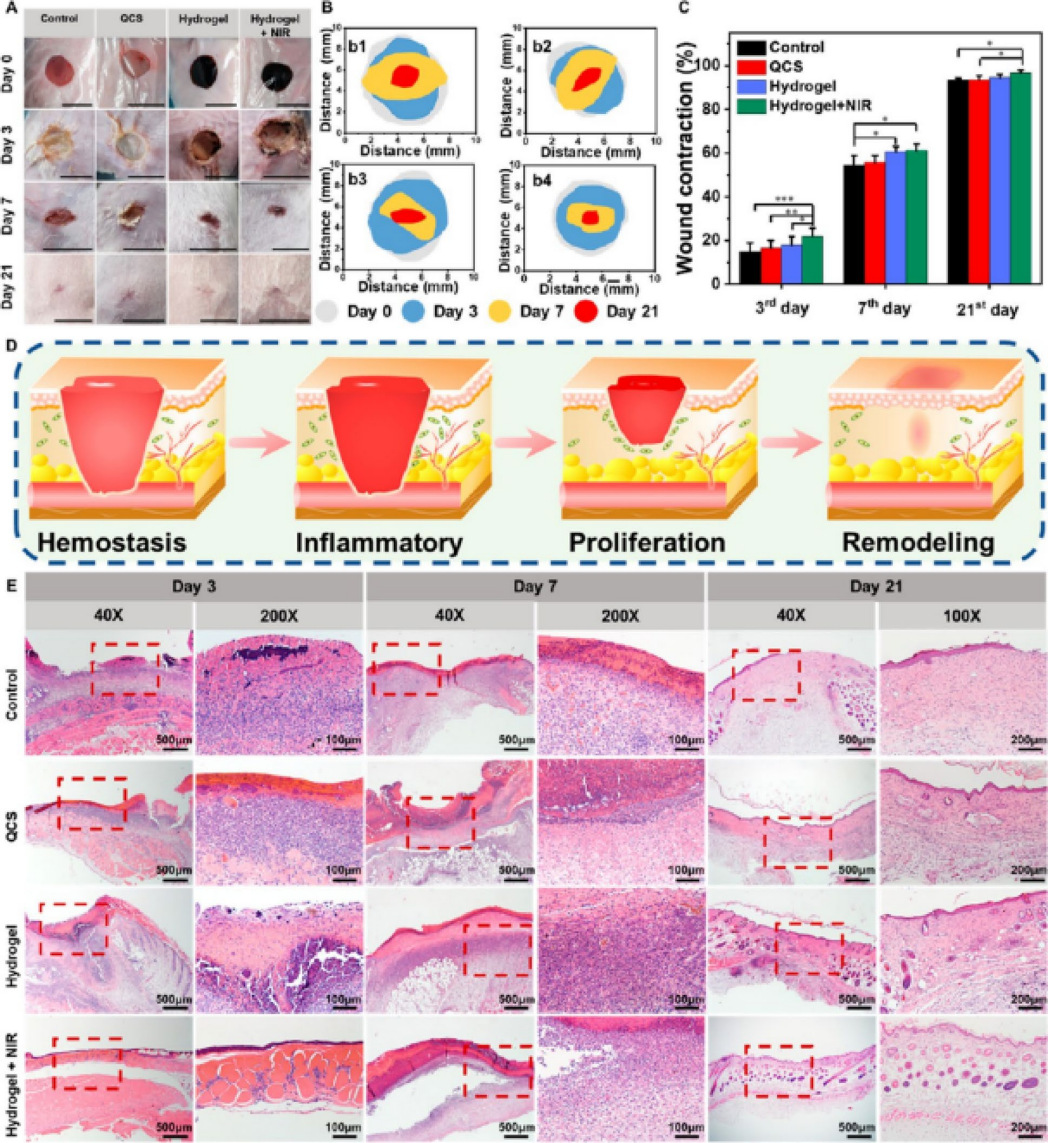
Hydrogel-mediated wound healing in *S. aureus*-infected wounds. (**A**) Representative wound images across different treatments (Control, QCS, Hydrogel, Hydrogel + NIR) on Days 0, 3, 7, and 21. (**B**) Wound size measurements showing progressive contraction. (**C**) Quantitative wound contraction analysis indicating enhanced healing with Hydrogel + NIR treatment. (**D**) Schematic illustration of wound healing phases (hemostasis, inflammatory, proliferation, and remodeling). (**E**) Histological examination at different magnifications confirms superior re-epithelialization, reduced inflammation, and tissue regeneration with Hydrogel + NIR. Adapted from [162–165]

Further. the study also conducted in the wounded rat model showed their significant anti-inflammatory, angiogenic, and folliculogenic properties, thus promoting wound regeneration and healing ability [162]. Considering this fact, a study developed dual dynamic-bond cross-linking between Fe, PA containing aldehyde groups and quaternized chitosan, (QCS) adhesive hydrogels dressing that could repeatedly close the wound to prevent infection and delay healing. Further, the sensitive coordinate bond of catechol-Fe and dynamic Schiff base bonds improved the mechanical properties of the hydrogel as well as their injectability and self-healing properties, thus making the adhesive hydrogel a smart wound sealant to heal infected wounds and improve tissue regeneration in vivo. Additionally, this study also compared the healing effects of QCS-PA@Fe10 hydrogel and QCS-PA@Fe10 hydrogel with NIR irradiation after MRDS-infection along with the wounds treated with pure QCS and Tegaderm dressings [161]. The results showed that compared all other hydrogels treated, hydrogel treated with NIR irradiation showed more contraction of wound indicating better healing efficacy, (Fig. 11).

The self-healing hydrogels can initiate self-repair in the wounding site after application [166, 167]. Therefore, the development of conductive self-healing and adhesive hydrogels based on N-carboxyethyl chitosan (CEC), benzaldehyde-terminated pluronic F127/carbon nanotubes (PF127/CNTs) are used to treat bacterial-infected wound repair [163]. In DFU disease, hypoxia generation causes impaired angiogenesis and prolonged inflammation associated with bacterial infection. Therefore, a study was conducted to develop injectable hydrogel using hyaluronic acid-graft-dopamine (HA-DA) and polydopamine (PDA) coated Ti_3_C_2_ MXene nanosheets that were cross-linked by an oxyhemoglobin/hydrogen (HbO_2_/H_2_O_2_) along with mild photothermal stimulation effect. Hydrogel containing HbO_2_ performs dual functions, i.e., it acts as horseradish peroxidase-like to catalyze hydrogel formation but also carries oxygen and release in a sustainable manner upon near-infra red exposure (NIR). The presence of MXene in the hydrogel is responsible for the continuous oxygen release and to scavenge excessive ROS including H_2_O_2_, O2•–, and •OH, thus aiding in intracellular redox homeostasis to heal infections caused by bacteria. Overall, MXene-based hydrogel promotes tissue adhesion, self-healing ability, injectability, and homeostasis upon mild photothermal effects that stimulate diabetic wound healing [168].

#### Strategies to combat antibiotic resistance using nano-delivery systems

**Fig. 12:**
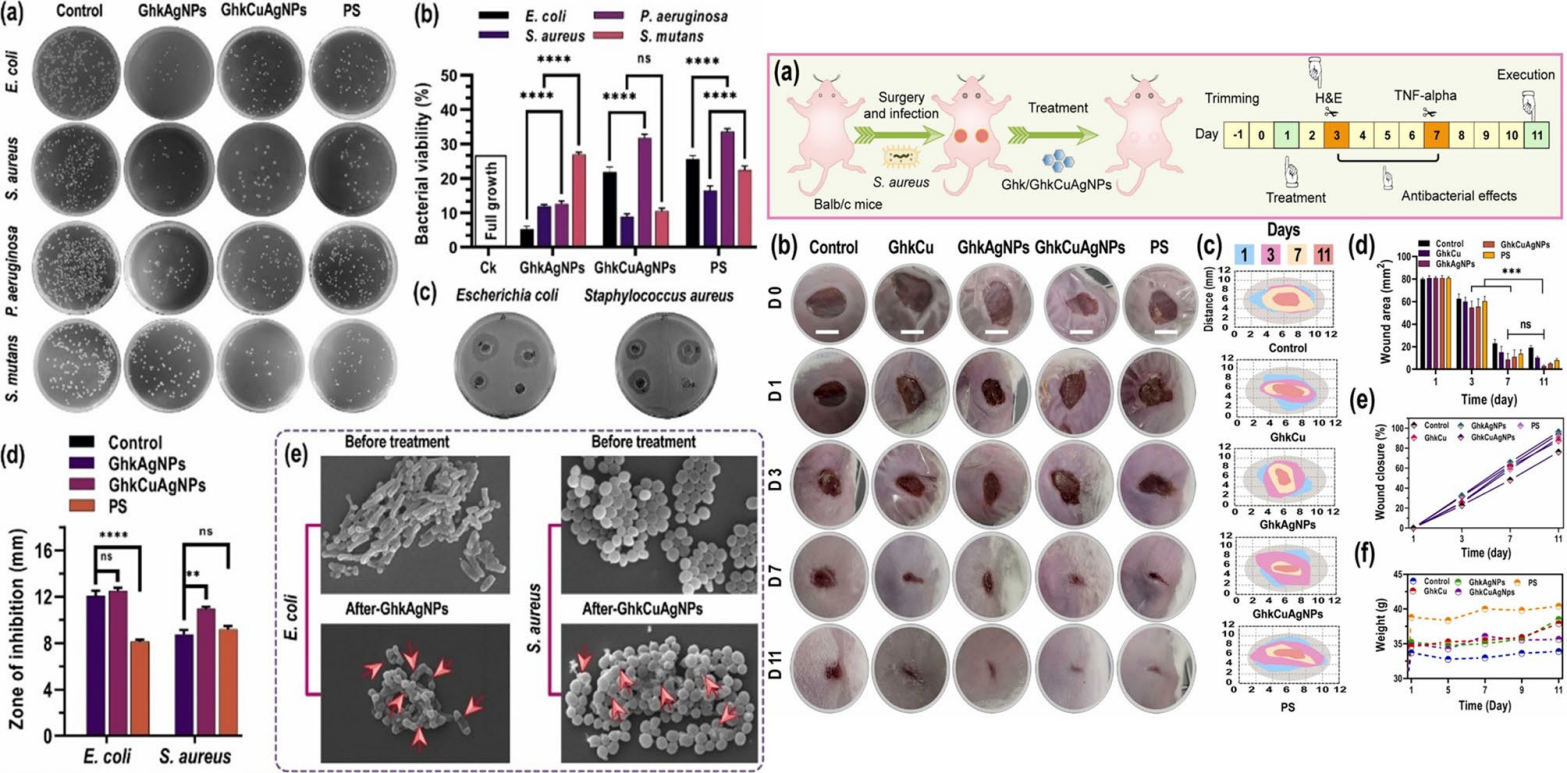
In vitro and in vivo antibacterial evaluation of Ghk- and GhkCu-modified AgNPs. (**A**) In vitro antibacterial activity against *E. coli*,* S. aureus*,* P. aeruginosa*, and *S. mutans*, showing bacterial viability, inhibition zones, and morphological changes under SEM. (**B**) SEM images highlight bacterial membrane disruption after treatment. (**F**) In vivo therapeutic efficacy of GhkAgNPs and GhkCuAgNPs in *S. aureus*–infected wounds in Balb/c mice, including wound healing progression, wound area quantification, histological evaluation, and cytokine analysis. This figure was adapted from [157]

Bacterial multidrug efflux pumps are the major contributors to antibiotic resistance formation in microbial pathogens. Therefore, discovering the nanosystems inhibiting multidrug efflux can maximize the antibiotic effect and promote efficient healing of chronic wounds. Therefore, a study used dual molecules such as ciprofloxacin (CIP) and an efflux inhibitor 5-Nitro-2-(3-phenylpropoxy) pyridine (5-NPPP) were used to synthesize and deliver together with an antibiotic can re-sensitize pathogen against antibiotics and simultaneously treat the infections. Similarly, to improve sustained release of the drug and to re-sensitize fluroquinolone-resistant strain, CIP was loaded into PLGA microspheres in combination with ginsenoside Rh2 to treat *S. aureus*-induced skin infections [169]. Another study reported the use of CIP loaded hydrogels decreased the wound contraction and promote epithelialization without affecting the granulation tissue deposition and remodeling during the healing process [170]. In another work, the combination of cyclodextrin (CDs) with agar to render chemically cross-linked hydrogels with drugs by the formation of inclusion complexes or ionic bonds with polysaccharide enhanced loading ability, dually controlled delivery, release profile based on CD affinity and tunable external stimuli such as pH and ionic strength [171]. An interesting study used peptides such as Ghk, GhkCu-modified AgNPs that promoted cell migration and proliferation in dermal fibroblasts in wounding sites by exhibiting anti-bacterial effects against *S. aureus* and *E. coli* (Fig. 12). These tripeptides-modified AgNPs also enhanced tissue regeneration, increased epidermal thickness, collagen deposition, and reduced TNF-alpha expression to promote wound healing mechanism.

#### Synergistic effects of antimicrobial peptides, polysaccharides, and enzymes in hybrid nanomaterials

Recent studies showed a synergistic effect of using antimicrobial peptide and NPs together to enhance the overall antibacterial effect and to overcome conventional antibiotic-related disadvantages. In this line, a study used peptide-conjugated phage-mimicking NPs (PhaNP@Syn71) to improve wound size and enhance infection-free wound healing in vitro and in vivo. The phage-mimicking NPs are considered advantageous because of their evolutionary constant shape, improved antibacterial effect, and lack of immune reactions in the human body [166]. Using ultra-short anti-microbial peptides (USAMPs) has gained structural and economic advantages over AMPs, such as improved mammalian cell toxicity, enhanced potency, and reduced cost. Therefore, a study used WOW peptide conjugated with AgNPs to study the antibacterial effect on various pathogens such as *S. aureus*, *E. coli*, MRSA, and ESBL *E. coli*.

The result showed that AgNPs conjugated with WOW-peptide showed the highest antibacterial effect compared to WOW peptide, thus demonstrating their potential against bacterial infection with the least toxicity [168]. Another study used peptide-AgNPs conjugates using myristoyl tetrapeptide 6 (MT6) or copper tripeptide 1 (CuTP1) to compare wound healing capacity with peptide-free AgNPs. The conjugated peptide (MT6) with AgNPs exhibited 71.97 ± 4.35% wound closure with enhanced wound Healing ability that is 2.82-fold better than CuTP1 respectively [172].

Apart from peptides, polysaccharides also have potential in wound healing and anti-bacterial effect due to their high biocompatibility and physicochemical properties. For instance, a study used agarose polysaccharides/oligosaccharides from marine red algae for AgNPs synthesis and further used odorranain A (OA) to synthesis novel AGO-AgNPs-OA. This study showed AGO-AgNPs-OA had the strongest antibacterial effect and wound healing property compared to AGO-AgNPs respectively [158]. Another interesting used covalently grafted quaternary ammonium groups (QAGs) containing 12-carbon straight-chain alkanes to the dextran polymer skeleton. Further, the product obtained from the above reaction was oxidized into quaternized dextran (OQD), which is high in antibacterial QAGs and aldehyde groups. The resulting QAGs can react with glycol chitosan (GC) via Schiff-base reaction to form GC@OQD hydrogel with significant self-healing behavior hemostasis, injectability, inherent superior antibacterial activity, biocompatibility, and excellent promotion of healing of *MRSA*-infected wounds. Additionally, GC@OQD hydrogel can also treat irregular wounds thus indicating their therapeutic role as wound dressing in clinical practice [173]. In another study, researchers used polysaccharides with polyacrylamide to exhibit cytocompatibility, antibacterial activity, and excellent tissue adhesion. To achieve this, they used alginate/chitosan-based hydrogels and cross-linked with tissue surface to achieve tissue adhesion, whereas the use of polysaccharide further promoted the tissue adhesion, thus resulting in good biocompatibility and enhanced antibacterial activity, which can be used in tissue engineering [174]. Recent study also used naturally derived polysaccharides from *Bletilla striata* polysaccharide and Berberine (BER) with borox via borate ester bond to develop self-healing hydrogels to treat diabetic ulcers (DUs). The developed hydrogel exhibited an anti-bacterial effect of over 90% and wound healing effect against *E. coli* and S. *aureus*. Another interesting study used immune-regulating polysaccharide-based hybrid hydrogel with mild photothermal effects. Specifically, they designed injectable self-healing hydrogel of oxidized konjac glucomannan/arginine-modified chitosan (OKGM/CS-Arg, OC) conjugated with protocatechualdehyde-@Fe (PF) NPs which can absorb near-infrared radiation. When PTT and OC/PF were used in combination, they exhibited antibacterial effects by promoting cell migration and angiogenesis. Additionally, in methicillin-resistance, *S. aureus*-infected full-thickness mouse wounds showed significant antibacterial and anti-inflammatory effects by stimulating wound healing ability and by improving wound immune microenvironment, thus can be confidentially used in managing infectious wound dressings [175].

Several studies also conducted using enzymes for nanomaterials to enhance wound healing and to eradicate harmful pathogenic infection at wounding site. For instance, self-activated cascade nanozyme, f-FeNC@GOx are activated in the presence of glucose can activate highly toxic hydroxyl radicals (▪OH), develops ability to eradicate bacteria and promotes wound healing [176]. Therefore, the f-FeNC@GOx complex can be widely used as an antibacterial drug for eradicating pathogens to promote wound healing. Another study used pH responsive nanozyme hydrogel loaded with glucose-oxidase (GOx), exhibits intrinsic GOx, peroxidase (POD)-, oxidase (OXD)-, catalase (CAT)- and superoxide dismutase (SOD)-like activities with pH-switchable glucose-initiated cascade reaction for diabetic wound Healing. The 1st cascade of reaction generates superoxide anion radical (O_2_^·−^) and hydroxyl radicals (·OH) which could be responsible for eradicating bacteria at the wounding site whereas, 2nd cascade reaction alters the wound pH environment from acidic to alkaline by the decomposition of endogenous and exogeneous H_2_O_2_ unto O_2_ thus eliminates the oxidative stress and hypoxia condition. In addition, the gel was designed with water/alcohol solubility to prevent secondary injury to the wounding site. Overall, the synthesized multifunctional hydrogel efficiently promoted pro-angiogenesis and enhanced bacterial-infected wound healing ability [177]. Alternatively, studies were also conducted using *Eragrostis teff* straw based nanocellulose (NC) to synthesis NC/Fe_3_O_4_/Ag peroxidase nanozyme as an antibacterial and wound healing agent. The synthesized nanozyme was found to be temperature stable while Maintaining over 60% of its anti-bacterial activity in vitro. Also, in vivo studies demonstrated the 30% increased wound healing ability of nanozyme compared to control-based ointment and commercial nitrofurazone ointment, respectively [178].

#### Stimuli-responsive platforms for on-demand drug release and microenvironment remodeling

Generally, the release dynamic curve of a drug delivery system is static and unchanged upon stimuli thus causing failure of administered drug to be released upon demand and therefore discovering the drug carriers that contain fine tunable properties including tunable pore structure, surface chemistry, bioavailability, and degradability, is extremely considered advantageous when considering improving the site-specific release of drugs. Such mechanisms enable precise control over therapeutic delivery, where, for instance, pH-sensitive linkages break down in acidic environments to release payloads directly at infection sites, or ROS-triggered oxidation facilitates burst release of antimicrobials. Recently, the dual synergistic antibacterial platform was developed using AgNPs and AMP coloaded porous silicon (Psi) to execute on-demand release ability. This interesting combination of AgNPs and AMP coloaded porous silicon microparticles (AgNPs-AMP@PSiMPs) possess acidic pH and ROS-stimulated release of Ag + ions and AMPs under bacterial infection due to their oxidation and desorption effects. This combination also exhibited excellent bacterial-killing effect, wound-healing, and low biotoxicity in a *S. aureus*-infected rat wound model [171]. The remodeling of the microenvironment has been achieved through the use of a bacteria-responsive self-activating hydrogel platform with increased anti-bacterial properties in the infection stage to promote wound closure, accelerate wound collagen deposition and promote wound closure; thus the engineered hydrogel is named as “self-diagnostic” that can be used to alter wound microenvironment to promote wound healing in an intelligent manner [179]. These functional outcomes stem from intricate material designs, such as dynamic cross-linking bonds that allow self-healing or photothermal conversion for targeted bacterial ablation, ultimately translating molecular-level responses into macroscopic tissue repair.

### Broader advantages of intelligent hybrid nanomaterials in clinicals and systemic context

**Fig. 13:**
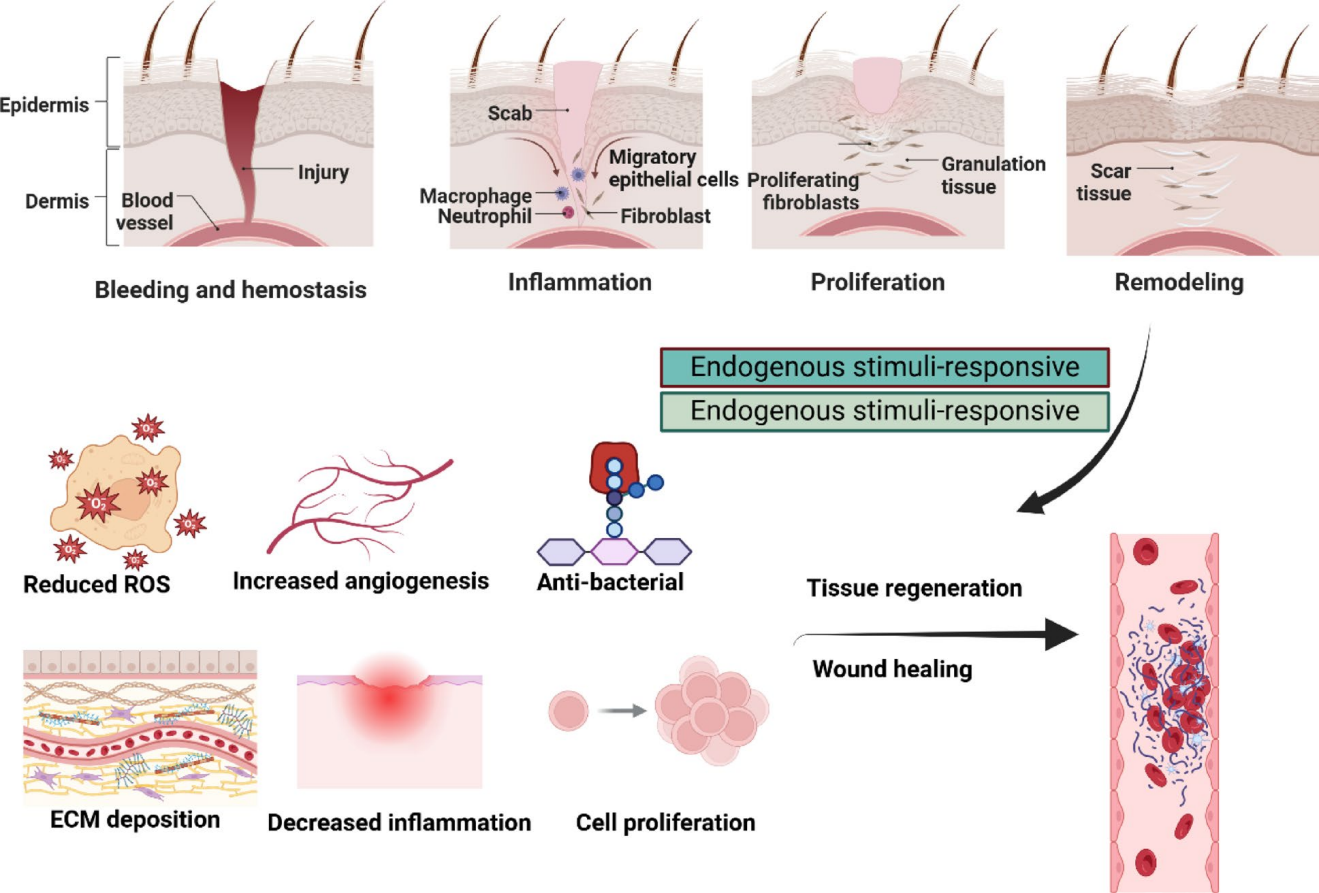
Benefits of smart hybrid nanosystems in regenerative medicine: This figure illustrates how smart hybrid nanomaterials support each stage of wound healing from hemostasis and inflammation to proliferation and tissue remodeling. These systems respond to internal biological cues to promote antibacterial action, ROS, enhance angiogenesis, modulate inflammation, and boost ECM deposition and cell proliferation. Collectively, these effects accelerate tissue regeneration and improve wound healing outcomes

In recent years, addressing chronic wounds, including diabetic wounds, injuries, burns, ulcers, etc., has become a serious threat among health professionals due to the poor quality of patient’s life and the burden on the healthcare system. There are various methods employed traditionally to address various wounds. However, wound dressings are mostly used as a physical barrier to prevent external infections rather than focusing on treatment or tissue regeneration. Slowly, slightly advanced techniques containing antibiotics and antiseptics were developed and commonly used in wound dressings including tetracyclines, quinolones, aminoglycosides, and cephalosporins (Fig. 13).

**Fig. 14:**
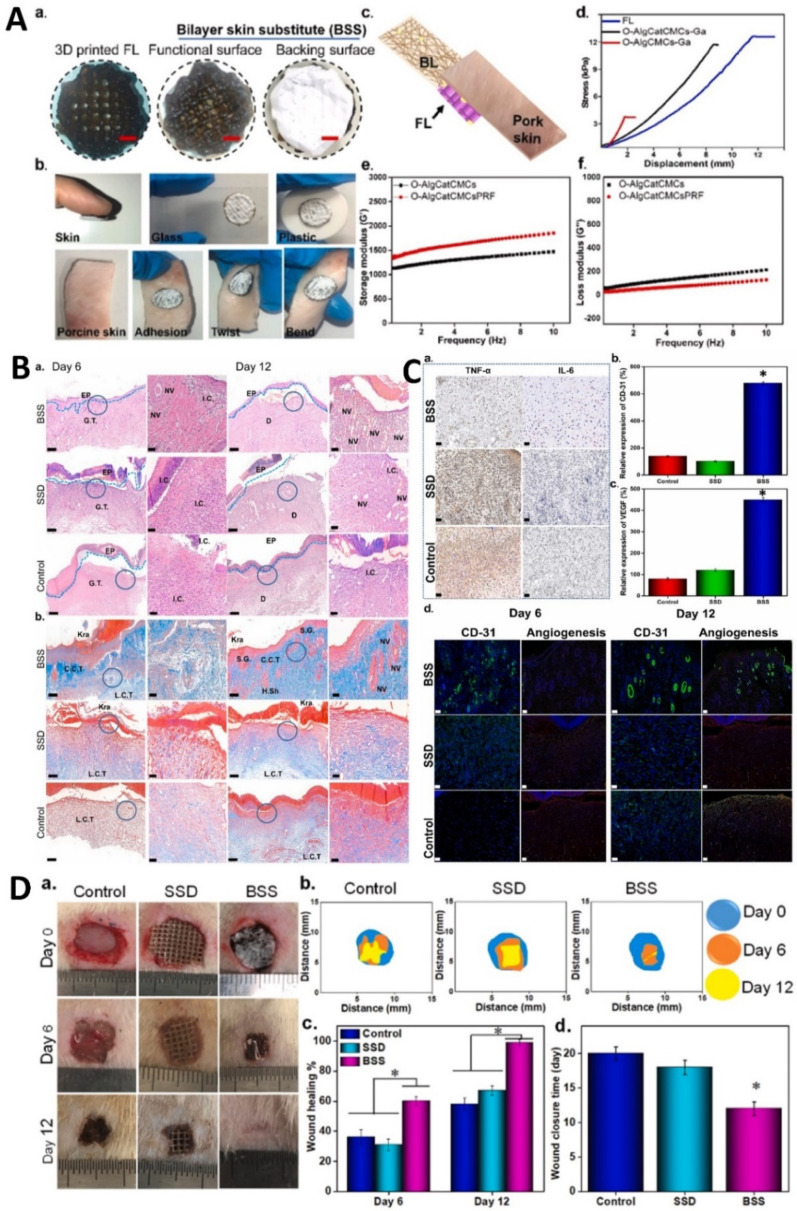
(**A**) The visual appearance and shear strength of BSS. (**B**) Histological analysis of regenerative skin tissue, stained with (**a**) H&E and (**b**) Masson at day 6 (**C**) (**A**) Immunostaining of TNF-α and IL-6 on day 6 (scale bare = 100 μm) (**D**) In vivo infected burned wound healing studies of the BSS, commercial SSD and control (without treatment). This figure is adapted from [184]

This method of dressing can target protein and nucleic acid synthesis of the bacteria and cause metabolic disturbances and bacterial cell wall integrity. Although this method gained some advantages, the improper dosage/use of antibiotics causes further burden, leading to antibiotic resistance than treatment itself. It is also noteworthy to emphasize that more than 70% of colonized bacteria in wound microenvironments are known to exhibit antibiotic resistance [180]. Therefore, conventional approaches fail to address chronic wounds effectively, thus resulting in endless treatment schedules. To overcome these burdens, modern technology attempted to replace conventional treatments by designing smart hybrid nanosystems that can deliver drugs to localized sites to prevent infection by overcoming barriers imposed by biofilms and regulating complex wound microenvironment, thus promoting tissue regeneration in the wounded site. Such intelligent NPs can be engineered and incorporated into the dressing to respond to infected microenvironments such as acidic pH, specific enzymes, and bacterial toxins, as well as to external stimuli, light, thermal, etc., to promote wound healing [181]. These systems offer superior precision over traditional methods by enabling on-demand responses, reducing side effects, and improving patient compliance through minimally invasive applications. Moreover, they mitigate antibiotic resistance by minimizing drug usage and targeting pathogens selectively, potentially lowering healthcare costs by shortening treatment durations and reducing hospital stays. In a study, they developed easy-to-use, innovate regenerative product, “placental multipotent mesenchymal stromal cell” (MMSC) secretome-based chitosan hydrogel (MSC-Ch-gel) effectively promotes infected wound healing in rats with third-degree burns. Specifically, the application of MSC-Ch-gel effectively addressed microorganisms associated wounding by minimizing inflammation, enhanced re-epithelialization, and stimulated well-vascularized granulation tissue due to the pleiotropic and multi-targeted effects of stem cell secretome thus decreasing the application of antibacterial and antiseptic drugs. Although the use of these systems might control the spread of microbial pathogens, the effects observed in this study are not optimal; future studies need to be conducted to improve the application of MMSC secretome-based products to treat chronic injuries and wounds [182]. Thermoresponsive gels containing AuNPs were synthesized using Pluronic^®^127 alone (F1) and with hydroxypropyl methylcellulose (F2) and are considered smart intelligent nanosystems as they perform dual-action such as antibacterial and wound healing effect. The use of this smart system improved bioavailability, skin permeation, and antibacterial and anti-inflammatory activity in vitro, in vivo, and ex vivo conditions. They also didn’t exhibit any kind of skin irritation or sensitivity in guinea pigs, and therefore, this can be used as a smart, topical antibacterial drug delivery system to treat skin inflammation and wound healing [183]. A 3D printed hydrogel scaffold was designed to form bilayer skin substitute (BSS) that prevent fluid entry into the wounding site and so controls bacterial infection induced chronic wounds (Fig. 14).

**Fig. 15:**
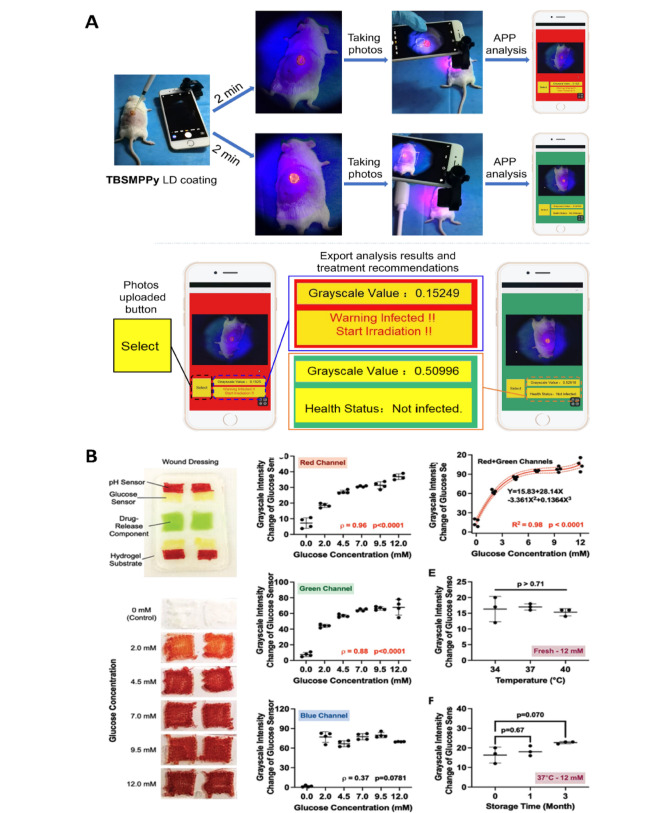
Smart wound monitoring and responsive hydrogel systems. (**A**) In vivo monitoring of fungal-infected wounds using a smartphone-based imaging and analysis platform. Real-time photos are processed by the app to calculate grayscale values, providing infection alerts and treatment recommendations [185]. (**B**) GelDerm hydrogel platform integrating pH and glucose sensors with drug-release components. Panels show (i) wound dressing design, (ii) colorimetric changes with increasing glucose concentrations, (iii–iv) grayscale intensity calibration across RGB channels, and (v–vi) stability of glucose detection under varying temperatures and storage conditions [186]

The 3D printed functional layer (FL) contains catechol, gallium, and biologically active platelet-rich fibrin (PRF) that promotes both tissue and backing layer adhesion to enhance antibacterial effects, angiogenesis, cell growth, and migration thus can be therapeutically used to treat burned wounds [184]. Another interesting study developed on demand photodynamic therapy “AIEgen-based smart system” for monitoring fungal-infected wound monitoring on the mobile phone in sensitive to pH (Fig. 15A). This strategy allowed the patient to monitor the wound and, if necessary, to provide photodynamic therapy by switching on the white light. This also, in turn, allows doctors to evaluate the wound healing process and provide treatment plans according to software results, thus resulting in significant improvement in medical efficacy by providing intelligent scientific technical support [185]. This method of photodynamic therapy also overcomes the use of antibiotic resistance drugs and creates a path to develop smart wound dressing and promising preclinical research and diagnostics [185]. For the synergistic action, drug delivery, and sensing (pH and glucose) dual action approach were integrated that could aid continual monitoring of cutaneous wounds (Fig. 15B). The incorporation of growth factor delivery modules and an array of colorimetric glucose sensors into wound dressing stimulates wound healing. This smart dressing allows both infected and non-infected wounds to heal efficiently and helps in real-time monitoring of wounds as well as early detection of wound infection [186]. All things considered, these benefits demonstrate how intelligent hybrid nanomaterials not only outperform conventional dressings in terms of effectiveness but also open the door for customised, responsive treatments that adjust to the unique dynamics of each wound, ultimately lowering medical expenses and enhancing long-term results. These materials change wound care from reactive to proactive by incorporating diagnostic capabilities, like real-time monitoring through smartphones or sensors. This improves patient engagement and makes telemedicine applications possible in environments with limited resources.

### Clinical validation

Even though several earlier studies on different stimuli-responsive hybrid nanomaterials demonstrated encouraging outcomes in both in vitro and in vivo investigations of wound healing and tissue regeneration, issues still need to be resolved before applying them in clinical settings. Some nanomaterials have already progressed into clinical practice or trials. For example, silver-based wound dressings (e.g., Acticoat^®^, Aquacel^®^ Ag) are FDA-approved and widely used for chronic wounds and burns, while chitosan-based bandages and cellulose nanofiber scaffolds are being tested in early-phase trials, showing reduced infection rates and faster re-epithelialization. These examples illustrate that nanomaterials are not only experimental concepts but are also beginning to demonstrate clinical utility.

The challenges faced by the nanomaterials in the process of clinical translation are highly complex due to the fate of nanomaterials in vivo, specifically stability issues as it is exposed to various biological systems, safety/toxicity issues due to bio-accumulation of non-degradable materials, and so on. For Successful and sustainable wound dressing, Materials must be biocompatible, prevent skin allergies and skin irritations, be capable of renal clearance, etc. Also, most of the studies using wound dressing were specific to the study, and not one wound dressing fit for all types of wounds, Making the treatment plan complicated. Apart from this, depending on the types of wound dressing, safety profiles can vary. For instance, 3D structure-based nano-in-microparticles synthesized using polysaccharides such as maltodextrin or dextran, amino acids, and doped with copper oxide (CuO) or zinc oxide (ZnO) NPs are used for skin repair and to prevent bacterial infection by overcoming colonization. This safety profile using nano-in-microparticles also indicated that nano-in-microparticles can degrade completely within 10 days, enhance tissue repair by promoting adhesion and proliferation. Additionally, no sign of inflammation is presented indicating that nano-in-microparticles don’t stimulate immunological response. However, it is known that CuO-NPs can exhibit toxicity in rats when administrated orally into 1000 mg/kg for 28 days and result in ROS accumulation. Similarly, 250 mg/kg ZnO NPs administrated for 7 weeks accumulated Zn in the kidneys and Liver of mice. Nevertheless, metal oxide doses administrated at 1mg/kg in a single dose need proper safety profile investigation for use in clinical settings [187]. When nanoparticle-hydrogel used alone or in combination safety issues must be properly investigated. Compared to the single nanoparticle used, the combined nanoparticle-hydrogel possesses a high risk in terms of safety, and therefore, it must be systematically investigated. For this, in vivo interaction of the system, and their level of toxicity and fate of the nanosystems inside the body must be thoroughly investigated [188].

Similarly, the use of enzyme-based hydrogels is extensively investigated in vitro; their interactions inside the human body are yet in the budding stage. Also, when applied to clinical practice, it is essential to use a simple method to synthesize nanozyme-hydrogels. When the above concerns are addressed along with the safety and efficacy of nanozyme-hydrogels, they can be freely used in patients to treat chronic wounds in real-world applications [189]. A near-infrared-responsive analgesic black phosphorus-based gel effectively relieves pain, heals chronic and harsh wounds, promotes angiogenesis, and reduces inflammation, suggesting their promising role in diabetic wound ulcer treatment. The study also emphasized the gel can also be used in treating other chronic wounds as well as inflammation. Despite their advantageous nature, black phosphorous NPs biosafety must be assessed in human subjects to used them confidently in clinical settings [190]. The self-healing ability and pH/ROS responsiveness of multifunctional glycopeptide EPMA hydrogel as a wound dressing enhanced skin tissue regeneration and wound repair with multifunctional biological activities, including anti-inflammatory, anti-bacterial, and angiogenesis activation as well as macrophage polarization regulation. This innovative smart hydrogel is an ideal candidate for clinical application and translational research [191]. Despite these promising advances, clinical validation remains limited by small cohort sizes, short follow-up durations, and inconsistent trial designs. Regulatory pathways also remain unclear, with some nanomaterials classified as medical devices and others as drugs, slowing down translation. Addressing these hurdles will be crucial to move multifunctional nanomaterials from promising laboratory prototypes into standardized therapies (Table 4).

**Table 4:** Provides a clinical-translation snapshot of representative nanomaterial platforms for wound healing, highlighting their current clinical status, therapeutic applications, benefits, and limitations

Nanomaterial/System	Clinical status/Stage	Key application/Use case	Reported benefits & findings	Limitations & challenges
AgNPs dressings (e.g., Aquacel^®^ Ag+, Acticoat^®^)	FDA-approved, widely used for chronic wounds	Chronic wounds, burns	Broad-spectrum antimicrobial, effective biofilm disruption, cost-effective and patient-comfort advantages	Potential cytotoxicity at high doses, emerging resistance risks
Chitosan-based dressings/nanofibers	Early-stage clinical trials and translational design	Hemostasis and infection control in wounds	Promising antimicrobial, hemostatic, and healing properties; stimuli-responsive and immunomodulatory systems in development	Limited large-scale clinical data; manufacturing consistency issues
Cellulose nanofiber scaffolds (e.g., TEMPO-oxidized)	Pilot clinical/large animal validation	Wound repair, hemostasis	Enhanced epithelialization and hemostatic efficacy, demonstrated safety in human dermal cells and large animal models	Cost and scale-up challenges
Chitosan/cellulose composite hydrogels	Preclinical studies	Chronic and drug-resistant wound healing	Excellent antibacterial activity (> 99.9%), biofilm inhibition, and tissue management capability	Preclinical stage only; clinical translation pending
Hydrogel nanogels (e.g., Ag-loaded, immunomodulatory)	Preclinical, in vivo validation	Antimicrobial delivery, immune stimulation	Shows accelerated wound healing via encapsulated antibiotics or immunomodulators	Needs systematic safety and long-term effect evaluation

To accelerate clinical translation, a stepwise pathway needs to be established. First, reproducible GMP-compliant large-scale manufacturing of nanomaterials and hydrogels is essential to ensure batch-to-batch consistency. Second, standardized safety evaluation frameworks including long-term biodistribution, immunogenicity, and clearance studies should be aligned with FDA/EMA guidelines to reduce regulatory uncertainty. Third, adaptive clinical trial designs involving larger patient cohorts and multi-center validation will be crucial to demonstrate efficacy beyond preclinical promise. Finally, integration into existing wound-care protocols through collaborations with clinicians and industry partners can facilitate adoption. Together, these steps outline a feasible bench-to-bedside strategy, addressing both regulatory and translational gaps. Overall, smart, stimuli-responsive nanomaterials are steadily moving from preclinical innovation toward clinical adoption. With their ability to combine antimicrobial, anti-inflammatory, and regenerative functions, they represent a promising alternative to conventional wound dressings. However, their widespread clinical use will depend on rigorous safety evaluation, standardized manufacturing, and clearer regulatory pathways. If these challenges are addressed, multifunctional nanomaterials could become a frontline option for managing chronic wounds and inflammation.

## Toxicology and environmental impacts of hybrid nanomaterials

**Fig. 16:**
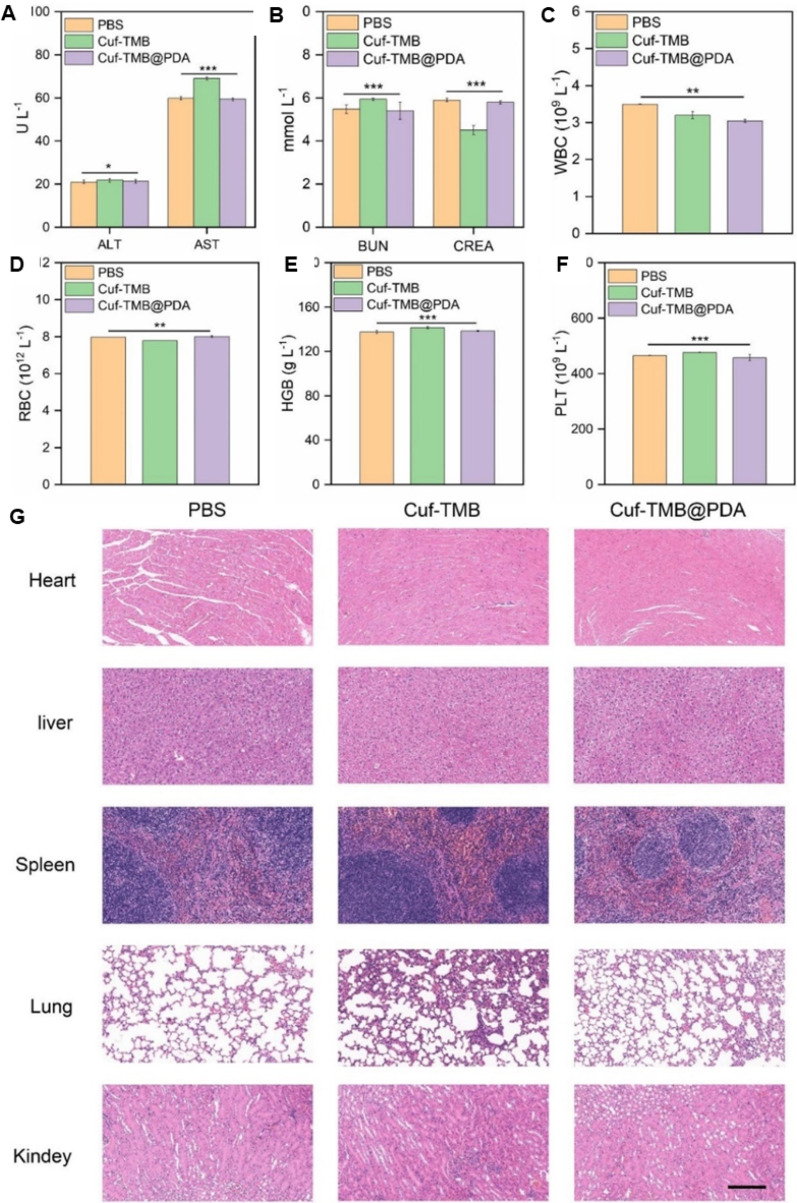
Biosafety evaluation of Cuf–TMB@PDA in vivo. (**A**–**F**) Blood biochemistry and hematology parameters after treatment, including liver function (ALT, AST), kidney function (BUN, CREA), and blood indices (WBC, RBC, HGB, PLT). (**G**) Representative H&E-stained histological images of major organs (heart, liver, spleen, lung, kidney) show no significant pathological abnormalities across PBS, Cuf–TMB, and Cuf–TMB@PDA groups. This figure is adapted from [197, 199–202]

This section critically evaluates the potential toxicology and environmental impacts of these hybrid nanomaterials themselves, including risks arising from metal-bio interfaces, to inform safe translation into clinical wound therapies. The anti-bacterial application of smart hybrid NPs gained the spotlight over conventional nanoparticle synthesis due to their improved stability, bioavailability, sustainable release, antibacterial activity, angiogenesis, regeneration, and so on. Their positive impact is mainly due to the blending of bio-friendly and synthetic elements in hybrid NPs tested in preclinical and clinical settings for wound healing applications. However, thorough testing of smart hybrid NPs’ environmental and potential toxicity needs special attention. Few studies attempted to study the toxicity of hybrid NPs in several unicellular and multicellular organisms such as *Caenorhabditis elegans*, fish, *Drosophila melanogaster*, mice, or rats in various biological applications. For instance, AgNPs are well known for their antimicrobial effects and are mostly used in synthesizing smart hybrid NPs in regenerative medicine. A study that used AgNPs with iron oxide NPs Fe_3_O_4_-NPs in one hybrid structure (Fe_3_O_4_@Ag-NPs) exhibited targeted biomedical applications but also showed toxicity effects in germline apoptosis and delayed egg hatching due to increased ROS accumulation. This study concluded that the high doses of Fe_3_O_4_@Ag-NPs treatment can be toxic and can exhibit harmful effects on living organisms [192]. In contrast, the synthesized MnFe_2_O_4_@poly(*t*BGE-alt-PA) nanocomposite was tested for toxicological properties in Drosophila melanogaster. Upon treatment of the composite to larvae, no abnormal path and altered crawling pattern were observed, indicating that there was no neurotoxicity. Additionally, the composite also doesn’t exhibit structural and phenotypic anomalies, apoptosis, or gut necrosis, indicating that they are safe to be used in biomedical and environmental applications [193]. Therefore, understanding each nanomaterial’s toxicity despite its biomedical application is extremely important to avoid detrimental effects. Copper oxide NPs (CuO-NPs) are widely used in biomedical applications, including antimicrobial agents, and their toxic nature to animals has also been reported. For example, CuO nanomaterials affect organs such as the kidney, liver, and spleen and can reduce the antioxidant response at the plasma level by enhancing malondialdehyde content. Another study also reported that the use of CuO-NPs can affect hepatic, renal, cardiac, and neural organs by altering the structure and physiology of those organs due to increased production of oxidative stress; thus, we need to pay more attention when using this as it can impact humans and related ecosystems [194, 195]. Conversely, compared to organic antimicrobial NPs, the use of inorganic antimicrobial NPs has gained a spotlight due to their low toxicity, eco-friendliness, high stability, and photothermal effect. Moreover, it is also well-known that compared to the use of mono metallic NPs for biomedical applications, the design of bi-metallic or hybrid NPs can also benefit in terms of toxicity. Therefore, a study reported copper oxide and silver oxide co doped with nanocomposites (CuO@AgO/ZnO NPs). This therapeutic nanocomposite enhanced rapid wound healing of *S. aureus* infected wounds by exhibiting tremendous wound recovery along with good hemocompatibility and biocompatibility [196]. However, this study used only hemolytic assay in vitro to measure toxicity and lacks toxicological evaluation in vivo and thus alarming their use in clinical practice. Another study showed polydopamine (PDA) coated copper-amine-based nanoparticle (Cuf-TMB@PDA) can combat bacterial infections and minimize toxicity-related challenges faced by Cu (II) NPs in vitro and in vivo (Fig. 16). With effective antibacterial effect, skin tissue repair and cytocompatibility of Cuf-TMB@PDA [197]. Zein is a biodegradable and biocompatible protein and therefore considered advantageous of using zein in NPs synthesis. Isoliquiritigenin (ISL), a type of flavonoid, has many health benefits but has poor bioavailability and stability and limits its application in clinical studies. The study demonstrated the designed NPs didn’t exhibit adverse effects on acute and sub-acute studies as a result claim to use them in prolonged consumption. The study on ISL@ZLN NPs needs long-term toxicity analysis in vivo for sustainability, and to assess potential dose-dependent and cumulative effects [198].

Generally, AuNPs can be considered safe and widely used alone with other nanomaterials. However, their toxicity can vary largely depending on the physicochemical parameters, type of conjugation used, cells studied, etc. Recently, smart hybrid NPs have been combined with gold and designed to be stimuli-responsive to release the drug sustainably. For instance, thermos-responsive gels conjugated with AuNPs showed the highest antibacterial and wound healing effect, exhibiting prolonged and sustained drug release [183]. Although they showed no skin irritation or skin-related sensitization in rabbits or guinea pigs, their toxicity response for a long time must be conducted to use this material confidentially in clinical practice. In another study, they studied the novel iron-gold-based hybrid NPs.

**Fig. 17:**
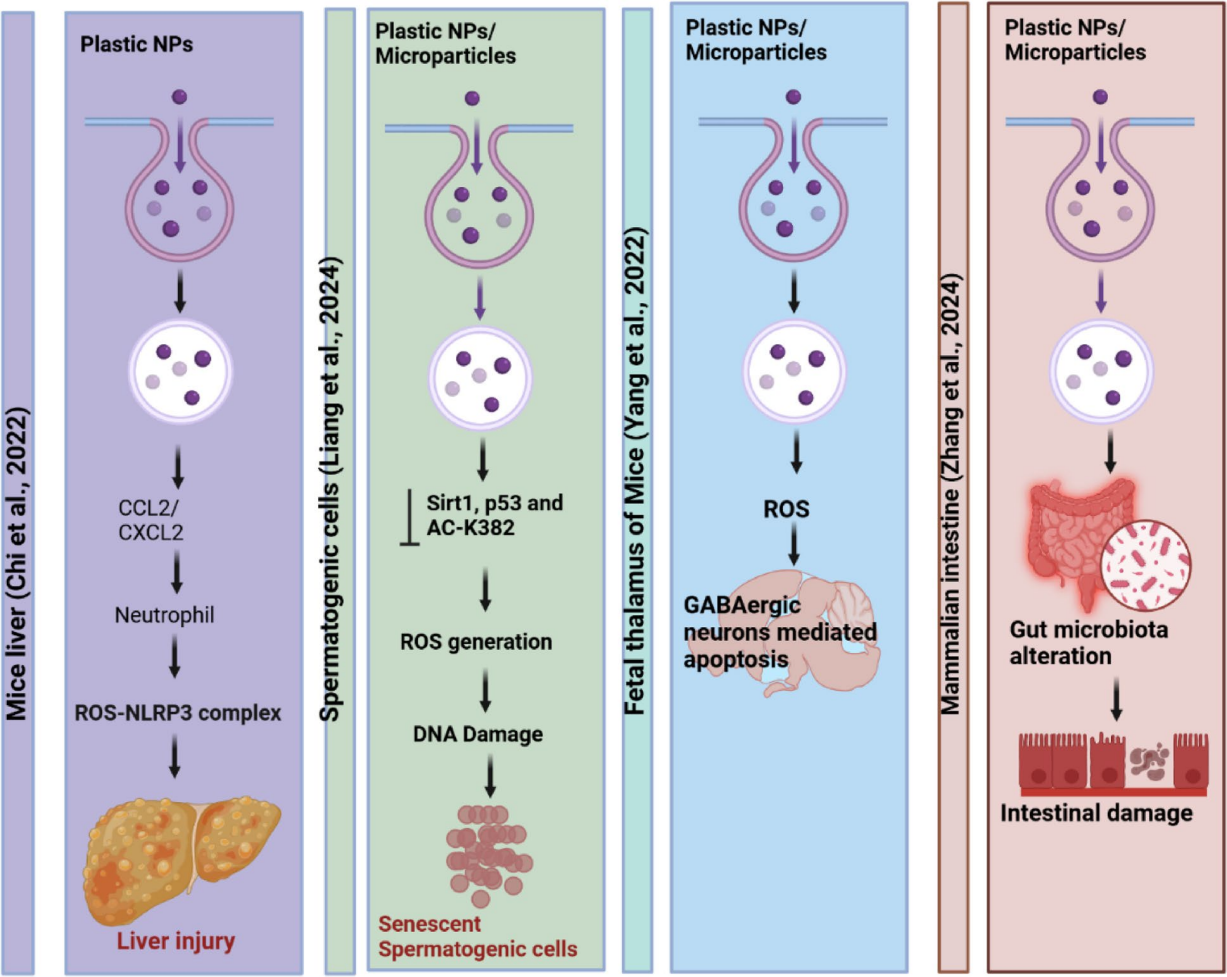
Organ-specific toxicity mechanisms induced by plastic nano- and microparticles This figure outlines the molecular pathways through which plastic NPs and microparticles exert toxic effects on various organs. In the liver, they trigger the CCL2/CXCL2 pathway and ROS–NLRP3 inflammasome activation, leading to neutrophil recruitment and liver injury. In spermatogenic cells, plastic particles disrupt Sirt1 and p53 regulation, induce ROS production, and cause DNA damage, resulting in cellular senescence. In the fetal thalamus, elevated ROS induces apoptosis in GABAergic neurons. In the gut, plastic exposure alters microbiota composition and damages intestinal structure, highlighting widespread organ-specific toxicity associated with plastic particle exposure [208–211]

Although several researchers focused on biomedical applications on various hybrid NPs, environmental degradation is poorly investigated. However, a deep understanding of the degradation process and its impact on environmental health and the food chain is crucial to attaining sustainability. The degradation of polymeric materials is complex and involves several mechanisms, including physical, chemical, and biological pathways. For example, physical mechanism refers to the breakdown of materials upon mechanical forces such as abrasion, tearing, and fragmentation that are known to be induced by external physical stresses such as weathering, friction, and impact. Environmental factors, including temperature and UV, also belong to physical degradation. For instance, the environmental degradability of the tert-butyl peroxybenzoate melt-modified poly(_L_-lactide) (PLLA) was easily degradable at high temperatures and showed poor degradability at temperatures < 4 °C [203]. Plastic pollution including micro and nanoplastics such as polyethylene (PE), polyvinyl chloride (PVC), polystyrene (PS), and polyethylene terephthalate (PET) showed poor resistant degradation properties that can enter into food chain and induce toxicity response. The impact of PS containing hybrid NPs induces toxicity to various organs (Fig. 17). However, to enhance the degradation of these polymers, enzymes in microorganisms, such as laccases, proteases, cutinases, etc., can degrade polymers enzymatically [204]. Variety of microorganisms were reported to have the ability to degrade polyethylene including *Comamonas*, *Pseudomonas*, *Rhodococcus*, *Staphylococcus*, *Streptomyces*, *Bacillus*, *Acinetobacter*, *Aspergillus*,* Cladosporium*,* Fusarium*,* Penicillium* respectively. This decomposing property of microbes is due to the presence of enzymes and co-enzymes that react with polar carbonyl and hydroxyl bonds, which are responsible for the degradation of polyethylene [205]. Currently, adapting enzymatic catalysis to degrade plastic waste is an excellent way to promote sustainability to reach environmental goals and minimize their environmental footprint. Polymers can also undergo degradation upon oxygen availability to undergo photo-oxidative and thermos-oxidative degradation processes. In this scenario, oxygen participated in the production of ROS to enhance the faster degradation process. If the oxygen is absent, then the polymers can take anerobic degradation alternate pathways to continue degradation [206, 207].

**Fig. 18:**
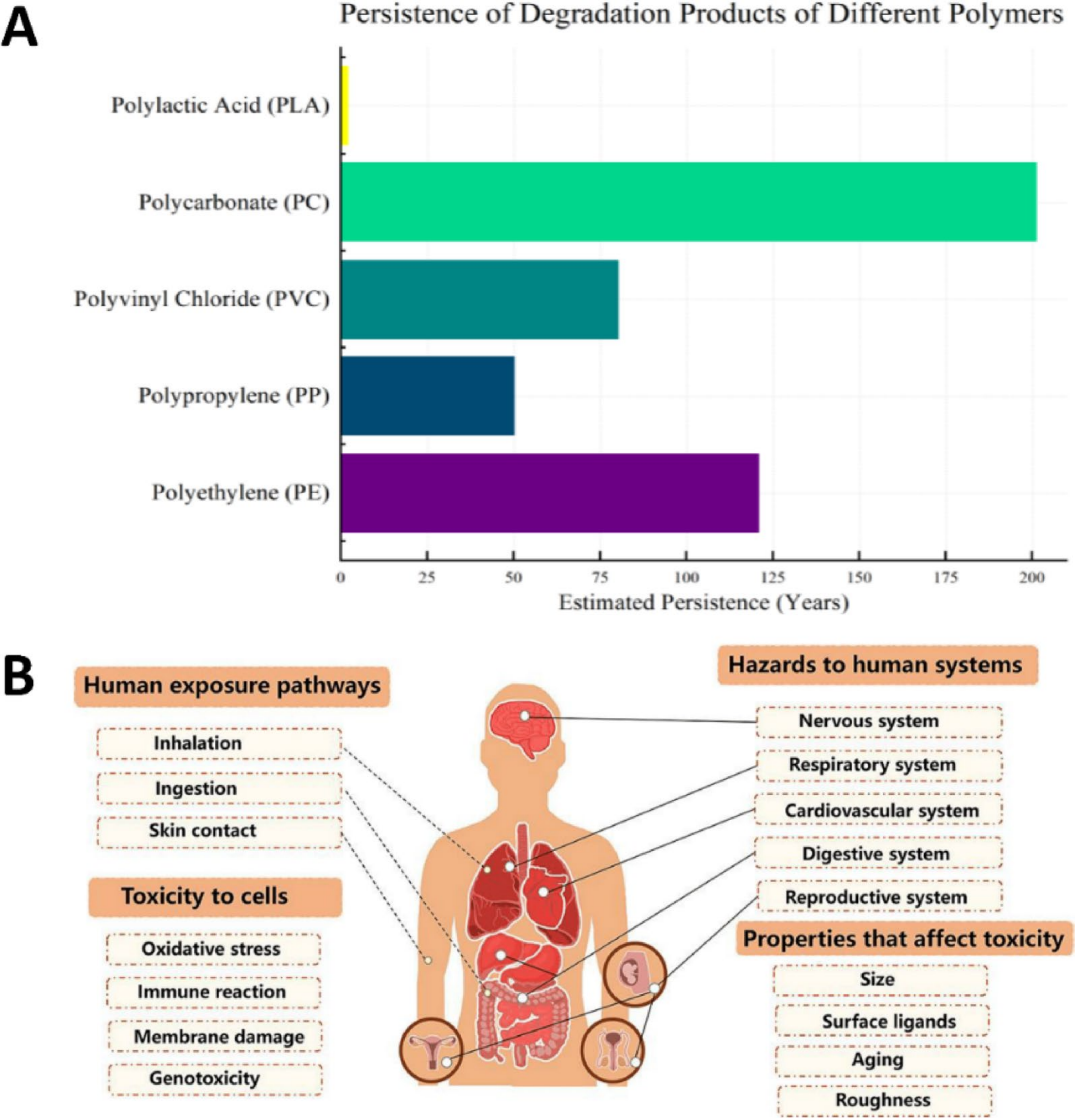
Persistence and toxicity pathways of synthetic polymer degradation products. (**A**) Estimated persistence of degradation products from different polymers shows high stability of polycarbonate (PC), polyethylene (PE), and polyvinyl chloride (PVC), whereas polylactic acid (PLA) degrades rapidly. (**B**) Human exposure to microplastics occurs through inhalation, ingestion, and skin contact, leading to cellular toxicity via oxidative stress, immune reactions, membrane damage, and genotoxicity. These effects manifest as hazards to the nervous, respiratory, cardiovascular, digestive, and reproductive systems, with toxicity influenced by particle size, surface ligands, aging, and roughness. Adapted from [216, 217]

Integration of metal cores with green matrices can increase cytotoxicity and immunotoxicity by exacerbating risks such as ion leaching (e.g., Ag^+^ or Zn^2+^ release) in hybrid systems, such as chitosan-metal composites or AgNPs functionalised with peptides. According to in vitro studies on PBMCs exposed to AgNPs, for example, these hybrids may cause ROS-mediated mitochondrial damage and apoptosis in hepatocytes or immune cells, potentially jeopardising the very microbiome modulation meant for chronic wound healing. Hybrid nanomaterials pose unique reprotoxicity concerns due to their nanoscale size and surface modifications; for example, iron oxide/silver hybrids have been shown to impair the cholinergic system and cause reprotoxicity in Caenorhabditis elegans models, while polystyrene hybrids in mice trigger liver inflammation via ROS-NLRP3 axis-dependent DNA-NET formation (Fig. 18). These findings highlight the need for rigorous preclinical testing to mitigate risks in vulnerable populations, such as diabetic patients with chronic wounds.

The hybrid NPs, especially prepared using synthetic polymers such as polystyrene, when exposed to animal models, exhibited varying toxicity levels, such as developmental abnormalities, increased mortality rates, alteration in gut microbiota, liver damage, reproductive toxicity, and so on [212–214]. Several organ-related toxicities were absorbed using the combination of polystyrene microplastics with zinc oxide NPs (PSMPs + ZnO NPs) in the aquatic food chain from *Chlorella vulgaris* to *Daphnia magna* (Fig. 18). It is well-known that this plastic containing polymeric materials can accumulate in various organs, especially in the liver and kidneys, and cause overall health complications. For instance, when mice are exposed to polystyrene, microplastics are known to impair glucose tolerance and accumulate hepatic lipid deposition in mice [215].

**Fig. 19:**
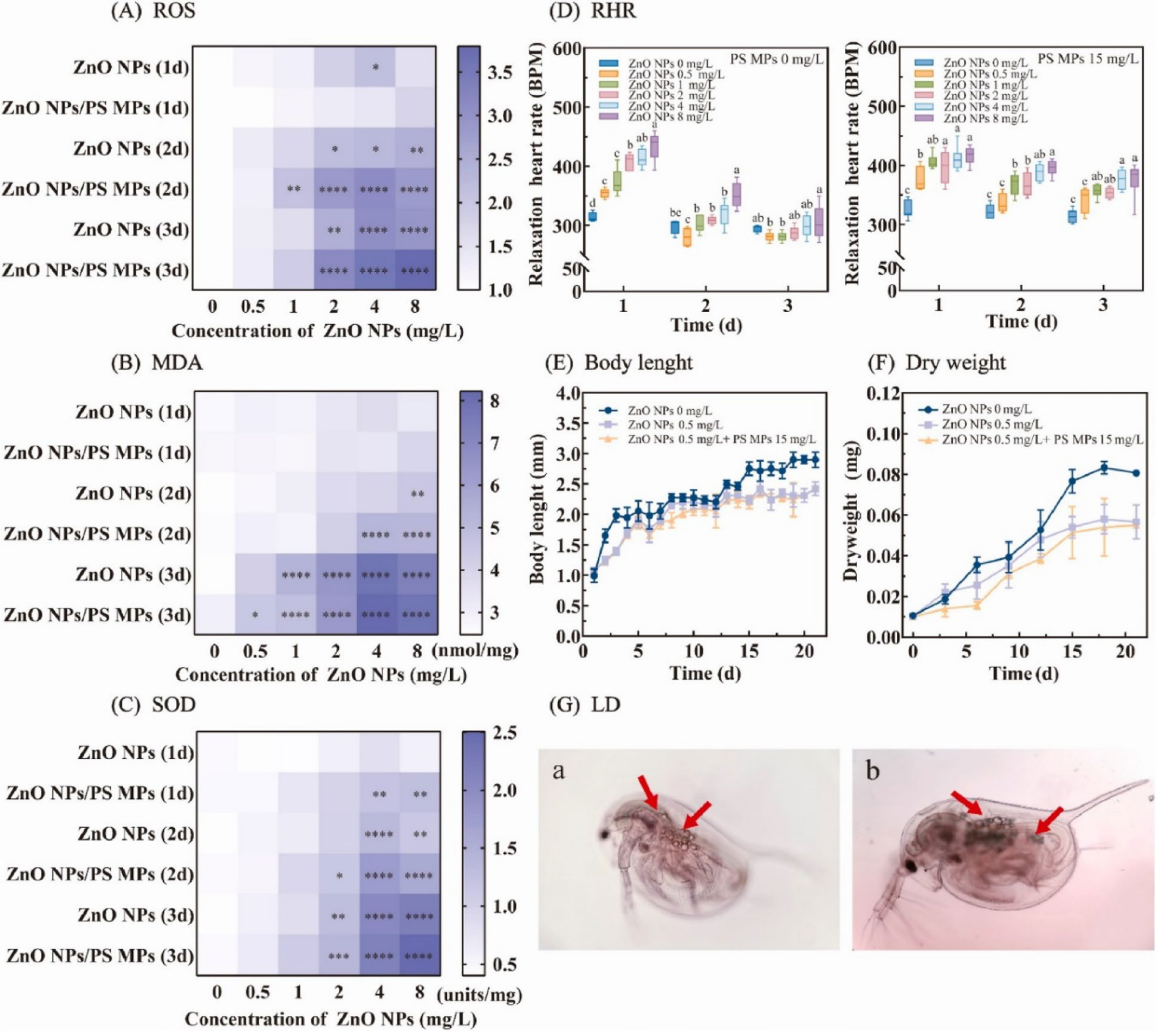
Biochemical and physiological toxicity of ZnO NPs and polystyrene microplastics (PS-MPs) in *Daphnia magna* after dietary exposure. (**A**–**C**) Oxidative stress biomarkers including ROS, MDA, and SOD levels after 1–3 days at different ZnO NP concentrations with/without PS MPs. (**D**) Relaxation heart rate (RHR) Changes over 3 days across treatment groups. (**E**–**F**) Effects on body length and dry weight during 20-day exposure. (**G**) Light microscopy images showing lipid droplet (LD) accumulation (red arrows) in *D. magna* exposed to ZnO NPs and ZnO NPs + PS MPs. This figure is adapted from [224]

Another study demonstrated that exposure to Dr(2-ethyl hexyl) phthalate (DEHP) resulted in increased inflammatory markers, metabolic function disruption, and increased lipid accumulation, thus affecting the liver and inducing liver damage [218]. Similar study was also conducted to evaluate kidney toxicity induced by polystyrene also caused detrimental effects such as enhanced renal inflammation, increased oxidative stress, fibrosis, etc., in mice. Apart from organ damage, these plastic microparticles are also known to exhibit immunotoxicity by increasing pro-inflammatory responses. Microplastics hinder immune responses by altering cytokines, increasing susceptibility to infections, increasing oxidative stress and ROS, and so on [219, 220]. The contamination of microplastics into ecosystems can also increase genotoxicity and carcinogenicity in animal models. Specifically, they are known to cause DNA damage, chromosomal aberrations, development of tumors, etc [221]. They are also known to cause neurological disorders such as anxiety and depression by increasing the mitochondrial damage to neurons and enhancement of disruption of the tight junction of mice [222]. Although these disadvantages exist, understanding degradation mechanisms and molecular pathways of synthetic polymers alone or when combined is crucial and timely for predicting polymer stability and the environmental fate of polymeric NPs (Fig. 19). For instance, the use of C, N-TiO_2_/SiO_2_ was used to degrade microplastics through a photocatalytic degradation process in a pH-dependent Manner, achieving 9.35–16.22% of mass loss [207]. Most of the toxicity studies are carried out using animal models but not on the real situation of the human body, and therefore, large funding must be encouraged for human-related models such as spheroids and organoids to provide deep and accurate insights into using polystyrene-induced possible detrimental effects in the real human model.

Recently, the 3Rs policy was developed to reduce, refine, and replace animal experiments for accessing nanoparticle-induced toxicity. 3D culture of HepG2 liver cells was used to study several NPs such as ZnO, TiO_2_, and AgNPs, indicating that TiO_2_ didn’t exhibit cytotoxicity, whereas AgNPs and ZnO showed mild genotoxicity but not significant. This study also observed the use of 3D culture, as there was significant toxicity variation between 2D and 3D models. For instance, AgNPs are shown to increase toxicity in 2D but not in 3D cultures (EC50 > 30 µg/cm^2^) [223].

Overall, stimuli responsive nanomaterials that are used in antibacterial treatment are not widely studied for clinical application [225]. However, only one nanomedicine called as Visudyne that are stimuli-responsive, were approved by FDA and some others are still in the clinical stage [199]. To make the nanomaterials reach clinical trial and for long-term and sustainable use, simplified formulations must be adapted, and a thorough toxicity profile of the synthesized nanomaterials must be performed for the long term. Moreover, an enormous amount of time, labor, and cost are needed to optimize and improvements needed to translate each stimulus-based NM from the preclinical to clinical stage apart from endogenous triggers such as pH and temperature, as this varies depending from one person to another. Nevertheless, the exogenous stimuli-responsive systems are much easier to control and promising for clinical practice. With the advancement in technologies and with deep research, exploration can loosen up the regulatory laws, and therefore, stimuli-responsive nanomaterial for bacterial infection can have a bright future.

**Fig. 20:**
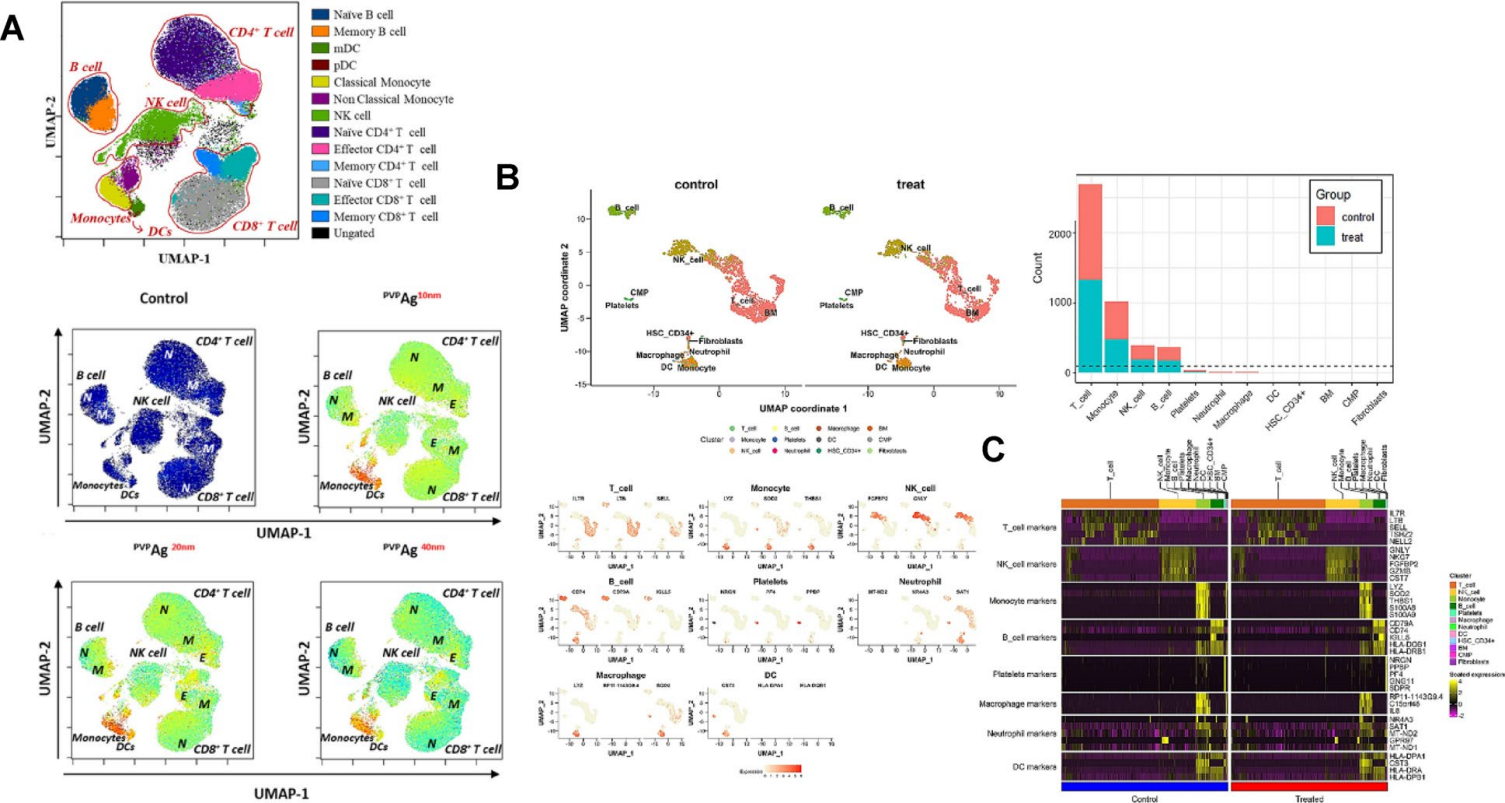
Single-cell phenotypic profiling of immune cell subsets after AgNPs treatment. (**A**) UMAP clustering showing distinct immune cell populations. (**B**) Comparative UMAP plots for control and AgNPs-treated groups with cell type annotation, marker expression, and cell distribution shifts. (**C**) Heatmap analysis of lineage-specific markers across T cells, NK cells, monocytes, B cells, macrophages, neutrophils, and DCs. This figure is adapted from [226, 227]

Additionally, the immunological profile at the single-cell level needs to be investigated to investigate whether nanomaterials induce immune-transcriptional and translational changes upon hybrid NMs exposure. So far, very few research papers have been reported on the immunological response after metal NPs treatment to PBMC cells. For instance, this group demonstrated that upon AgNPs exposure, there was upregulation of cellular metal-ion association with a significant increase in metallothionein genes that didn’t induce a significant inflammatory response, indicating that AgNPs can be safely used (Fig. 20) [226]. Balancing therapeutic efficacy with environmental Sustainability is essential to ensure these Materials are safe for both patients and ecosystems. Very few studies reported the toxicity of NPs using 3D models but not on hybrid NPs, and therefore, future studies are warranted to study the toxicity of hybrid nanomaterials using 3D cultures. Additionally, funding agencies can also consider funding for degradation and toxicity studies that use high dimensional techniques such as siRNA seq, mass cytometry, and special transcriptomics to demonstrate single cell and tissue level toxicity of degradation products. Once all is set, a meaningful understanding of synthetic polymers'potential degradation and toxicity effects can move NPs forward and help them decide to use them safely in clinical practice.

In order to facilitate the safe clinical application of hybrid nanomaterials in regenerative medicine, this warning analysis ultimately emphasises the need for balanced risk assessment and mitigation techniques, such as AI-driven toxicity prediction.

## AI-driven nanomaterial design for personalized therapy

With the growing complexity of smart nanomaterial design, AI has become a vital asset, enabling data-driven optimization and simulation of material behavior. AI technologies are now being leveraged to engineer microbiome-adaptive nanoplatforms with enhanced precision and personalization. AI’s capacity to process and analyze vast datasets and uncover complex patterns has led to significant advances in personalized therapy [228]. AI-driven methodologies have transformed nanomaterial discovery by improving precision and efficiency in cancer and regenerative applications. By predicting nanomaterial interactions with biological systems, researchers can now optimize material properties, resulting in more effective and personalized medical solutions [229]. ML, a subset of AI, plays a central role by accurately forecasting nanomaterial behavior, including their stability, solubility, and biocompatibility across physiological environments. This facilitates safer, more targeted therapeutic designs. The integration of AI tools such as data mining, neural networks, and nanoinformatics marks a key milestone for precision medicine.

Traditionally, nanomaterial design relied on labor-intensive, trial-and-error methods. These approaches were not only time-consuming but also error-prone. AI-powered systems now simulate multiple design scenarios and forecast outcomes, significantly reducing the need for repetitive laboratory testing. This is particularly valuable in regenerative medicine, where timely interventions can greatly influence recovery. AI expedites the screening of candidate materials and configurations, improving efficiency and enabling faster therapeutic breakthroughs.

**Fig. 21:**
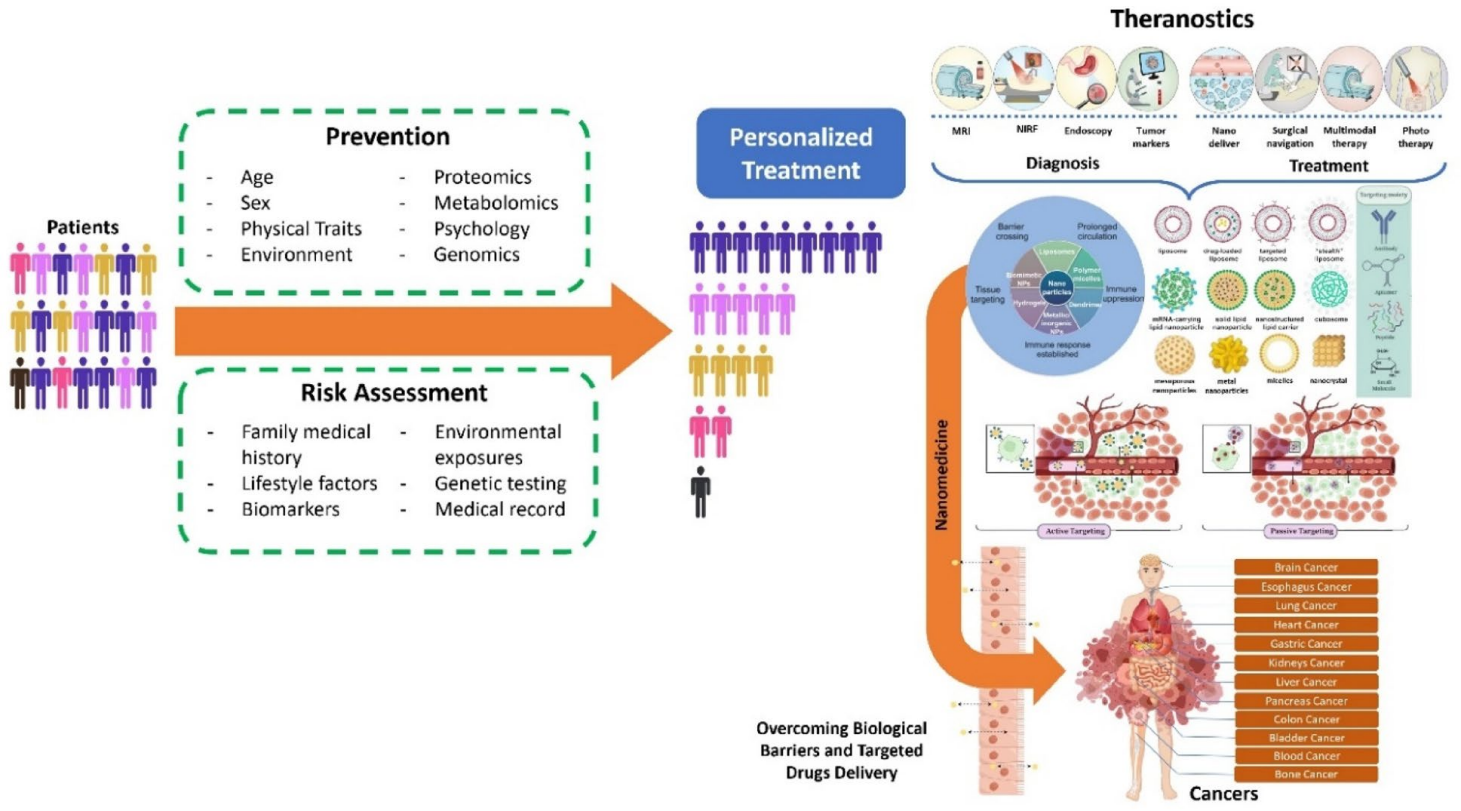
Nanomedicine utilizes nanocarriers (e.g., liposomes, micelles, NPs) to target malignant cells with precision, reducing harm to healthy tissue. Passive targeting leverages enhanced permeability and retention effect, while active targeting uses ligands to bind cancer-specific receptors. This strategy improves outcomes in tumors like brain, lungs, liver, and colon cancers

Personalized medicine is essential for managing chronic infections, where factors such as multidrug-resistant pathogens, biofilms, and diverse immune responses complicate treatment [230]. Tailoring therapy to a patient’s unique profile enhances effectiveness and reduces the risk of resistance. Nanorobotics guided by AI can model nanoparticle behavior, drug release kinetics, and therapeutic responses. This highlights the potential of AI-driven nanoinformatics in addressing treatment complexity and improving patient outcomes (Fig. 21).

AI enhances personalized therapy through data integration across genomics, proteomics, metabolomics, and clinical records [231]. This comprehensive analysis allows researchers to identify biomarkers linked to disease states and responses, guiding the design of personalized nanomaterials [232, 233]. For example, AI can help develop nanocarriers that target infection-specific receptors, minimizing off-target effects. Moreover, AI systems can dynamically adapt therapies in real time, refining treatment regimens based on ongoing monitoring a crucial capability in managing fluctuating conditions such as chronic infections.

### Role of AI in nanomaterials

AI, particularly through ML algorithms, has become a critical tool for predicting the biocompatibility and performance of nanomaterials. This advancement stems from AI’s ability to process complex, multi-dimensional datasets collected from biological, clinical, and computational sources [234]. These models provide predictive insights into nanomaterial interactions with living systems, offering significant value in applications such as drug delivery, tissue engineering, and diagnostic imaging. Understanding how the nanomaterials behave in biological systems is essential for their safe and effective use. ML models, trained on extensive in vitro and in vivo datasets, can anticipate cellular uptake, toxicity profiles, and biodistribution with increasing accuracy [234]. This predictive capability accelerates material screening, reducing the need for exhaustive experimental iterations.

Moreover, the integration of AI with high-throughput screening platforms like automated cell culture and robotic analysis has substantially expedited biocompatibility assessments. These platforms generate large-scale datasets that AI can rapidly analyze to identify promising nanomaterial formulations suited for specific therapeutic contexts. Such synergy is especially impactful for personalized medicine, where individual variability in physiological responses can alter nanomaterial behavior.

**Fig. 22:**
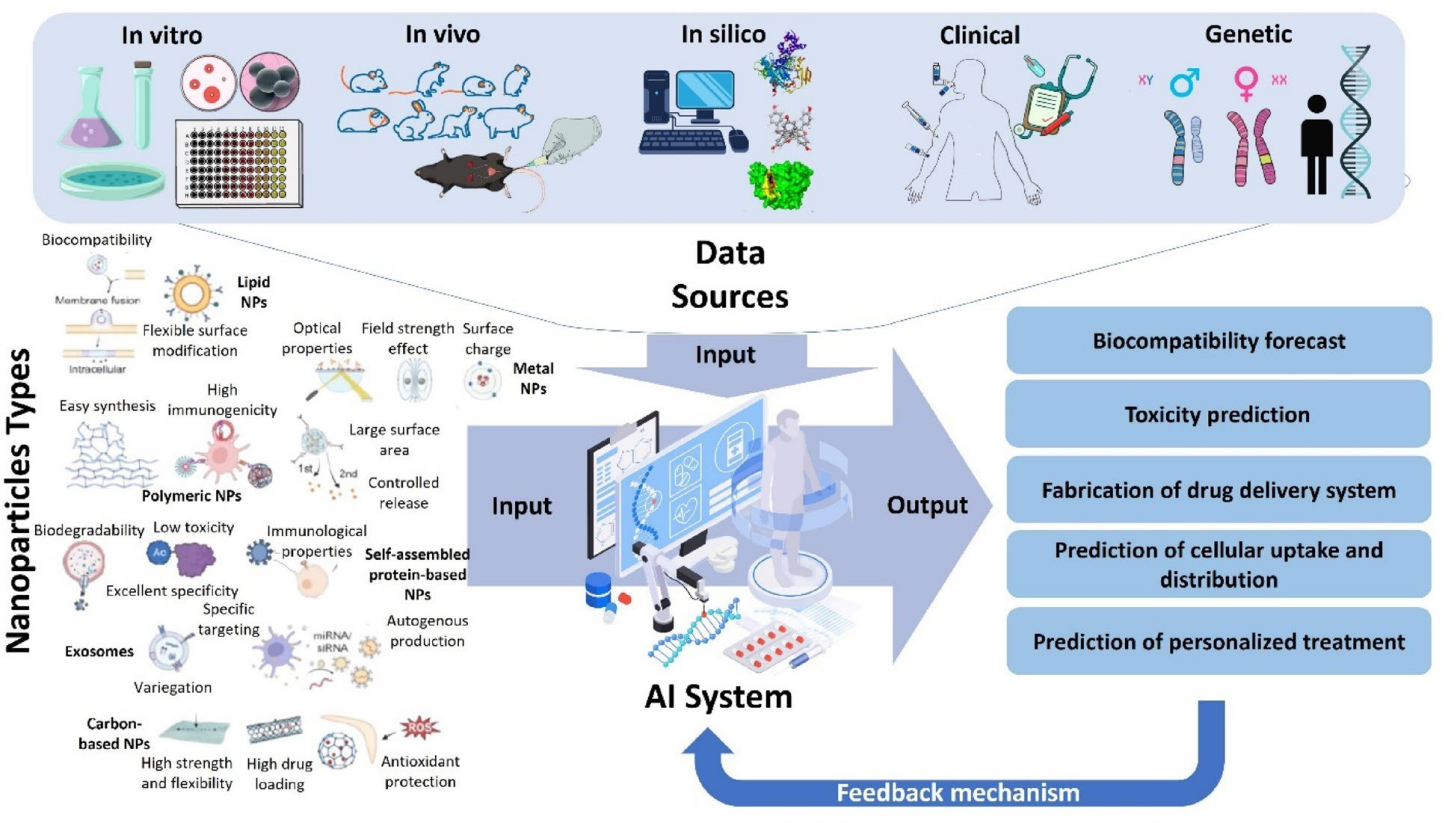
illustrates how AI leverages diverse data sources including in vitro, in vivo, in silico, and clinical studies to forecast biocompatibility, optimize drug delivery systems, and guide patient-specific therapy. Different classes of NPs, such as lipid-based, metallic, polymeric, carbonaceous, and protein-derived materials are modeled for tailored applications, ensuring a holistic view of their biomedical potential

AI models can also incorporate pharmacokinetic and pharmacodynamic parameters to predict nanoparticle absorption, distribution, metabolism, and excretion (ADME), helping identify potential toxicities early in development [232]. This not only minimizes trial-and-error experimentation but also supports rational design decisions that prioritize both efficacy and safety [232]. By harnessing AI’s capabilities, researchers can streamline the development process, bringing innovative nanomedicines to market more rapidly, ultimately benefiting patients and enhancing healthcare outcomes (Fig. 22).

AI’s influence in modeling and predicting the progression of chronic infections is equally transformative. These advanced models utilize diverse datasets, including clinical records, environmental factors, and even digital epidemiology data, to forecast how infections evolve. In chronic infection scenarios, AI can analyze patient-specific information to uncover complex interactions between pathogens and host immune responses, offering critical insights that enable timely and effective interventions. Predictive models powered by AI can detect early signs of infection escalation or remission, allowing clinicians to proactively adjust treatment plans and strategies [235]. Furthermore, these models can help identify potential outbreak hotspots, enhancing public health preparedness and resource allocation. Beyond infection tracking, AI can simulate therapeutic scenarios to evaluate the outcomes of various treatment strategies. This capability supports the development of personalized protocols, improving efficacy while minimizing side effects [231, 235, 236]. The integration of AI-driven models into healthcare represents a major advancement in managing infectious diseases. Through predictive analytics, clinicians can make informed decisions, enhance treatment methodologies, and improve patient outcomes, especially in the context of chronic or difficult-to-treat infections (Table 5).

AI also enables optimization of therapeutic agent release profiles an essential component of personalized medicine. AI-based models assess patient-specific parameters, including disease progression, drug kinetics, and real-time physiological responses, to tailor drug administration schedules. These models ensure therapeutic agents are delivered at optimal times and doses, maximizing efficacy while minimizing systemic toxicity. For instance, AI can aid in designing nanocarriers that respond to specific biomarkers, releasing drugs only when defined conditions such as changes in pH, enzyme levels, or inflammatory markers are met. Biomarkers, which include proteins, nucleic acids, and metabolites, provide critical insights into a patient’s physiological state. AI-guided drug delivery systems that respond to these markers enable precise, adaptive treatment strategies.

**Table 5:** Summary of AI/ML applications in nanomaterials

Application area	AI/ML methods	Features	Goal	Key outcomes
Biocompatibility prediction	SVM, Random Forest	Physicochemical + *in vitro/in vivo* datasets	Classify safe vs. toxic nanomaterials	High accuracy in toxicity prediction
High-throughput screening	AI integrated with automation	Large-scale experimental datasets	Identify promising formulations	Accelerated material screening & discovery
Pharmacokinetics & ADME modeling	Bayesian models, Regression	Pharmacokinetic/Pharmacodynamic datasets	Predict absorption, distribution, metabolism, excretion	Reduced trial-and-error, early toxicity detection
Chronic infection modeling	Predictive analytics, Deep Learning	Clinical + epidemiological datasets	Forecast infection progression & treatment response	Enabled early intervention & outbreak tracking
Drug release optimization	AI-based optimization models	Patient-specific biomarkers, disease profiles	Tailor release profiles & dosing schedules	Adaptive, precise therapy with minimal side effects
Intelligent drug delivery	AI with biosensor integration	Real-time physiological monitoring data	Dynamic dose adjustment	Revolutionized personalized medicine with real-time control

The table highlights how different AI and machine learning methods leverage diverse datasets (in vitro, in vivo, in silico, and clinical) to predict biocompatibility, model pharmacokinetics, accelerate high-throughput screening, optimize drug release, and enable intelligent drug delivery. These approaches support safer, more effective, and personalized nanomedicine development

Additionally, AI contributes to the development of intelligent drug delivery platforms equipped with biosensors that monitor physiological metrics in real time. These systems dynamically adjust dosing based on patient conditions, improving therapeutic precision and reducing adverse effects. This is particularly beneficial for managing fluctuating diseases such as cancer or chronic inflammation, where drug needs may vary over time. The implementation of AI-powered drug delivery systems thus holds the potential to revolutionize personalized medicine resulting in more effective treatments, minimized side effects, and significantly improved patient outcomes. By integrating predictive analytics and real-time physiological monitoring, such systems pave the way toward truly individualized care.

### Patient-specific customization

AI has shown significant promise in customizing nanomaterials to specifically target biofilm-associated infections. By analyzing key biofilm characteristics such as composition, density, and developmental patterns, AI algorithms can help design nanomaterials optimized for deep penetration and structural disruption [237, 238]. This precision-based strategy enhances antimicrobial efficacy while reducing the risk of resistance, by ensuring that nanomaterials are meticulously engineered to dismantle biofilm architecture and neutralize embedded pathogens.

**Fig. 23:**
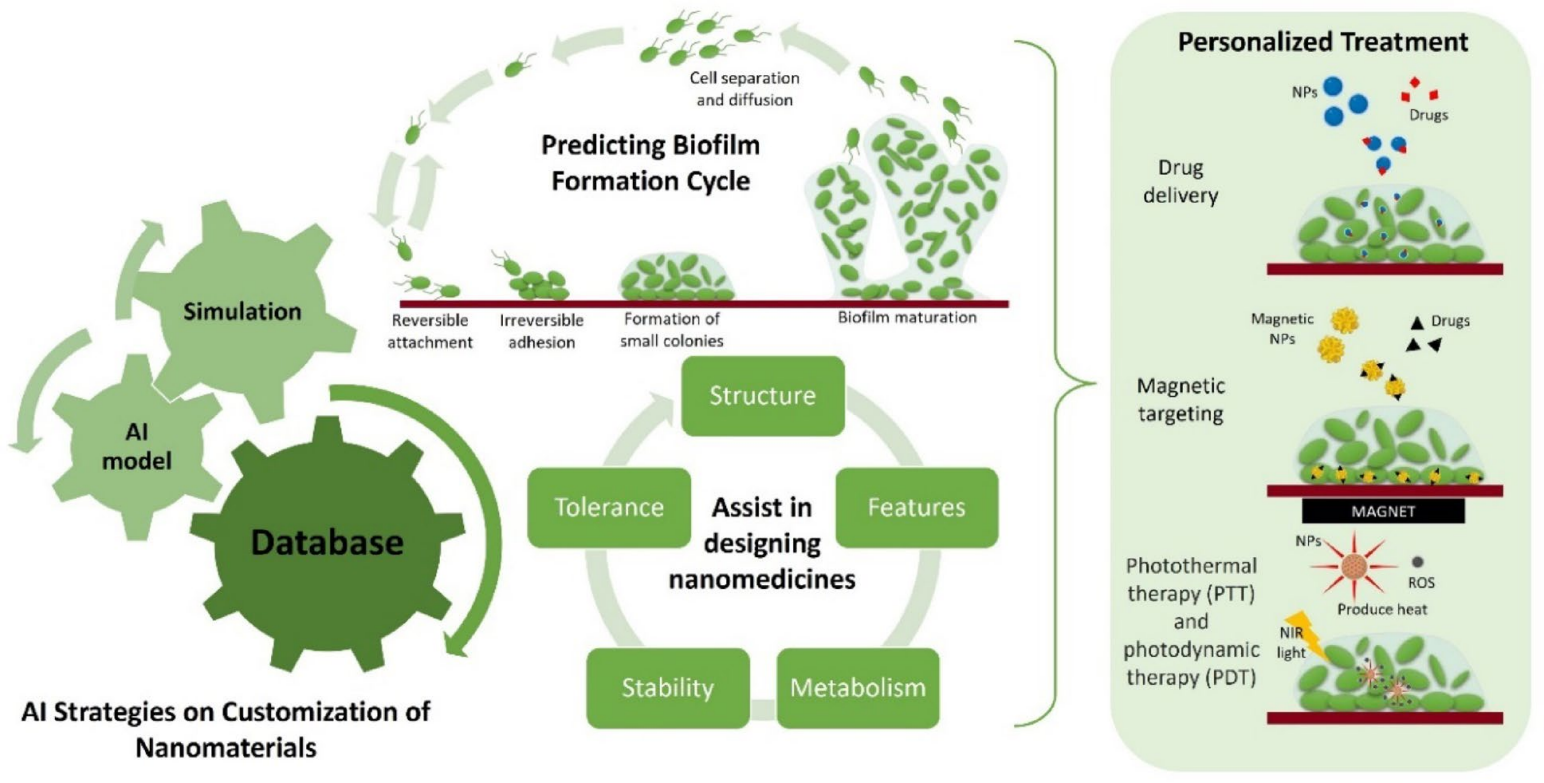
AI-based models and simulations are used to optimize cancer biofilm therapy by targeting various stages of biofilm development: reversible attachment, irreversible adhesion, colony formation, and biofilm maturity. AI aids in personalizing nanomedicine tactics such as drug delivery, magnetic targeting, and photothermal or photodynamic therapy, improving the success of interventions in biofilm-associated tumors

Beyond structural targeting, AI can simulate complex microenvironments within biofilms, including ECM components and microbial communication pathways, to further refine therapeutic strategies. These simulations support the design of nanomaterials that not only improve penetration but also act on multiple functional components of the biofilm, enhancing therapeutic durability [239]. Additionally, AI-powered models can predict biofilm responses to various treatment modalities, enabling adaptive and informed therapeutic planning (Fig. 23).

This holistic integration of AI and nanotechnology not only improves immediate antimicrobial outcomes but also aligns with long-term goals in sustainable infection control. Moreover, recent developments extend AI’s role to customizing treatment strategies based on inflammatory markers, which are pivotal in managing chronic infections and systemic diseases [239]. AI can process complex omics data, including genomic, proteomic, and metabolomic layers, to identify biomarkers linked with inflammation and stratify patients for optimized anti-inflammatory therapy [240].

Such individualized models allow clinicians to select the most effective anti-inflammatory agents and fine-tune dosage regimens to minimize side effects while maximizing therapeutic efficacy. Continuous real-time monitoring of inflammatory markers through AI-integrated platforms enables dynamic treatment adjustments. For instance, Peng et al. (2021) demonstrated how AI systems can modulate therapy in response to fluctuating inflammation levels [236]. Integrating data from wearable biosensors, these systems enable timely detection of changes in a patient’s inflammatory state, allowing prompt therapeutic interventions that prevent complications and improve outcomes.

AI also plays a crucial role in adapting nanomaterials for tissue-specific delivery. Since each tissue type exhibits unique traits such as vascularization, immune profile, and biomechanical properties, tailored approaches are essential for optimal treatment. AI models can evaluate these physiological differences and guide the design of nanomaterials suited for specific tissues. For example, nanomaterials for hepatic applications must be engineered to withstand the liver’s high vascularity and robust immune surveillance, ensuring accurate delivery and minimizing immunogenicity [241]. In neurological applications, AI facilitates the creation of nanosystems capable of traversing the blood-brain barrier, an otherwise formidable obstacle, by optimizing surface charge, ligand chemistry, and size parameters. Similarly, for cutaneous applications, AI aids in predicting how nanomaterials will interact with distinct skin layers, adjusting particle properties for effective penetration and sustained therapeutic release. This is particularly valuable in chronic wound management and dermatological infections.

Moreover, AI enables the simulation of dynamic interactions between nanomaterials and biological tissues, allowing iterative refinement of properties such as particle size, surface chemistry, and drug release kinetics. This feedback-driven optimization ensures that nanomaterials remain compatible with the evolving tissue environment, enhancing both therapeutic efficacy and precision. As a result, off-target effects and systemic toxicity are significantly minimized. Building on this foundation, the next section examines how AI empowers nanomaterials to respond dynamically in real time to physiological changes, enabling precision interventions in fluctuating disease conditions. These adaptive capabilities, further illustrated through real-world case studies, underscore AI’s pivotal role in advancing intelligent nanomedicine.

### Dynamic response systems

AI enables real-time modulation of nanomaterial behavior by integrating patient-specific data streams with ML algorithms. This capability allows nanocarriers to dynamically adapt their therapeutic profiles in response to physiological changes, which is particularly important for chronic infections or inflammation-prone conditions where disease states evolve rapidly [242]. Advanced biosensors can track physiological variables such as pH, temperature, and disease-specific biomarkers. These data are continuously analyzed by AI systems to inform therapeutic decisions and adjust nanomaterial parameters accordingly, such as modifying surface chemistry, altering drug release rates, or changing biodistribution patterns [243]. For instance, if a localized infection site becomes more acidic, AI systems can trigger a faster release of antimicrobial agents to counteract the inflammatory environment.

Real-time feedback platforms also leverage wearable technologies and implantable devices to gather continuous health data. AI models process these signals to detect early signs of disease progression, enabling preemptive therapeutic adjustments. A practical example is AI-guided insulin regulation in diabetes management, which could be translated to other drug delivery platforms using nanocarriers for dynamic dosing [242, 243]. Beyond physiological monitoring, AI contributes to therapeutic adaptation by analyzing longitudinal clinical data, such as genetic markers, patient histories, and immune profiles [244]. This allows for context-specific treatment adjustments. For example, AI can identify early indicators of antibiotic resistance and recommend alternative combination therapies before clinical symptoms worsen [241]. In the context of AMR, AI identifies resistance-associated genes and optimizes antimicrobial selection through predictive analytics [245]. This is critical in designing multifunctional nanoplatforms capable of bypassing or neutralizing resistance mechanisms. Additionally, AI can suggest synergistic drug combinations and model the pharmacodynamics of each agent within the infected tissue microenvironment. AI also supports antimicrobial stewardship by optimizing dosage, scheduling, and agent selection tailored to pathogen profiles and patient response. This capability enhances therapeutic precision while minimizing side effects and limiting resistance development. The integration of dynamic response systems with AI-powered decision-making paves the way for intelligent nanomedicine that is responsive, personalized, and context-aware. These adaptive capabilities are increasingly being validated through industry-led innovations, which are explored in the next section.

### Case studies

Several companies and research groups have demonstrated the transformative potential of AI in advancing nanomedicine from drug discovery to real-time therapeutic optimization. Insilico Medicine developed a novel oncology drug candidate using a deep learning platform for target identification and molecule generation. The candidate is now in Phase II clinical trials, representing a significant reduction in the traditional drug discovery timeline [242]. XtalPi, in collaboration with Eli Lilly, applies AI and quantum physics to predict molecular properties, including crystal structures of small molecules. These insights are directly applicable to nanoparticle design, particularly in improving drug loading efficiency and stability for therapeutic formulations [242]. CytoReason models immune system dynamics using AI to simulate patient-specific responses to therapies. In partnership with Pfizer, these simulations have refined treatment selection for autoimmune conditions an approach adaptable to immunomodulatory nanomaterials [242]. Exscientia introduced DSP-1181 for obsessive-compulsive disorder (OCD) using AI to accelerate compound screening and preclinical optimization. The company’s platform integrates chemistry, biology, and clinical data to shorten the path from molecule design to clinical validation. Benevolent AI identified therapeutic leads for amyotrophic lateral sclerosis (ALS) by mining multi-omics datasets with its AI engine. Their approach highlights how complex biological data can inform nanoparticle design for targeting neurodegenerative pathways.

#### Ongoing research

Recent literature continues to emphasize the pivotal role of AI in driving innovation across the nanotechnology landscape, with diverse applications in material synthesis, biocompatibility enhancement, predictive modeling, and clinical translation. Hrvat et al. (2021) provides a comprehensive review highlighting the integration of AI in designing and synthesizing nanomaterials with tailored physicochemical characteristics. Their findings underscore how AI can accelerate the development of nanostructures for medical diagnostics and therapeutic delivery, significantly improving design efficiency, functionality, and safety profiles [246]. In a related contribution, Liu et al. (2021) investigated how ML algorithms can predict the behavior of nanomaterials in complex biological environments. Their study illustrates the critical importance of modeling nanoparticle biological interactions such as cellular uptake, immune evasion, and in vivo degradation patterns early in the design phase. Such predictive insights are invaluable in minimizing trial-and-error experiments, ultimately supporting the rational development of safer and more effective nanotherapeutics [246].

Nandipati et al. (2024) examined the role of AI in enhancing nanomaterial synthesis. Their work demonstrates how ML models can fine-tune reaction parameters and process conditions to ensure batch consistency, reproducibility, and high-quality outputs in nanoscale production [247]. This is especially relevant for translating lab-scale formulations into clinical-grade materials, where quality control and scalability are often major bottlenecks. Advancing further, Zhu and Li (2023) explored intelligent material design by integrating AI with large chemical databases to discover new material compositions and architectures. Their work focuses on algorithm-driven synthesis pathways that reduce redundancy and cost, accelerating the innovation cycle across both biomedical and industrial applications [248]. Similarly, Gadzhimagomedova et al. (2022) highlighted how AI can address longstanding challenges in nanofluid research and catalysis by modeling thermal behavior and surface reactivity at the atomic level. This type of mechanistic insight enables better control over nanoparticle functionality in real-time applications, such as targeted drug delivery and biosensing [237]. Complementing these studies, Guo et al. (2021) investigated AI’s role in mechanical material design, demonstrating how deep learning techniques can predict structural behavior under various stress conditions and discover previously unknown performance mechanisms. Such approaches can be extrapolated to biomedical nanomaterials, especially those intended for tissue engineering, implant coatings, or dynamic therapeutic environments [242]. Together, these ongoing research efforts showcase how AI is reshaping the way nanomaterials are conceived, fabricated, and applied. By bridging computational intelligence with materials science, researchers are moving toward a future where nanomedicine is not only more personalized and predictive but also more robust and translatable across diverse clinical settings.

### Closed-loop regenerative systems

Closed-loop regenerative systems represent an advanced therapeutic model that integrates AI with real-time biological feedback to continuously optimize treatment interventions. These systems gather data from diverse sources including infection sites, patient physiology, nanomaterial behavior, and microbiome status and feed it into adaptive algorithms that tailor therapeutic strategies in real time [249]. This continuous feedback loop supports dynamic modifications in drug release profiles, surface chemistry, or dosing schedules, ensuring that treatments evolve in step with disease progression and individual patient responses. By leveraging sensor technologies and AI analytics, such systems enable real-time surveillance and precise adjustment of nanomaterial properties. For instance, smart NPs can be engineered to respond to environmental triggers, such as local pH changes or the presence of specific inflammatory biomarkers, by modifying their surface charge or releasing drugs at adjusted rates. This level of customization is particularly beneficial for chronic infections, where the biological environment is dynamic and conventional static treatment regimens often fail [237].

**Fig. 24:**
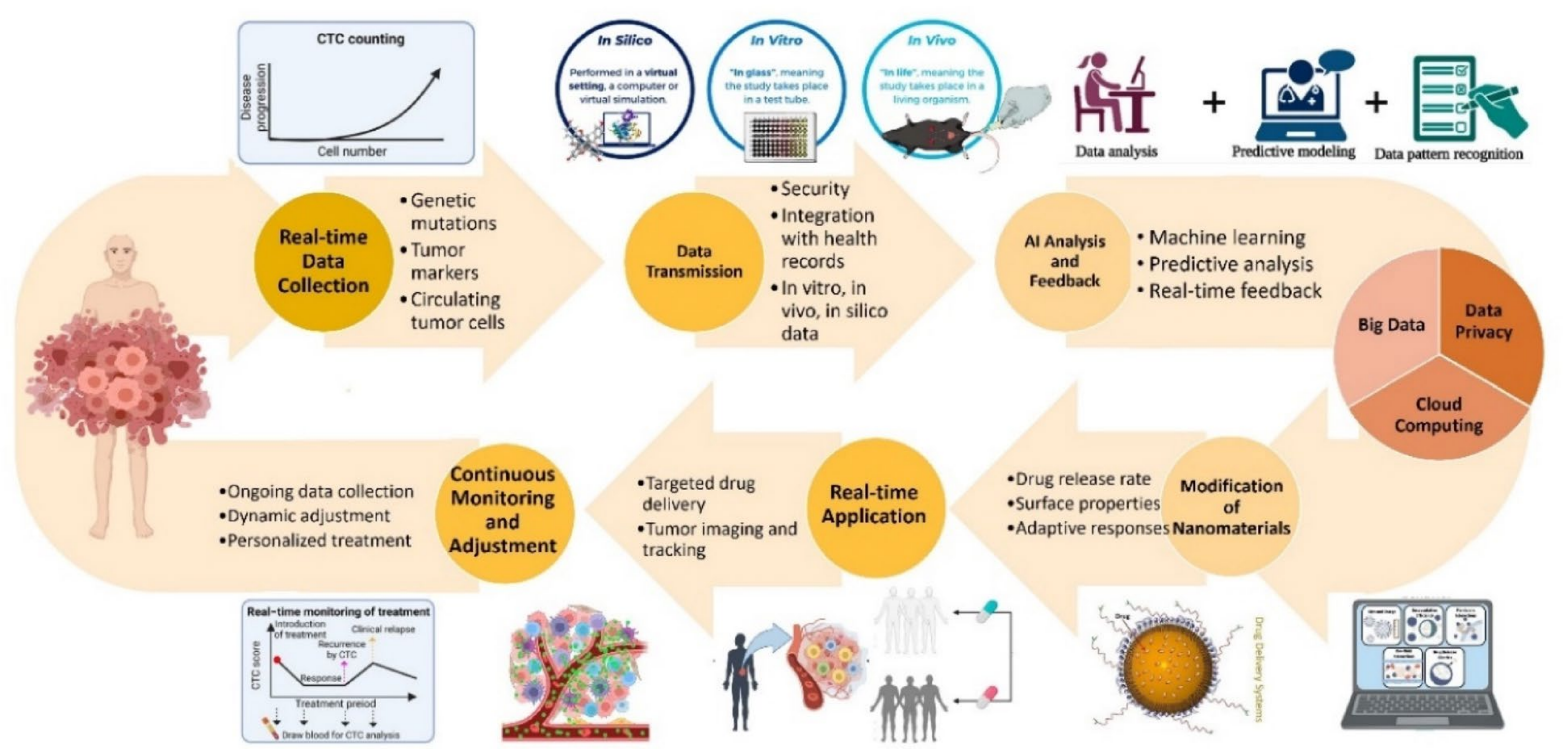
AI-driven closed-loop regenerative systems continuously gather and evaluate data from malignant sites, nanomaterial responses, and patient conditions to adjust therapeutic interventions in real time. These systems adapt treatments by analyzing changes in genetic mutation, specific tumor biomarkers, and circulating tumor cells, ensuring enhanced therapeutic efficacy. ML algorithms process patient data to forecast optimal treatments, while also detecting early signs of drug resistance to enable timely adjustments. Continuous monitoring allows AI systems to update models with new data, maintaining the effectiveness of personalized treatments over time

A key innovation in these systems is their reliance on adaptive treatment strategies (Fig. 24). ML models analyze individual patient data ranging from genetic and proteomic profiles to microbial signatures and use pattern recognition to optimize therapeutic delivery [237]. If, for example, a patient’s infection exhibits a dense biofilm structure that impairs drug diffusion, AI can suggest alterations in nanoparticle composition or functionalization to enhance penetration. Similarly, continuous analysis of inflammation markers enables timely adjustments in dosing schedules or the selection of more targeted anti-inflammatory agents [250]. Beyond personalization, these systems play a critical role in anticipating and counteracting drug resistance. AI-driven analytics can detect early signs of emerging resistance through trends in therapeutic response, enabling clinicians to adjust regimens preemptively. This might involve switching to alternative agents, modifying dosages, or implementing combination therapies specifically chosen to overcome identified resistance mechanisms [237]. The strength of closed-loop systems lies in their capacity for continuous adaptation. As a patient’s condition evolves, the AI algorithm updates its predictive models using newly acquired data from clinical observations to molecular readouts ensuring that therapy remains aligned with the current disease state [250]. For instance, if infection severity increases, the system may escalate therapeutic payloads or adjust the release kinetics of nanocarriers to meet the heightened demand. This approach ensures a resilient, patient-responsive framework that outperforms traditional fixed regimens. Moreover, this model benefits from integrating multiple data streams, including patient-reported outcomes, wearable sensors, and laboratory diagnostics. This holistic view enhances decision-making accuracy and refines therapeutic protocols to maintain efficacy over time [251]. Ultimately, AI-powered closed-loop regenerative systems are positioned to redefine infection management by offering intelligent, flexible, and patient-specific treatment architectures.

### Technical and practical implementation

In recent years, considerable strides have been made in integrating sensors with AI and nanomaterial-based feedback systems. Nanoscale materials significantly improve sensor performance by enhancing sensitivity and specificity, allowing AI systems to better detect and interpret biological and environmental signals. This synergy has led to highly responsive AI technologies that find utility in diverse sectors, particularly healthcare and environmental monitoring. For instance, AI-integrated nanosensors are increasingly used to monitor physiological parameters such as glucose, pH, and temperature in real time, enabling dynamic modifications to treatment protocols. Their precision ensures that subtle fluctuations in patient physiology are detected early, facilitating timely and accurate interventions [246]. Beyond clinical settings, these AI-nanosensor platforms are proving vital in environmental monitoring. A notable example includes their deployment in detecting and managing oil spills in marine ecosystems [252]. Such versatility underscores their broader applicability and the transformative impact of AI-guided nanosensing technologies.

However, translating these technologies from laboratory settings to clinical practice remains a major hurdle. Challenges include the high cost of production, protracted clinical trial timelines, and the stringent safety and regulatory requirements that must be met. Ensuring biocompatibility and avoiding immune system activation are critical; thus, thorough preclinical validation is essential. Additionally, while laboratory-scale nanomaterial synthesis methods are well-established, scaling them up for mass production without compromising consistency and quality remains difficult [252]. Addressing these obstacles requires collaborative efforts among researchers, clinicians, regulatory bodies, and industry stakeholders. Such partnerships can facilitate the development of standardized protocols for nanomaterial characterization, production, and clinical validation. Interdisciplinary collaboration will also support the seamless integration of AI-driven systems within existing healthcare infrastructures, ensuring that these technologies are both effective and practical [252].

Regulatory frameworks surrounding AI in healthcare present additional complexities. Ensuring transparency, algorithmic fairness, data privacy, and robust cybersecurity is critical for maintaining trust and meeting compliance standards. Comprehensive validation of AI algorithms across diverse patient populations is necessary to avoid biased outcomes and to confirm reliability under varied clinical scenarios [246]. Engaging regulatory agencies early in the development pipeline can streamline approval processes and ensure that innovations meet evolving standards. Continuous dialogue between developers and regulators will be essential to adapting policies that keep pace with rapid technological advancements.

### Patient-centric benefits

AI-driven approaches in regenerative medicine offer tangible benefits by minimizing the frequency of medical interventions and tailoring treatment protocols to individual patient profiles. Through advanced ML models, AI can analyze vast datasets to predict the most effective therapeutic strategy, thereby reducing the need for repeated procedures [228]. This proactive treatment planning enhances patient comfort, lowers healthcare burdens, and improves overall well-being. For example, in the management of chronic infections, AI can detect subtle indicators of disease recurrence before clinical symptoms emerge. Real-time monitoring enables healthcare providers to adjust drug dosages or introduce combination therapies promptly, thereby maintaining disease control without requiring aggressive or frequent interventions. This predictive capability extends to post-treatment care, where AI assists in early relapse detection and rapid corrective measures, preserving the efficacy of therapy and ensuring sustained patient recovery [228].

Furthermore, AI contributes to the real-time optimization of therapeutic outcomes. By continuously evaluating patient responses, AI can dynamically modify drug delivery systems such as adjusting release rates or therapeutic concentrations based on metabolic feedback ensuring that treatments remain both effective and safe [253]. This is particularly beneficial in personalized medicine, where inter-patient variability in drug metabolism and immune responses can greatly influence therapeutic success.

AI’s power also lies in synthesizing multidimensional data, such as genomics, lifestyle, and microbiome profiles, to create highly individualized treatment plans. These personalized regimens not only improve efficacy but also reduce adverse reactions by avoiding unsuitable therapies [228]. For instance, AI can identify biomarkers predictive of therapy response, allowing clinicians to stratify patients and prescribe targeted treatments more likely to succeed. This precision reduces trial-and-error approaches and enhances treatment efficiency. Additionally, AI supports microbiome-centered strategies, which are gaining traction in regenerative medicine. By analyzing microbiome composition and its interaction with host physiology, AI can inform therapeutic decisions that align with a patient’s unique microbial environment [253]. his enhances therapeutic durability and patient tolerance while minimizing disruptions to the microbiome.

Remote monitoring technologies, powered by AI, offer further patient-centric advantages. Wearable devices can continuously track health metrics, such as vital signs or biomarkers, and transmit the data to healthcare systems. AI analyzes these inputs in real time, facilitating timely clinical decisions and interventions. This is especially valuable for patients with chronic or mobility-limiting conditions, who benefit from reduced hospital visits and uninterrupted care. Additionally, AI-driven telehealth solutions enhance accessibility for patients in remote or underserved regions, enabling early diagnosis and timely treatment.Finally, AI’s predictive modeling capabilities serve a critical role in preemptive healthcare. By identifying potential complications or side effects before they occur, AI empowers clinicians to implement risk-mitigation strategies. For chronic and progressive illnesses, this anticipatory care model can significantly alter disease trajectories, improving prognosis and long-term outcomes.

## Future perspectives, challenges, and ethical integration of AI-nanomaterial therapies

The convergence of nanotechnology, microbiome science, regenerative medicine, and AI heralds a transformative shift in the treatment of chronic infections and inflammation-linked diseases. As highlighted throughout this review, the development of multifunctional nanomaterials particularly those that are stimuli-responsive, biocompatible, and sensitive to host-microbe interactions offers a strategic avenue to overcome the limitations of traditional antimicrobial and anti-inflammatory therapies. Future directions will focus on precision-engineered platforms that not only eradicate biofilms and drug-resistant pathogens but also facilitate tissue regeneration, microbiome restoration, and real-time therapeutic modulation.

One key frontier involves personalized nanotherapeutics that account for individual microbiome compositions, immune profiles, and wound pathophysiology. These systems will combine engineered nanomaterials with omics-driven patient data to deliver precisely tuned therapies. For example, biomaterials capable of releasing growth factors in synchrony with microbiome recovery stages or anti-inflammatory agents in response to specific immune signatures represent the next generation of biologically intelligent systems. These platforms must be adaptable not only to the biochemical signals of infection and inflammation but also to mechanical, enzymatic, and microbial stressors, particularly in complex wounds, diabetic ulcers, and infection-related cancers.

From an engineering standpoint, microbiome-responsive hydrogels, enzyme-cleavable coatings, and dual-acting nanosystems (e.g., combining photothermal therapy with antibiotic release) require significant refinement for clinical deployment. This involves addressing spatiotemporal control, ensuring precise localization, activation, and degradation of materials in a patient-specific manner. Additionally, novel strategies are needed to prevent premature degradation and minimize cytotoxicity, especially in immunocompromised patients. Hybrid nanomaterials incorporating natural polymers like chitosan or cellulose with metallic cores could offer a sustainable and effective solution, provided challenges in manufacturing consistency and storage stability are addressed.

The role of AI in optimizing nanomaterial design, as discussed in prior sections, is poised to expand further. Beyond passive data analysis, future AI tools will be embedded into closed-loop systems capable of real-time interpretation and actuation, adjusting drug release or material properties based on dynamic physiological feedback. These advances will rely heavily on the integration of organ-on-chip platforms and digital twin models, enabling continuous in vitro and in silico validation of therapeutic decisions before patient application. However, robust modeling of host–microbiome–pathogen interactions and material–tissue dynamics remains a major gap that must be filled through interdisciplinary research.

Another urgent need is dataset standardization and regulatory harmonization. The lack of shared ontologies for describing nanomaterial properties, therapeutic outcomes, and patient response metrics inhibits both reproducibility and algorithm transferability. Global efforts are needed to create open-source, annotated databases capturing microbiome states, infection progression, wound healing stages, and material biocompatibility under various clinical conditions. These repositories will be instrumental for both training AI systems and informing evidence-based regulatory guidelines for adaptive therapies.

Ethical and policy dimensions must evolve in parallel. Adaptive nanotherapies and AI-driven interventions challenge conventional definitions of medical autonomy, informed consent, and clinical oversight. Patients must be informed about how and when algorithms intervene, particularly in therapies that auto-modulate dosage or target dynamically evolving infections. Ethical frameworks should also guide the use of microbiome-modulating agents, ensuring interventions do not compromise long-term host–microbiota balance or inadvertently promote dysbiosis. Furthermore, algorithmic bias, rooted in non-representative datasets or population-specific responses to materials, must be transparently reported and corrected.

While these innovations open exciting frontiers, their true impact will depend on how effectively they transition into clinical practice. Translational success will require (i) rigorous preclinical-to-clinical pipelines that include long-term toxicity and biodistribution studies, (ii) GMP- compliant manufacturing to ensure reproducibility and scalability, (iii) adaptive trial designs capable of evaluating patient-specific nanotherapies, and (iv) clearer regulatory alignment across FDA/EMA to classify and approve multifunctional nanoplatforms. Importantly, integration into existing wound-care workflows will necessitate collaboration among clinicians, material scientists, and regulatory bodies. Establishing this structured pathway will be essential for moving stimuli-responsive nanomaterials from proof-of-concept innovations into standardized, safe, and accessible therapies for chronic wounds and infection-related diseases.

**Future directions: open scientific and clinical questions**

- How can long-term safety, biodistribution, and immunocompatibility of multifunctional nanomaterials be reliably assessed in humans?
- What are the optimal strategies for integrating AI-driven design with patient-specific microbiome and immune profiles?
- How can long-term safety, biodistribution, and immunocompatibility of multifunctional nanomaterials be reliably assessed in humans?
- What regulatory pathways (FDA/EMA) are most appropriate for hybrid nanoplatforms combining antimicrobial, regenerative, and AI-enabled functions?
- Can adaptive nanotherapies be effectively scaled to GMP production without compromising environmental sustainability?
- How should ethical frameworks evolve to address patient consent, algorithmic decision making,and microbiome modulation risks?

Environmental sustainability will define the long-term viability of nanomedicine. Despite the growing interest in green synthesis techniques, current large-scale manufacturing of nanomaterials and embedded electronic components often involves rare earth metals, organic solvents, and non-degradable polymers. Future systems must prioritize eco-conscious alternatives, such as biopolymer matrices, plant-derived reducing agents, and recyclable hardware components. Moreover, frameworks for safe disposal and circular reuse of nanotherapeutic devices must be developed in alignment with life-cycle assessment principles.

## Translational, immunocompatibility, and regulatory considerations

Despite the rapid progress in smart hybrid nanomaterials for chronic infections, their successful clinical translation remains a considerable challenge. Scaling laboratory formulations into clinically viable products requires adherence to Good Manufacturing Practices (GMP) [119], with stringent demands for reproducibility, sterility, and batch-to-batch consistency. Variations in nanomaterial size, surface charge, or functionalization can critically alter pharmacokinetics and therapeutic outcomes, making standardization a pressing bottleneck. Moreover, reliable analytical protocols for quality control and long-term stability remain underdeveloped, delaying entry of promising nanotherapeutics into clinical trials.

On the regulatory side, agencies such as the U.S. Food and Drug Administration (FDA) and the European Medicines Agency (EMA) have begun issuing guidance documents to address the complexities of nanomedicine evaluation. These include recommendations for comprehensive physicochemical characterization, in vivo biodistribution studies, and detailed toxicological profiling. However, regulatory pathways are not yet fully harmonized, and classification ambiguities often exist-whether a product is regulated as a drug, biologic, or combination device. This uncertainty can prolong approval timelines and create additional hurdles for researchers and developers. Equally important are the immunological considerations of nanomaterial-based therapies. NPs may activate the complement cascade, trigger hypersensitivity reactions, or induce off-target inflammatory responses, all of which can compromise patient safety. Recent strategies to improve immunocompatibility include the incorporation of biomimetic coatings, the use of stealth polymers such as PEG and zwitterionic layers, and the design of surface modifications that minimize opsonization. While such approaches are promising, systematic immuno-safety assessment frameworks remain limited, and predictive models for patient-specific immune responses are urgently needed.

Finally, the path to translation also requires coordinated global efforts in regulatory science. Standardized testing workflows, cross-validation of results across laboratories, and international alignment of approval processes are critical to accelerate clinical adoption. Emerging multi-stakeholder consortia that include academia, industry, and regulators are beginning to shape this landscape by sharing data, defining best practices, and establishing reference materials for evaluation. Addressing these translational and regulatory gaps will be essential for moving smart nanomaterials from promising laboratory tools to clinically approved therapies that can transform the management of chronic infections.

## Conclusion

This review emphasises the revolutionary potential of microbiome-responsive smart hybrid nanomaterials in treating chronic infections and fostering tissue regeneration, especially those that incorporate green polymers like chitosan and AI-optimized designs. By utilising non-traditional antimicrobial mechanisms like ROS generation and pH/enzyme-triggered release, these multifunctional platforms not only modulate dysbiosis and disrupt resilient biofilms, but they also achieve up to 90% wound closure in preclinical models. Importantly, a paradigm shift in infection management is being brought about by the combination of AI-driven design, sustainable biomaterials, and microbiome-informed strategies. These systems provide a comprehensive therapeutic model, targeting pathogens, boosting host immunity, and improving regenerative outcomes all at once, in contrast to conventional methods that break down antimicrobial action from tissue repair. By facilitating real-time treatment adaptation, predictive modelling, and metagenomic analysis for personalised microbiome stratification, AI further spurs innovation. Clinical translation in the future will depend on thorough microbiome profiling, life-cycle sustainability evaluations, and progressive regulatory frameworks. If effectively adopted, these multidisciplinary innovations have the potential to reshape the norms for ethically guided, environmentally responsible, and individualised healthcaregreen.

## Data Availability

No datasets were generated or analysed during the current study.

## Abbreviations

AI: Artificial intelligence
CNC: Cellulose nanocrystals
DFU: Diabetic foot ulcer
EPS: Extracellular polymeric substances
GOx: Glucose oxidase
LCA: Life cycle assessment
MMPs: Matrix metalloproteinases
NC: Nanocellulose
PLA: Polylactic acid
ROS: Reactive oxygen species
SCFA: Short-chain fatty acids
TMAO: Trimethylamine N-oxide
ECM: Extracellular matrix
CFU: Colony-forming units

## References

[1] Collaborators GBDCoD. Global burden of 288 causes of death and life expectancy decomposition in 204 countries and territories and 811 subnational locations, 1990–2021: a systematic analysis for the global burden of disease study 2021. Lancet. 2024;403:2100–32. 38582094 10.1016/S0140-6736(24)00367-2 PMC11126520

[2] Barman S, Kurnaz LB, Leighton R, Hossain MW, Decho AW, Tang C. Intrinsic antimicrobial resistance: molecular biomaterials to combat microbial biofilms and bacterial persisters. Biomaterials. 2024;311:122690. 38976935 10.1016/j.biomaterials.2024.122690 PMC11298303

[3] Antimicrobial Resistance C. The burden of bacterial antimicrobial resistance in the WHO African region in 2019: a cross-country systematic analysis. Lancet Glob Health. 2024;12:e201–16. 38134946 10.1016/S2214-109X(23)00539-9 PMC10805005

[4] Uberoi A, McCready-Vangi A, Grice EA. The wound microbiota: microbial mechanisms of impaired wound healing and infection. Nat Rev Microbiol. 2024;22:507–21. 38575708 10.1038/s41579-024-01035-z

[5] Monack DM, Mueller A, Falkow S. Persistent bacterial infections: the interface of the pathogen and the host immune system. Nat Rev Microbiol. 2004;2:747–65. 15372085 10.1038/nrmicro955

[6] Filloux A, Davies JC. Chronic infection by controlling inflammation. Nat Microbiol. 2019;4:378–9. 30787479 10.1038/s41564-019-0397-6

[7] Bhattarai SK, Du M, Zeamer AL, Kellogg BMM, Firat TD. Commensal antimicrobial resistance mediates microbiome resilience to antibiotic disruption. Sci Transl Med. 2024;16:eadi9711. 38232140 10.1126/scitranslmed.adi9711 PMC11017772

[8] Engelsberger V, Gerhard M, Mejias-Luque R. Effects of *Helicobacter pylori* infection on intestinal microbiota, immunity and colorectal cancer risk. Front Cell Infect Microbiol. 2024;14:1339750. 38343887 10.3389/fcimb.2024.1339750 PMC10853882

[9] Tavakoli S, Klar AS. Advanced hydrogels as wound dressings. Biomolecules. 2020;10(8):1169. 10.3390/biom10081169. 32796593 10.3390/biom10081169 PMC7464761

[10] Singh P, Kim YJ, Zhang D, Yang DC. Biological synthesis of nanoparticles from plants and microorganisms. Trends Biotechnol. 2016;34:588–99. 26944794 10.1016/j.tibtech.2016.02.006

[11] Singh P, Kim YJ, Wang C, Mathiyalagan R, Yang DC. The development of a green approach for the biosynthesis of silver and gold nanoparticles by using *Panax ginseng* root extract, and their biological applications. Artif Cells Nanomed Biotechnol. 2016;44:1150–7. 25771716 10.3109/21691401.2015.1011809

[12] Jeyaraman M, Ratna HVK, Jeyaraman N, Venkatesan A, Ramasubramanian S, Yadav S. Leveraging artificial intelligence and machine learning in regenerative orthopedics: a paradigm shift in patient care. Cureus. 2023;15:e49756. 38161806 10.7759/cureus.49756 PMC10757680

[13] Asadi Sarabi P, Shabanpouremam M, Eghtedari AR, Barat M, Moshiri B, Zarrabi A, et al. Ai-based solutions for current challenges in regenerative medicine. Eur J Pharmacol. 2024;984:177067. 39454850 10.1016/j.ejphar.2024.177067

[14] Gharibshahian M, Torkashvand M, Bavisi M, Aldaghi N, Alizadeh A. Recent advances in artificial intelligent strategies for tissue engineering and regenerative medicine. Skin Res Technol. 2024;30:e70016. 39189880 10.1111/srt.70016 PMC11348508

[15] Nosrati H, Nosrati M. Artificial intelligence in regenerative medicine: applications and implications. Biomimetics. 2023;8(5):442. 10.3390/biomimetics8050442. 37754193 10.3390/biomimetics8050442 PMC10526210

[16] Akkus G, Sert M. Diabetic foot ulcers: a devastating complication of diabetes mellitus continues non-stop in spite of new medical treatment modalities. World J Diabetes. 2022;13:1106–21. 36578865 10.4239/wjd.v13.i12.1106 PMC9791571

[17] Larouche J, Sheoran S, Maruyama K, Martino MM. Immune regulation of skin wound healing: mechanisms and novel therapeutic targets. Adv Wound Care. 2018;7:209–31. 10.1089/wound.2017.0761 PMC6032665 29984112

[18] DeGruttola AK, Low D, Mizoguchi A, Mizoguchi E. Current understanding of dysbiosis in disease in human and animal models. Inflamm Bowel Dis. 2016;22:1137–50. 27070911 10.1097/MIB.0000000000000750 PMC4838534

[19] Gadani SP, Walsh JT, Lukens JR, Kipnis J. Dealing with danger in the CNS: the response of the immune system to injury. Neuron. 2015;87:47–62. 26139369 10.1016/j.neuron.2015.05.019 PMC4491143

[20] Cangui-Panchi SP, Nacato-Toapanta AL, Enriquez-Martinez LJ, Salinas-Delgado GA, Reyes J, Garzon-Chavez D, et al. Battle royale: immune response on biofilms - host-pathogen interactions. Curr Res Immunol. 2023;4:100057. 37025390 10.1016/j.crimmu.2023.100057 PMC10070391

[21] Rimi SS, Ashraf MN, Sigma SH, Ahammed MT, Siddique MP, Zinnah MA, et al. Biofilm formation, Agr typing and antibiotic resistance pattern in methicillin-resistant *Staphylococcus aureus* isolated from hospital environments. PLoS ONE. 2024;19:e0308282. 39102390 10.1371/journal.pone.0308282 PMC11299820

[22] Gholami A, Minai-Tehrani D, Mahdizadeh SJ, Saenz-Mendez P, Eriksson LA. Structural insights into *Pseudomonas aeruginosa* exotoxin A-elongation factor 2 interactions: a molecular dynamics study. J Chem Inf Model. 2023;63:1578–91. 36802593 10.1021/acs.jcim.3c00064 PMC10015456

[23] Murru A, Sommerhoff C. First, thou shall not chronicize: the risk of untreated insomnia. Eur Neuropsychopharmacol. 2024;83:56. 38643637 10.1016/j.euroneuro.2024.03.009

[24] Berbudi A, Rahmadika N, Tjahjadi AI, Ruslami R. Type 2 diabetes and its impact on the immune system. Curr Diabetes Rev. 2020;16:442–9. 31657690 10.2174/1573399815666191024085838 PMC7475801

[25] Vinaik R, Abdullahi A, Barayan D, Jeschke MG. Nlrp3 inflammasome activity is required for wound healing after burns. Transl Res. 2020;217:47–60. 31843468 10.1016/j.trsl.2019.11.002 PMC7036017

[26] Solari JIG, Filippi-Chiela E, Pilar ES, Nunes V, Gonzalez EA, Figueiro F, et al. Damage-associated molecular patterns (DAMPs) related to immunogenic cell death are differentially triggered by clinically relevant chemotherapeutics in lung adenocarcinoma cells. BMC Cancer. 2020;20:474. 32456685 10.1186/s12885-020-06964-5 PMC7251700

[27] Braga TT, Forni MF, Correa-Costa M, Ramos RN, Barbuto JA, Branco P, et al. Soluble uric acid activates the NLRP3 inflammasome. Sci Rep. 2017;7:39884. 28084303 10.1038/srep39884 PMC5233987

[28] Chen R, Zou J, Chen J, Zhong X, Kang R, Tang D. Pattern recognition receptors: function, regulation and therapeutic potential. Signal Transduct Target Ther. 2025;10:216. 40640149 10.1038/s41392-025-02264-1 PMC12246121

[29] Wang L, Hauenstein AV. The NLRP3 inflammasome: mechanism of action, role in disease and therapies. Mol Aspects Med. 2020;76:100889. 32859386 10.1016/j.mam.2020.100889

[30] Gorfu G, Cirelli KM, Melo MB, Mayer-Barber K, Crown D, Koller BH, et al. Dual role for inflammasome sensors NLRP1 and NLRP3 in murine resistance to *Toxoplasma gondii*. mBio. 2014;5(1):e01117-13. 10.1128/mBio.01117-13. 24549849 10.1128/mBio.01117-13 PMC3944820

[31] Yuan X, Wang L, Bhat OM, Lohner H, Li PL. Differential effects of short chain fatty acids on endothelial Nlrp3 inflammasome activation and neointima formation: antioxidant action of butyrate. Redox Biol. 2018;16:21–31. 29475132 10.1016/j.redox.2018.02.007 PMC5842312

[32] Chen L, DiPietro LA. Toll-like receptor function in acute wounds. Adv Wound Care. 2017;6:344–55. 10.1089/wound.2017.0734 PMC5649397 29062591

[33] Zhu J, Mohan C. Toll-like receptor signaling pathways–therapeutic opportunities. Mediators Inflamm. 2010;2010:781235. 20981241 10.1155/2010/781235 PMC2963142

[34] Suga H, Sugaya M, Fujita H, Asano Y, Tada Y, Kadono T, et al. TLR4, rather than TLR2, regulates wound healing through TGF-beta and CCL5 expression. J Dermatol Sci. 2014;73:117–24. 24252748 10.1016/j.jdermsci.2013.10.009

[35] Dasu MR, Martin SJ. Toll-like receptor expression and signaling in human diabetic wounds. World J Diabetes. 2014;5:219–23. 24748934 10.4239/wjd.v5.i2.219 PMC3990321

[36] Carneiro PP, Dorea AS, Oliveira WN, Guimaraes LH, Brodskyn C, Carvalho EM, et al. Blockade of TLR2 and TLR4 attenuates inflammatory response and parasite load in cutaneous leishmaniasis. Front Immunol. 2021;12:706510. 34691019 10.3389/fimmu.2021.706510 PMC8526941

[37] Zheng D, Liwinski T, Elinav E. Interaction between microbiota and immunity in health and disease. Cell Res. 2020;30:492–506. 32433595 10.1038/s41422-020-0332-7 PMC7264227

[38] Phillipson M, Kubes P. The healing power of neutrophils. Trends Immunol. 2019;40:635–47. 31160208 10.1016/j.it.2019.05.001

[39] Herb M, Schramm M. Functions of ROS in macrophages and antimicrobial immunity. Antioxidants. 2021;10(2):313. 10.3390/antiox10020313. 33669824 10.3390/antiox10020313 PMC7923022

[40] Zhang M, Guo B, Zhang X, Han D, Lv L, Yan X, et al. IFP35, a novel DAMP, aggravates neuroinflammation following acute ischemic stroke via TLR4/NF-kappaB/NLRP3 signaling. J Neuroinflammation. 2025;22:164. 40563086 10.1186/s12974-025-03492-6 PMC12188676

[41] Schleicher U, Paduch K, Debus A, Obermeyer S, Konig T, Kling JC, et al. Tnf-mediated restriction of arginase 1 expression in myeloid cells triggers type 2 NO synthase activity at the site of infection. Cell Rep. 2016;15:1062–75. 27117406 10.1016/j.celrep.2016.04.001 PMC5065922

[42] Mirza R, Koh TJ. Dysregulation of monocyte/macrophage phenotype in wounds of diabetic mice. Cytokine. 2011;56:256–64. 21803601 10.1016/j.cyto.2011.06.016

[43] Seilie ES, Bubeck Wardenburg J. *Staphylococcus aureus* pore-forming toxins: the interface of pathogen and host complexity. Semin Cell Dev Biol. 2017;72:101–16. 28445785 10.1016/j.semcdb.2017.04.003 PMC5823538

[44] Kiran NS, Chatterjee A, Yashaswini C, Deshmukh R, Alsaidan OA, Bhattacharya S, et al. The gastrointestinal mycobiome in inflammation and cancer: unraveling fungal dysbiosis, pathogenesis, and therapeutic potential. Med Oncol. 2025;42:195. 40323477 10.1007/s12032-025-02761-x

[45] Schilrreff P, Alexiev U. Chronic inflammation in non-healing skin wounds and promising natural bioactive compounds treatment. Int J Mol Sci. 2022;23(9):4928. 10.3390/ijms23094928. 35563319 10.3390/ijms23094928 PMC9104327

[46] Geraci J, Neubauer S, Pollath C, Hansen U, Rizzo F, Krafft C, et al. The *Staphylococcus aureus* extracellular matrix protein (Emp) has a fibrous structure and binds to different extracellular matrices. Sci Rep. 2017;7:13665. 29057978 10.1038/s41598-017-14168-4 PMC5651841

[47] Zhu Z, Hu Z, Li S, Fang R, Ono HK, Hu DL. Molecular characteristics and pathogenicity of *Staphylococcus aureus* exotoxins. Int J Mol Sci. 2023;25(1):395. 10.3390/ijms25010395. 38203566 10.3390/ijms25010395 PMC10778951

[48] Howden BP, Giulieri SG, Wong Fok Lung T, Baines SL, Sharkey LK, Lee JYH, et al. *Staphylococcus aureus* host interactions and adaptation. Nat Rev Microbiol. 2023;21:380–95. 36707725 10.1038/s41579-023-00852-y PMC9882747

[49] da Cruz Nizer WS, Inkovskiy V, Versey Z, Strempel N, Cassol E, Overhage J. Oxidative stress response in *Pseudomonas aeruginosa*. Pathogens. 2021;10(9):1187. 10.3390/pathogens10091187. 34578219 10.3390/pathogens10091187 PMC8466533

[50] Liao C, Huang X, Wang Q, Yao D, Lu W. Virulence factors of *Pseudomonas aeruginosa* and antivirulence strategies to combat its drug resistance. Front Cell Infect Microbiol. 2022;12:926758. 35873152 10.3389/fcimb.2022.926758 PMC9299443

[51] Wang S, Xiang D, Tian F, Ni M. Lipopolysaccharide from biofilm-forming *Pseudomonas aeruginosa* PAO1 induces macrophage hyperinflammatory responses. J Med Microbiol. 2021;70(4):001352. 10.1099/jmm.0.001352. 33909550 10.1099/jmm.0.001352 PMC8289208

[52] Sierig G, Cywes C, Wessels MR, Ashbaugh CD. Cytotoxic effects of streptolysin o and streptolysin s enhance the virulence of poorly encapsulated group a streptococci. Infect Immun. 2003;71:446–55. 12496195 10.1128/IAI.71.1.446-455.2003 PMC143243

[53] Shumba P, Mairpady Shambat S, Siemens N. The role of Streptococcal and Staphylococcal exotoxins and proteases in human necrotizing soft tissue infections. Toxins (Basel). 2019;11(6):332. 10.3390/toxins11060332. 31212697 10.3390/toxins11060332 PMC6628391

[54] Yuen GJ, Ausubel FM. Enterococcus infection biology: lessons from invertebrate host models. J Microbiol. 2014;52:200–10. 24585051 10.1007/s12275-014-4011-6 PMC4556283

[55] Schulz E, Schumann M, Schneemann M, Dony V, Fromm A, Nagel O, et al. *Escherichia coli* alpha-hemolysin HlyA induces host cell polarity changes, epithelial barrier dysfunction and cell detachment in human colon carcinoma Caco-2 cell model via PTEN-dependent dysregulation of cell junctions. Toxins (Basel). 2021;13(8):520. 10.3390/toxins13080520. 34437391 10.3390/toxins13080520 PMC8402498

[56] Page MJ, Kell DB, Pretorius E. The role of lipopolysaccharide-induced cell signalling in chronic inflammation. Chronic Stress. 2022;6:24705470221076390. 35155966 10.1177/24705470221076390 PMC8829728

[57] Urbina P, Collado MI, Alonso A, Goni FM, Flores-Diaz M, Alape-Giron A, et al. Unexpected wide substrate specificity of C. perfringens alpha-toxin phospholipase C. Biochim Biophys Acta. 2011;1808:2618–27. 21704605 10.1016/j.bbamem.2011.06.008

[58] Ellemor DM, Baird RN, Awad MM, Boyd RL, Rood JI, Emmins JJ. Use of genetically manipulated strains of *Clostridium perfringens* reveals that both alpha-toxin and theta-toxin are required for vascular leukostasis to occur in experimental gas gangrene. Infect Immun. 1999;67:4902–7. 10456947 10.1128/iai.67.9.4902-4907.1999 PMC96825

[59] Opoku-Temeng C, Kobayashi SD, DeLeo FR. *Klebsiella pneumoniae* capsule polysaccharide as a target for therapeutics and vaccines. Comput Struct Biotechnol J. 2019;17:1360–6. 31762959 10.1016/j.csbj.2019.09.011 PMC6861629

[60] Armbruster CE, Mobley HLT, Pearson MM. Pathogenesis of *Proteus mirabilis* infection. EcoSal Plus. 2018(1). 10.1128/ecosalplus.esp-0009-2017. 10.1128/ecosalplus.esp-0009-2017 PMC5880328 29424333

[61] Wexler HM. Bacteroides: the good, the bad, and the nitty-gritty. Clin Microbiol Rev. 2007;20:593–621. 17934076 10.1128/CMR.00008-07 PMC2176045

[62] Jun SH, Lee JH, Kim BR, Kim SI, Park TI, Lee JC, et al. *Acinetobacter baumannii* outer membrane vesicles elicit a potent innate immune response via membrane proteins. PLoS ONE. 2013;8:e71751. 23977136 10.1371/journal.pone.0071751 PMC3743744

[63] Kyriakidis I, Vasileiou E, Pana ZD, Tragiannidis A. *Acinetobacter baumannii* antibiotic resistance mechanisms. Pathogens. 2021;10(3):373. 10.3390/pathogens10030373. 33808905 10.3390/pathogens10030373 PMC8003822

[64] Xiao J, Jiang J, He X, Zhang S, Wang Z, Wang F, et al. Evaluation of immunoprotective effects of *Fusobacterium necrophorum* outer membrane proteins 43K OMP, leukotoxin and hemolysin multi-component recombinant subunit vaccine in mice. Front Vet Sci. 2021;8:780377. 34938794 10.3389/fvets.2021.780377 PMC8685265

[65] Larsen JM. The immune response to prevotella bacteria in chronic inflammatory disease. Immunology. 2017;151:363–74. 28542929 10.1111/imm.12760 PMC5506432

[66] DuMont AL, Cianciotto NP. *Stenotrophomonas maltophilia* serine protease StmPr1 induces matrilysis, anoikis, and protease-activated receptor 2 activation in human lung epithelial cells. Infect Immun. 2017;85(12):e00544-17. 10.1128/IAI.00544-17. 28893914 10.1128/IAI.00544-17 PMC5695115

[67] Hansen P, Haubenthal T, Reiter C, Kniewel J, Bosse-Plois K, Niemann HH, et al. Differential effects of Rhodococcus equi Virulence-Associated proteins on macrophages and artificial lipid membranes. Microbiol Spectr. 2023;11:e0341722. 36786596 10.1128/spectrum.03417-22 PMC10100859

[68] Yuan Y, Feng Z, Wang J. *Vibrio vulnificus* hemolysin: biological activity, regulation of VvhA expression, and role in pathogenesis. Front Immunol. 2020;11:599439. 33193453 10.3389/fimmu.2020.599439 PMC7644469

[69] Rodriguez JM, Murphy K, Stanton C, Ross RP, Kober OI, Juge N, et al. The composition of the gut microbiota throughout life, with an emphasis on early life. Microb Ecol Health Dis. 2015;26(0):26050. 25651996 10.3402/mehd.v26.26050 PMC4315782

[70] Mitchell MJ, Billingsley MM, Haley RM, Wechsler ME, Peppas NA, Langer R. Engineering precision nanoparticles for drug delivery. Nat Reviews Drug Discovery 2020. 2020;20:2. 10.1038/s41573-020-0090-8 PMC7717100 33277608

[71] Singh H, Du J, Singh P, Yi TH. Ecofriendly synthesis of silver and gold nanoparticles by *Euphrasia officinalis* leaf extract and its biomedical applications. Artif Cells Nanomed Biotechnol. 2018;46:1163–70. 28784039 10.1080/21691401.2017.1362417

[72] Jhonsi MA, Ananth DA, Nambirajan G, Sivasudha T, Yamini R, Bera S, et al. Antimicrobial activity, cytotoxicity and DNA binding studies of carbon dots. Spectrochim Acta A Mol Biomol Spectrosc. 2018;196:295–302. 29459160 10.1016/j.saa.2018.02.030

[73] Meziani MJ, Dong X, Zhu L, Jones LP, LeCroy GE, Yang F, et al. Visible-light-activated bactericidal functions of carbon quantum dots. ACS Appl Mater Interfaces. 2016;8:10761–6. 27064729 10.1021/acsami.6b01765 PMC5017886

[74] Khalid A, Madni A, Raza B, Islam MU, Hassan A, Ahmad F, et al. Multiwalled carbon nanotubes functionalized bacterial cellulose as an efficient healing material for diabetic wounds. Int J Biol Macromol. 2022;203:256–67. 35093443 10.1016/j.ijbiomac.2022.01.146

[75] Nandhakumar M, Thangaian DT, Sundaram S, Roy A, Subramanian B. An enduring in vitro wound healing phase recipient by bioactive glass-graphene oxide nanocomposites. Sci Rep. 2022;12:16162. 36171341 10.1038/s41598-022-20575-z PMC9519557

[76] Sharma S, Bose A, Biswas S, Sen S, Roy I. *Cyperus rotundus* mediated green synthesis of silver nanoparticles for antibacterial wound dressing applications. Sci Rep. 2025;15:18394. 40419643 10.1038/s41598-025-03555-x PMC12106647

[77] Alhosani F, Islayem D, Almansoori S, Zaka A, Nayfeh L, Rezk A, et al. Antibiofilm activity of ZnO-Ag nanoparticles against *Pseudomonas aeruginosa*. Sci Rep. 2025;15:17321. 40389571 10.1038/s41598-025-02372-6 PMC12089420

[78] Mathesh A, Mohanprasanth A, Saravanan M. Synthesis and characterization of Spirulina-mediated titanium dioxide nanoparticles: antimicrobial activity against multidrug-resistant bacteria. Nano-Structures Nano-Objects. 2024;39:101225.

[79] Yu W, Tang J, Gao C, Zheng X, Zhu P. Green synthesis of copper nanoparticles from the aqueous extract of *lonicera japonica* Thunb and evaluation of its catalytic property and cytotoxicity and antimicrobial activity. Nanomaterials. 2025;15(2):91. 10.3390/nano15020091. 39852706 10.3390/nano15020091 PMC11767692

[80] Sklodowski K, Chmielewska-Deptula SJ, Piktel E, Wolak P, Wollny T, Bucki R. Metallic nanosystems in the development of antimicrobial strategies with high antimicrobial activity and high biocompatibility. Int J Mol Sci. 2023;24(3):2104. 10.3390/ijms24032104 10.3390/ijms24032104 PMC9917064 36768426

[81] Wathoni N, Herdiana Y, Suhandi C, Mohammed AFA, El-Rayyes A, Narsa AC. Chitosan/alginate-based nanoparticles for antibacterial agents delivery. Int J Nanomed. 2024;19:5021–44. 10.2147/IJN.S469572 PMC11146614 38832335

[82] Szymczak M, Pankowski JA, Kwiatek A, Grygorcewicz B, Karczewska-Golec J, Sadowska K, et al. An effective antibiofilm strategy based on bacteriophages armed with silver nanoparticles. Sci Rep. 2024;14:9088. 38643290 10.1038/s41598-024-59866-y PMC11032367

[83] Blanco Massani M, To D, Meile S, Schmelcher M, Gintsburg D, Coraca-Huber DC, et al. Enzyme-responsive nanoparticles: enhancing the ability of endolysins to eradicate *Staphylococcus aureus* biofilm. J Mater Chem B. 2024;12:9199–205. 39263769 10.1039/d4tb01122h

[84] Hajipour MJ, Saei AA, Walker ED, Conley B, Omidi Y, Lee KB, et al. Nanotechnology for targeted detection and removal of bacteria: opportunities and challenges. Adv Sci (Weinh). 2021;8:e2100556. 34558234 10.1002/advs.202100556 PMC8564466

[85] Garcia DG, Garzon-Romero C, Salazar MA, Lagos KJ, Campana KO, Debut A, et al. Bioinspired synthesis of magnetic nanoparticles based on iron oxides using orange waste and their application as photo-activated antibacterial agents. Int J Mol Sci. 2023;24(5):4770. 10.3390/ijms24054770. 36902198 10.3390/ijms24054770 PMC10002579

[86] Zharkova MS, Golubeva OY, Orlov DS, Vladimirova EV, Dmitriev AV, Tossi A, et al. Silver nanoparticles functionalized with antimicrobial polypeptides: benefits and possible pitfalls of a novel anti-infective tool. Front Microbiol. 2021;12:750556. 34975782 10.3389/fmicb.2021.750556 PMC8719061

[87] Kao WK, Gagnon PM, Vogel JP, Chole RA. Surface charge modification decreases *Pseudomonas aeruginosa* adherence in vitro and bacterial persistence in an in vivo implant model. Laryngoscope. 2017;127:1655–61. 28295372 10.1002/lary.26499 PMC5476480

[88] Miranda Calderon LG, Alejo T, Santos S, Mendoza G, Irusta S, Arruebo M. Antibody-functionalized polymer nanoparticles for targeted antibiotic delivery in models of pathogenic bacteria infecting human macrophages. ACS Appl Mater Interfaces. 2023;15:40213–27. 37596966 10.1021/acsami.3c07367 PMC10877563

[89] Zhang J, Tang W, Zhang X, Song Z, Tong T. An overview of stimuli-responsive intelligent antibacterial nanomaterials. Pharmaceutics. 2023;15(8):2113. 10.3390/pharmaceutics15082113. 37631327 10.3390/pharmaceutics15082113 PMC10458108

[90] Li LL, Xu JH, Qi GB, Zhao X, Yu F, Wang H. Core-shell supramolecular gelatin nanoparticles for adaptive and on-demand antibiotic delivery. ACS Nano. 2014;8:4975–83. 24716550 10.1021/nn501040h

[91] Landis RF, Li CH, Gupta A, Lee YW, Yazdani M, Ngernyuang N, et al. Biodegradable nanocomposite antimicrobials for the eradication of multidrug-resistant bacterial biofilms without accumulated resistance. J Am Chem Soc. 2018;140:6176–82. 29709168 10.1021/jacs.8b03575 PMC6044909

[92] Rogovski P, Cadamuro RD, da Silva R, de Souza EB, Bonatto C, Viancelli A, et al. Uses of bacteriophages as bacterial control tools and environmental safety indicators. Front Microbiol. 2021;12:793135. 34917066 10.3389/fmicb.2021.793135 PMC8670004

[93] Kim MK, Chen Q, Echterhof A, Pennetzdorfer N, McBride RC, Banaei N, et al. A blueprint for broadly effective bacteriophage-antibiotic cocktails against bacterial infections. Nat Commun. 2024;15:9987. 39609398 10.1038/s41467-024-53994-9 PMC11604943

[94] Kunisch F, Campobasso C, Wagemans J, Yildirim S, Chan BK, Schaudinn C, et al. Targeting *Pseudomonas aeruginosa* biofilm with an evolutionary trained bacteriophage cocktail exploiting phage resistance trade-offs. Nat Commun. 2024;15:8572. 39362854 10.1038/s41467-024-52595-w PMC11450229

[95] Lu Y, Wang Y, Wang J, Zhao Y, Zhong Q, Li G, et al. Phage endolysin LysP108 showed promising antibacterial potential against Methicillin-resistant *Staphylococcus aureus*. Front Cell Infect Microbiol. 2021;11:668430. 33937105 10.3389/fcimb.2021.668430 PMC8082462

[96] Juarez VM, Montalbine AN, Singh A. Microbiome as an immune regulator in health, disease, and therapeutics. Adv Drug Deliv Rev. 2022;188:114400. 35718251 10.1016/j.addr.2022.114400 PMC10751508

[97] Vetizou M, Pitt JM, Daillere R, Lepage P, Waldschmitt N, Flament C, et al. Anticancer immunotherapy by CTLA-4 blockade relies on the gut microbiota. Science. 2015;350:1079–84. 26541610 10.1126/science.aad1329 PMC4721659

[98] Mager LF, Burkhard R, Pett N, Cooke NCA, Brown K, Ramay H, et al. Microbiome-derived inosine modulates response to checkpoint inhibitor immunotherapy. Science. 2020;369:1481–9. 32792462 10.1126/science.abc3421

[99] Shi Y, Zheng W, Yang K, Harris KG, Ni K, Xue L, et al. Intratumoral accumulation of gut microbiota facilitates CD47-based immunotherapy via STING signaling. J Exp Med. 2020;217(5):e20192282. 10.1084/jem.20192282. 32142585 10.1084/jem.20192282 PMC7201921

[100] Zhao J, Wang Y, Wang J, Lv M, Zhou C, Jia L, et al. *Lactobacillus kefiranofaciens* ZW18 from kefir enhances the anti-tumor effect of anti-programmed cell death 1 (PD-1) immunotherapy by modulating the gut microbiota. Food Funct. 2022;13:10023–33. 36069328 10.1039/d2fo01747d

[101] Zhang S-L, Han B, Mao Y-Q, Zhang Z-Y, Li Z-M, Kong C-Y, et al. *Lacticaseibacillus paracasei* sh2020 induced antitumor immunity and synergized with anti-programmed cell death 1 to reduce tumor burden in mice. Gut Microbes. 2022;14:2046246. 35259052 10.1080/19490976.2022.2046246 PMC8920197

[102] Kang X, Liu C, Ding Y, Ni Y, Ji F, Lau HCH, et al. *Roseburia intestinalis*generated butyrate boosts anti-PD-1 efficacy in colorectal cancer by activating cytotoxic CD8^+^ T cells. Gut. 2023;72(11):2112. 37491158 10.1136/gutjnl-2023-330291 PMC10579466

[103] Montalban-Arques A, Katkeviciute E, Busenhart P, Bircher A, Wirbel J, Zeller G, et al. Commensal clostridiales strains mediate effective anti-cancer immune response against solid tumors. Cell Host Microbe. 2021;29:1573–88. e7. 34453895 10.1016/j.chom.2021.08.001

[104] Fong W, Li Q, Ji F, Liang W, Lau HCH, Kang X, et al. *Lactobacillus gallinarum*-derived metabolites boost anti-PD1 efficacy in colorectal cancer by inhibiting regulatory T cells through modulating IDO1/Kyn/AHR axis. Gut. 2023;72:2272. 37770127 10.1136/gutjnl-2023-329543 PMC10715476

[105] Kang X, Lau HC-H, Yu J. Modulating gut Microbiome in cancer immunotherapy: Harnessing microbes to enhance treatment efficacy. Cell Rep Med. 2024;5(4):101478. 10.1016/j.xcrm.2024.101478. 10.1016/j.xcrm.2024.101478 PMC11031381 38631285

[106] Magalhães-Ghiotto GAV, Oliveira AMd, Natal JPS, Bergamasco R, Gomes RG. Green nanoparticles in water treatment: a review of research trends, applications, environmental aspects and large-scale production. Environ Nanotechnol Monit Manag. 2021;16:100526.

[107] Makabenta JMV, Nabawy A, Li C-H, Schmidt-Malan S, Patel R, Rotello VM. Nanomaterial-based therapeutics for antibiotic-resistant bacterial infections. Nat Rev Microbiol. 2021;19:23–36. 32814862 10.1038/s41579-020-0420-1 PMC8559572

[108] Ahmadi F, Oveisi Z, Samani SM, Amoozgar Z. Chitosan based hydrogels: characteristics and pharmaceutical applications. Res Pharm Sci. 2015;10:1–16. 26430453 PMC4578208

[109] Yang C, Zhu Y, Tian Z, Zhang C, Han X, Jiang S, et al. Preparation of nanocellulose and its applications in wound dressing: a review. Int J Biol Macromol. 2024;254:127997. 37949262 10.1016/j.ijbiomac.2023.127997

[110] Ahmed SF, Mofijur M, Rafa N, Chowdhury AT, Chowdhury S, Nahrin M, et al. Green approaches in synthesising nanomaterials for environmental nanobioremediation: technological advancements, applications, benefits and challenges. Environ Res. 2022;204:111967. 34450159 10.1016/j.envres.2021.111967

[111] Singh P, Pandit S, Balusamy SR, Madhusudanan M, Singh H, Amsath Haseef HM, et al. Advanced nanomaterials for cancer therapy: gold, silver, and iron oxide nanoparticles in oncological applications. Adv Healthc Mater. 2025;14:2403059. 39501968 10.1002/adhm.202403059 PMC11804848

[112] Singh P, Pandit S, Garnaes J, Tunjic S, Mokkapati VR, Sultan A, et al. Green synthesis of gold and silver nanoparticles from *cannabis sativa* (industrial hemp) and their capacity for biofilm inhibition. Int J Nanomedicine. 2018;13:3571–91. 29950836 10.2147/IJN.S157958 PMC6016601

[113] Kaur M, Sood A, Gupta R. Chapter 16 - Biobased materials in drug delivery. In: Ahmed S, Annu, editors. Advanced applications of biobased materials. Elsevier; 2023. pp. 409–45.

[114] Singh P, Pandit S, Mokkapati V, Garg A, Ravikumar V, Mijakovic I. Gold nanoparticles in diagnostics and therapeutics for human cancer. Int J Mol Sci. 2018;19(7):1979. 10.3390/ijms19071979. 29986450 10.3390/ijms19071979 PMC6073740

[115] Ma X, Tian Y, Yang R, Wang H, Allahou LW, Chang J, et al. Nanotechnology in healthcare, and its safety and environmental risks. J Nanobiotechnol. 2024;22:715. 10.1186/s12951-024-02901-x PMC11566612 39548502

[116] Kumah EA, Fopa RD, Harati S, Boadu P, Zohoori FV, Pak T. Human and environmental impacts of nanoparticles: a scoping review of the current literature. BMC Public Health. 2023;23:1059. 37268899 10.1186/s12889-023-15958-4 PMC10239112

[117] Yonathan K, Mann R, Mahbub KR, Gunawan C. The impact of silver nanoparticles on microbial communities and antibiotic resistance determinants in the environment. Environ Pollut. 2022;293:118506. 34793904 10.1016/j.envpol.2021.118506

[118] Gonzalez-Vega JG, Garcia-Ramos JC, Chavez-Santoscoy RA, Castillo-Quinones JE, Arellano-Garcia ME, Toledano-Magana Y. Lung models to evaluate silver nanoparticles’ toxicity and their impact on human health. Nanomaterials. 2022. 10.3390/nano12132316. 35808152 10.3390/nano12132316 PMC9268743

[119] Kus-Liskiewicz M, Fickers P, Ben Tahar I. Biocompatibility and cytotoxicity of gold nanoparticles: recent advances in methodologies and regulations. Int J Mol Sci. 2021;22(20):10952. 10.3390/ijms222010952. 10.3390/ijms222010952 PMC8536023 34681612

[120] Sagadevan MM, Fatimah SEB, Lett I, Moharana JA. Enhancing biocompatibility and functionality: carbon nanotube-polymer nanocomposites for improved biomedical applications. J Drug Deliv Sci Technol. 2024;99:105958.

[121] Singh G, Thakur N, Kumar R. Nanoparticles in drinking water: assessing health risks and regulatory challenges. Sci Total Environ. 2024;949:174940. 39047836 10.1016/j.scitotenv.2024.174940

[122] Wahab A, Muhammad M, Ullah S, Abdi G, Shah GM, Zaman W, et al. Agriculture and environmental management through nanotechnology: eco-friendly nanomaterial synthesis for soil-plant systems, food safety, and sustainability. Sci Total Environ. 2024;926:171862. 38527538 10.1016/j.scitotenv.2024.171862

[123] Mandal AH, Ghosh S, Adhurjya D, Chatterjee P, Samajdar I, Mukherjee D, et al. Exploring the impact of zinc oxide nanoparticles on fish and fish-food organisms: a review. Aquac Rep. 2024;36:102038.

[124] Verma SK, Panda PK, Jha E, Suar M, Parashar SKS. Altered physiochemical properties in industrially synthesized ZnO nanoparticles regulate oxidative stress; induce in vivo cytotoxicity in embryonic zebrafish by apoptosis. Sci Rep. 2017;7:13909. 29066782 10.1038/s41598-017-14039-y PMC5655687

[125] Alaraby M, Abass D, Gutierrez J, Velazquez A, Hernandez A, Marcos R. Reproductive toxicity of nanomaterials using silver nanoparticles and drosophila as models. Molecules. 2024. 10.3390/molecules29235802. 39683959 10.3390/molecules29235802 PMC11643907

[126] Schlich K, Terytze K, Hund-Rinke K. Effect of TiO2 nanoparticles in the earthworm reproduction test. Environ Sci Eur. 2012;24:5. 10.1002/etc.2030 23059754

[127] Dash SR, Kundu CN. Promising opportunities and potential risk of nanoparticle on the society. IET Nanobiotechnol. 2020;14:253–60. 32463015 10.1049/iet-nbt.2019.0303 PMC8676294

[128] Demelash Abera B, Alefe Adimas M. Health benefits and health risks of contaminated fish consumption: current research outputs, research approaches, and perspectives. Heliyon. 2024;10:e33905. 39050454 10.1016/j.heliyon.2024.e33905 PMC11268356

[129] Panzarini E, Mariano S, Carata E, Mura F, Rossi M, Dini L. Intracellular transport of silver and gold nanoparticles and biological responses: an update. Int J Mol Sci. 2018;19. 10.3390/ijms19051305. 29702561 10.3390/ijms19051305 PMC5983807

[130] Saraswat P, Singh S, Prasad M, Misra R, Rajput VD, Ranjan R. Applications of bio-based nanomaterials in environment and agriculture: a review on recent progresses. Hybrid Advances. 2023;4:100097.

[131] Bashir SM, Ahmed Rather G, Patrício A, Haq Z, Sheikh AA, Shah MZH, et al. Chitosan nanoparticles: A versatile platform for biomedical applications. Materials. 2022;15:6521. 36233864 10.3390/ma15196521 PMC9570720

[132] Xing Y, Wang X, Guo X, Yang P, Yu J, Shui Y, et al. Comparison of antimicrobial activity of chitosan nanoparticles against bacteria and fungi. Coatings. 2021;11:769.

[133] Siritongsuk P, Thammawithan S, Srichaiyapol O, Nasompag S, Pongha S, Daduang S, et al. Synthesis and application of AgNPs-chitosan composite as a self-disinfecting coating in water-based polyurethane. Coatings. 2022;12:1832.

[134] Lee SJ, Nah H, Heo DN, Kim K-H, Seok JM, Heo M, et al. Induction of osteogenic differentiation in a rat calvarial bone defect model using an in situ forming graphene oxide incorporated glycol chitosan/oxidized hyaluronic acid injectable hydrogel. Carbon. 2020;168:264–77.

[135] Li P, Fu L, Liao Z, Peng Y, Ning C, Gao C, et al. Chitosan hydrogel/3D-printed poly(ε-caprolactone) hybrid scaffold containing synovial mesenchymal stem cells for cartilage regeneration based on tetrahedral framework nucleic acid recruitment. Biomaterials. 2021;278:121131. 34543785 10.1016/j.biomaterials.2021.121131

[136] Arockiasamy FS, Manoharan B, Santhi VM, Prakalathan K, Periasamy D, Dhandapani A, et al. Navigating the nano-world future: harnessing cellulose nanocrystals from green sources for sustainable innovation. Heliyon. 2025;11:e41188. 39811333 10.1016/j.heliyon.2024.e41188 PMC11730545

[137] Pelegrini BL, Ré F, de Oliveira MM, Fernandes T, de Oliveira JH, Oliveira Junior AG, et al. Cellulose nanocrystals as a sustainable raw material: cytotoxicity and applications on healthcare technology. Macromol Mater Eng. 2019;304:1900092.

[138] Tamo AK. Nanocellulose-based hydrogels as versatile materials with interesting functional properties for tissue engineering applications. J Mater Chem B. 2024;12:7692–759. 38805188 10.1039/d4tb00397g

[139] Tamo AK, Tran TA, Doench I, Jahangir S, Lall A, David L, et al. 3D printing of cellulase-laden cellulose nanofiber/chitosan hydrogel composites: towards tissue engineering functional biomaterials with enzyme-mediated biodegradation. Materials. 2022. 10.3390/ma15176039. 36079419 10.3390/ma15176039 PMC9456765

[140] Ao H, Jiang W, Nie Y, Zhou C, Zong J, Liu M, et al. Engineering quaternized chitosan in the 3D bacterial cellulose structure for antibacterial wound dressings. Polym Test. 2020;86:106490.

[141] Iravani S, Varma RS. Plants and plant-based polymers as scaffolds for tissue engineering. Green Chem. 2019;21:4839–67.

[142] Saracoglu P, Dokuz S, Ozbek T, Topuzogullari M, Ozmen MM. Starch nanogels as promising drug nanocarriers in the management of oral bacterial infections. J Drug Deliv Sci Technol. 2023;88:104973.

[143] Asl MA, Karbasi S, Beigi-Boroujeni S, Zamanlui Benisi S, Saeed M. Evaluation of the effects of starch on polyhydroxybutyrate electrospun scaffolds for bone tissue engineering applications. Int J Biol Macromol. 2021;191:500–13. 34555400 10.1016/j.ijbiomac.2021.09.078

[144] Ullah I, Chen Z, Xie Y, Khan SS, Singh S, Yu C, et al. Recent advances in biological activities of lignin and emerging biomedical applications: a short review. Int J Biol Macromol. 2022;208:819–32. 35364209 10.1016/j.ijbiomac.2022.03.182

[145] Ranakoti L, Gangil B, Bhandari P, Singh T, Sharma S, Singh J, et al. Promising role of polylactic acid as an ingenious biomaterial in scaffolds, drug delivery, tissue engineering, and medical implants: research developments, and prospective applications. Molecules. 2023;28:485. 36677545 10.3390/molecules28020485 PMC9861437

[146] Amini AR, Laurencin CT, Nukavarapu SP. Bone tissue engineering: recent advances and challenges. Crit Rev Biomed Eng. 2012;40:363–408. 23339648 10.1615/critrevbiomedeng.v40.i5.10 PMC3766369

[147] Klemeš JJ, Fan YV, Jiang P. Plastics: friends or foes? The circularity and plastic waste footprint. Energy Sources Part A Recover Util Environ Eff. 2021;43:1549–65.

[148] Atanasov AG, Waltenberger B, Pferschy-Wenzig E-M, Linder T, Wawrosch C, Uhrin P, et al. Discovery and resupply of pharmacologically active plant-derived natural products: a review. Biotechnol Adv. 2015;33:1582–614. 26281720 10.1016/j.biotechadv.2015.08.001 PMC4748402

[149] Roy H, Nayak BS, Nandi S. Chitosan anchored nanoparticles in current drug development utilizing computer-aided pharmacokinetic modeling: case studies for target specific cancer treatment and future prospective. Curr Pharm Des. 2020;26:1666–75. 32013823 10.2174/1381612826666200203121241

[150] Nuutila K, Laukkanen A, Lindford A, Juteau S, Nuopponen M, Vuola J, et al. Inhibition of skin wound contraction by nanofibrillar cellulose hydrogel. Plast Reconstr Surg. 2018;141:e357–66. 10.1097/PRS.0000000000004168 29135893

[151] Ashrafi G, Nasrollahzadeh M, Jaleh B, Sajjadi M, Ghafuri H. Biowaste- and nature-derived (nano)materials: biosynthesis, stability and environmental applications. Adv Colloid Interface Sci. 2022;301:102599. 35066374 10.1016/j.cis.2022.102599

[152] Di Domenico EG, Rimoldi SG, Cavallo I, D’Agosto G, Trento E, Cagnoni G, et al. Microbial biofilm correlates with an increased antibiotic tolerance and poor therapeutic outcome in infective endocarditis. BMC Microbiol. 2019;19:228. 31638894 10.1186/s12866-019-1596-2 PMC6802308

[153] Wang J, Chen X-Y, Zhao Y, Yang Y, Wang W, Wu C, et al. pH-switchable antimicrobial nanofiber networks of hydrogel eradicate biofilm and rescue stalled healing in chronic wounds. ACS Nano. 2019;13:11686–97. 31490650 10.1021/acsnano.9b05608

[154] Liang Y, Li M, Yang Y, Qiao L, Xu H, Guo B. Ph/Glucose dual responsive Metformin release hydrogel dressings with adhesion and self-healing via dual-dynamic bonding for athletic diabetic foot wound healing. ACS Nano. 2022;16:3194–207. 35099927 10.1021/acsnano.1c11040

[155] Li N, Liu W, Zheng X, Wang Q, Shen L, Hui J, et al. Antimicrobial hydrogel with multiple pH-responsiveness for infected burn wound healing. Nano Res. 2023;16:11139–48.

[156] Lozano Chamizo L, Luengo Morato Y, Ovejero Paredes K, Contreras Caceres R, Filice M, Marciello M. Ionotropic gelation-based synthesis of chitosan-metal hybrid nanoparticles showing combined antimicrobial and tissue regenerative activities. Polymers. 2021;13:3910. 34833209 10.3390/polym13223910 PMC8618652

[157] Islam R, Bilal H, Wang X, Zhang L. Tripeptides Ghk and GhkCu-modified silver nanoparticles for enhanced antibacterial and wound healing activities. Colloids and Surfaces B: Biointerfaces. 2024;236:113785. 38387323 10.1016/j.colsurfb.2024.113785

[158] Chen X, Li H, Qiao X, Jiang T, Fu X, He Y, et al. Agarose oligosaccharide- silver nanoparticle- antimicrobial peptide- composite for wound dressing. Carbohydr Polym. 2021;269:118258. 34294293 10.1016/j.carbpol.2021.118258

[159] Jia Q, Fu Z, Li Y, Kang Z, Wu Y, Ru Z, et al. Hydrogel loaded with peptide-containing nanocomplexes: symphonic cooperation of photothermal antimicrobial nanoparticles and prohealing peptides for the treatment of infected wounds. ACS Appl Mater Interfaces. 2024;16:13422–38. 38442213 10.1021/acsami.3c16061

[160] Qin P, Tang J, Sun D, Yang Y, Liu N, Li Y, et al. Zn2 + cross-linked alginate carrying hollow silica nanoparticles loaded with RL-QN15 peptides provides promising treatment for chronic skin wounds. ACS Appl Mater Interfaces. 2022;14:29491–505. 35731847 10.1021/acsami.2c03583

[161] Liang Y, Li Z, Huang Y, Yu R, Guo B. Dual-dynamic-bond cross-linked antibacterial adhesive hydrogel sealants with on-demand removability for post-wound-closure and infected wound healing. ACS Nano. 2021;15:7078–93. 33764740 10.1021/acsnano.1c00204

[162] Duan Y, Jiang F, Li Q, McDowell A, Li Y, Wang Y, et al. Multifunctional polysaccharide/metal/polyphenol double-crosslinked hydrogel for infected wound. Carbohydr Polym. 2024;332:121912. 38431415 10.1016/j.carbpol.2024.121912

[163] He J, Shi M, Liang Y, Guo B. Conductive adhesive self-healing nanocomposite hydrogel wound dressing for photothermal therapy of infected full-thickness skin wounds. Chem Eng J. 2020;394:124888.

[164] Stapleton LM, Steele AN, Wang H, Lopez Hernandez H, Yu AC, Paulsen MJ, et al. Use of a supramolecular polymeric hydrogel as an effective post-operative pericardial adhesion barrier. Nat Biomed Eng. 2019;3:611–20. 31391596 10.1038/s41551-019-0442-z PMC13371111

[165] Giano MC, Ibrahim Z, Medina SH, Sarhane KA, Christensen JM, Yamada Y, et al. Injectable bioadhesive hydrogels with innate antibacterial properties. Nat Commun. 2014;5:4095. 24958189 10.1038/ncomms5095 PMC4096704

[166] Zhao X, Wu H, Guo B, Dong R, Qiu Y, Ma PX. Antibacterial anti-oxidant electroactive injectable hydrogel as self-healing wound dressing with hemostasis and adhesiveness for cutaneous wound healing. Biomaterials. 2017;122:34–47. 28107663 10.1016/j.biomaterials.2017.01.011

[167] Deng Z, Wang H, Ma PX, Guo B. Self-healing conductive hydrogels: preparation, properties and applications. Nanoscale. 2020;12:1224–46. 31859313 10.1039/c9nr09283h

[168] Li Y, Fu R, Duan Z, Zhu C, Fan D. Artificial nonenzymatic antioxidant MXene nanosheet-anchored injectable hydrogel as a mild photothermal-controlled oxygen release platform for diabetic wound healing. ACS Nano. 2022;16:7486–502. 35533294 10.1021/acsnano.1c10575

[169] Sun M, Zhu C, Long J, Lu C, Pan X, Wu C. PLGA microsphere-based composite hydrogel for dual delivery of Ciprofloxacin and ginsenoside Rh2 to treat *Staphylococcus aureus*-induced skin infections. Drug Deliv. 2020;27:632–41. 32329376 10.1080/10717544.2020.1756985 PMC7241502

[170] Roy DC, Tomblyn S, Burmeister DM, Wrice NL, Becerra SC, Burnett LR, et al. Ciprofloxacin-Loaded keratin hydrogels prevent Pseudomonas aeruginosa infection and support healing in a Porcine Full-Thickness excisional wound. Adv Wound Care. 2014;4:457–68. 10.1089/wound.2014.0576 PMC4505758 26244102

[171] Blanco-Fernandez B, Lopez-Viota M, Concheiro A, Alvarez-Lorenzo C. Synergistic performance of cyclodextrin–agar hydrogels for ciprofloxacin delivery and antimicrobial effect. Carbohydr Polym. 2011;85:765–74.

[172] Papaioannou A, Liakopoulou A, Papoulis D, Gianni E, Gkolfi P, Zygouri E, et al. Effect of peptides on the synthesis, properties and wound healing capacity of silver nanoparticles. Pharmaceutics. 2023. 10.3390/pharmaceutics15102471. 37896231 10.3390/pharmaceutics15102471 PMC10609782

[173] Gao S, Deng J, Su Z, Liu M, Tang S, Hu T, et al. Turning polysaccharides into injectable and rapid self-healing antibacterial hydrogels for antibacterial treatment and bacterial-infected wound healing. Langmuir. 2024;40:9082–96. 38619979 10.1021/acs.langmuir.4c00451

[174] Tang X, Gu X, Wang Y, Chen X, Ling J, Yang Y. Stable antibacterial polysaccharide-based hydrogels as tissue adhesives for wound healing. RSC Adv. 2020;10:17280–7. 35521469 10.1039/d0ra02017f PMC9053413

[175] Zhou S, Zhang X, Ni W, He Y, Li M, Wang C, et al. An immune-regulating polysaccharide hybrid hydrogel with mild photothermal effect and anti-inflammatory for accelerating infected wound healing. Adv Healthc Mater. 2024;13:2400003. 10.1002/adhm.202400003 38711313

[176] Yang Q, Mo C, Cui P, Zhou S, Qiu L, Jiang P, et al. Application of f-FeNC@GOx cascade enzyme nanomaterials in the healing of infected wounds. Life Sci. 2023;329:121930. 37454755 10.1016/j.lfs.2023.121930

[177] Li Q, Dong M, Han Q, Zhang Y, Yang D, Wei D, et al. Enhancing diabetic wound healing with a pH-responsive nanozyme hydrogel featuring multi-enzyme-like activities and oxygen self-supply. J Controlled Release. 2024;365:905–18. 10.1016/j.jconrel.2023.12.015 38092256

[178] Geleto SA, Ariti AM, Gutema BT, Abda EM, Abiye AA, Abay SM, et al. Nanocellulose/Fe3O4/Ag nanozyme with robust peroxidase activity for enhanced antibacterial and wound healing applications. ACS Omega. 2023;8:48764–74. 38162792 10.1021/acsomega.3c05748 PMC10753546

[179] Yang Y, Wang J, Huang S, Li M, Chen J, Pei D, et al. Bacteria-responsive programmed self-activating antibacterial hydrogel to remodel regeneration microenvironment for infected wound healing. Natl Sci Rev. 2024;11:nwae044. 38440214 10.1093/nsr/nwae044 PMC10911815

[180] Negut I, Grumezescu V, Grumezescu AM. Treatment strategies for infected wounds. Molecules. 2018. 10.3390/molecules23092392. 30231567 10.3390/molecules23092392 PMC6225154

[181] Jiang L, Loo SCJ. Intelligent nanoparticle-based dressings for bacterial wound infections. ACS Appl Bio Mater. 2021;4:3849–62. 34056562 10.1021/acsabm.0c01168 PMC8155196

[182] Kudinov VA, Artyushev RI, Zurina IM, Lapshin RD, Snopova LB, Mukhina IV, et al. Antimicrobial and regenerative effects of placental multipotent mesenchymal stromal cell secretome-based chitosan gel on infected burns in rats. Pharmaceuticals. 2021. 10.3390/ph14121263. 34959663 10.3390/ph14121263 PMC8707738

[183] Arafa MG, El-Kased RF, Elmazar MM. Thermoresponsive gels containing gold nanoparticles as smart antibacterial and wound healing agents. Sci Rep. 2018;8:13674. 30209256 10.1038/s41598-018-31895-4 PMC6135834

[184] Shahriari-Khalaji M, Sattar M, Cao R, Zhu M. Angiogenesis, hemocompatibility and bactericidal effect of bioactive natural polymer-based bilayer adhesive skin substitute for infected burned wound healing. Bioact Mater. 2023;29:177–95. 37520303 10.1016/j.bioactmat.2023.07.008 PMC10384635

[185] Zhou K, Wang S, Xu L, Li H, Wang Y, Qiu Z, et al. Aiegen-based smart system for fungal-infected wound monitoring and on-demand photodynamic therapy. Matter. 2023;6:3449–62.

[186] Mirani B, Hadisi Z, Pagan E, Dabiri SMH, van Rijt A, Almutairi L, et al. Smart dual-sensor wound dressing for monitoring cutaneous wounds. Adv Healthc Mater. 2023;12:2203233. 36929644 10.1002/adhm.202203233 PMC11468884

[187] Ruggeri M, Vigani B, Boselli C, Icaro Cornaglia A, Colombo D, Sànchez-Espejo R, et al. Smart nano-in-microparticles to tackle bacterial infections in skin tissue engineering. Mater Today Bio. 2022;16:100418. 36157051 10.1016/j.mtbio.2022.100418 PMC9489812

[188] Ahmad N, Bukhari SNA, Hussain MA, Ejaz H, Munir MU, Amjad MW. Nanoparticles incorporated hydrogels for delivery of antimicrobial agents: developments and trends. RSC Adv. 2024;14:13535–64. 38665493 10.1039/d4ra00631c PMC11043667

[189] Kurian AG, Singh RK, Sagar V, Lee J-H, Kim H-W. Nanozyme-engineered hydrogels for anti-inflammation and skin regeneration. Nano-Micro Lett. 2024;16:110. 10.1007/s40820-024-01323-6 PMC10847086 38321242

[190] Ouyang J, Ji X, Zhang X, Feng C, Tang Z, Kong N, et al. In situ sprayed NIR-responsive, analgesic black phosphorus-based gel for diabetic ulcer treatment. Proc Natl Acad Sci U S A. 2020;117:28667–77. 33139557 10.1073/pnas.2016268117 PMC7682336

[191] Deng X, Wu Y, Tang Y, Ge Z, Wang D, Zheng C, et al. Microenvironment-responsive smart hydrogels with antibacterial activity and immune regulation for accelerating chronic wound healing. J Control Release. 2024;368:518–32. 38462042 10.1016/j.jconrel.2024.03.002

[192] Silva AC, dos Santos AGR, Pieretti JC, Rolim WR, Seabra AB, Ávila DS. Iron oxide/silver hybrid nanoparticles impair the cholinergic system and cause reprotoxicity in *Caenorhabditis elegans*. Food Chem Toxicol. 2023;179:113945. 37451599 10.1016/j.fct.2023.113945

[193] Kumar R, Bauri S, Sahu S, Chauhan S, Dholpuria S, Ruokolainen J, et al. Vivo toxicological analysis of MnFe2O4@poly(tBGE-alt-PA) composite as a hybrid nanomaterial for possible biomedical use. ACS Appl Bio Mater. 2023;6:1122–32. 36757355 10.1021/acsabm.2c00983 PMC10031559

[194] Privalova LI, Katsnelson BA, Loginova NV, Gurvich VB, Shur VY, Valamina IE, et al. Subchronic toxicity of copper oxide nanoparticles and its attenuation with the help of a combination of bioprotectors. Int J Mol Sci. 2014. 10.3390/ijms150712379. 25026171 10.3390/ijms150712379 PMC4139849

[195] Park S, Lee YK, Jung M, Kim KH, Chung N, Ahn E-K, et al. Cellular toxicity of various inhalable metal nanoparticles on human alveolar epithelial cells. Inhal Toxicol. 2007;19:59–65. 17886052 10.1080/08958370701493282

[196] Jarrar B, Almansour M, Jarrar Q, Al-Doaiss A, Lee SY, Boudemagh D. Toxicity assessment of copper oxide nanoparticles: in vivo study. Nanotechnology Rev. 2024. 10.1515/ntrev-2024-0122.

[197] Zhou Y, Sun P, Cao Y, Yang J, Wu Q, Peng J. Biocompatible copper formate-based nanoparticles with strong antibacterial properties for wound healing. J Nanobiotechnol. 2023;21:474. 10.1186/s12951-023-02247-w PMC10710715 38072979

[198] Yang K, Ganesan K, Gao F, Xie C, Chen J. Subacute toxicity of isoliquiritigenin-zein phosphatidylcholine nanoparticles on biochemical, hematological, and histopathological parameters in Sprague-Dawley rats. Explor Drug Sci. 2024;2:234–53.

[199] Ali Khan S, Ahmed N, Khan D, Bibi A, Rehman A. pH-responsive mupirocin-loaded hybrid nanoparticles in hydrogel and film forming spray against resistant bacterial wound infections. J Drug Deliv Sci Technol. 2024;101:106187.

[200] Aflakian F, Mirzavi F, Aiyelabegan HT, Soleimani A, Gholizadeh Navashenaq J, Karimi-Sani I, et al. Nanoparticles-based therapeutics for the management of bacterial infections: a special emphasis on FDA approved products and clinical trials. Eur J Pharm Sci. 2023;188:106515. 37402428 10.1016/j.ejps.2023.106515

[201] Wang H, Zhou S, Guo L, Wang Y, Feng L. Intelligent hybrid hydrogels for rapid in situ detection and photothermal therapy of bacterial infection. ACS Appl Mater Interfaces. 2020;12:39685–94. 32805886 10.1021/acsami.0c12355

[202] Liu D, Yang F, Xiong F, Gu N. The smart drug delivery system and its clinical potential. Theranostics. 2016;6:1306–23. 27375781 10.7150/thno.14858 PMC4924501

[203] Rasal RM, Janorkar AV, Hirt DE. Poly(lactic acid) modifications. Prog Polym Sci. 2010;35:338–56.

[204] Dhali SL, Parida D, Kumar B, Bala K. Recent trends in microbial and enzymatic plastic degradation: a solution for plastic pollution predicaments. Biotechnology for Sustainable Materials. 2024;1(1):11.

[205] Hakkarainen M, Albertsson A-C. Environmental degradation of polyethylene. In: Albertsson A-C, editor. Long term properties of polyolefins. Berlin, Heidelberg: Springer Berlin Heidelberg; 2004. pp. 177–200.

[206] Cabeza C, van Lier JB, van der Steen P. Effects of thermal and enzymatic pre-treatments on the solubilisation of extracellular polymeric substances (EPS) and subsequent anaerobic digestion of microalgae-bacterial biomass. Algal Res. 2023;72:103130.

[207] Ariza-Tarazona MC, Siligardi C, Carreón-López HA, Valdéz-Cerda JE, Pozzi P, Kaushik G, et al. Low environmental impact remediation of microplastics: visible-light photocatalytic degradation of PET microplastics using bio-inspired C,N-TiO2/SiO2 photocatalysts. Mar Pollut Bull. 2023;193:115206. 37392590 10.1016/j.marpolbul.2023.115206

[208] Chi Q, Xu T, He Y, Li Z, Tang X, Fan X, et al. Polystyrene nanoparticle exposure supports ROS-NLRP3 axis-dependent DNA-NET to promote liver inflammation. J Hazard Mater. 2022;439:129502. 35868089 10.1016/j.jhazmat.2022.129502

[209] Liang Y, Yang Y, Lu C, Cheng Y, Jiang X, Yang B, et al. Polystyrene nanoplastics exposure triggers spermatogenic cell senescence via the Sirt1/ROS axis. Ecotoxicol Environ Saf. 2024;279:116461. 38763051 10.1016/j.ecoenv.2024.116461

[210] Yang D, Zhu J, Zhou X, Pan D, Nan S, Yin R, et al. Polystyrene micro- and nano-particle coexposure injures fetal thalamus by inducing ROS-mediated cell apoptosis. Environ Int. 2022;166:107362. 35749991 10.1016/j.envint.2022.107362

[211] Zhang Y, Jia Z, Gao X, Zhao J, Zhang H. Polystyrene nanoparticles induced mammalian intestine damage caused by blockage of BNIP3/NIX-mediated mitophagy and gut microbiota alteration. Sci Total Environ. 2024;907:168064. 37884137 10.1016/j.scitotenv.2023.168064

[212] Ajonuma L, Bamiro S, Adeyinka R, Fapohunda D, Makanjuola S. P-100 Prolonged exposure to polyethylene glycol (peg) 6000 severely affects the reproductive system of male Wistar rats. Hum Reprod. 2024;39:deae108474.

[213] Bandow N, Aitken MD, Geburtig A, Kalbe U, Piechotta C, Schoknecht U, et al. Using environmental simulations to test the release of hazardous substances from polymer-based products. Are realism and pragmatism mutually exclusive objectives? Materials. 2020. 10.3390/ma13122709. 32549187 10.3390/ma13122709 PMC7345583

[214] Varg JE, Svanbäck R. Multi stress system: microplastics in freshwater and their effects on host microbiota. Sci Total Environ. 2023;856:159106. 36183774 10.1016/j.scitotenv.2022.159106

[215] Tao J, Deng P, Lin M, Chen C, Ma Q, Yang L, et al. Long-term exposure to polystyrene microplastics induces hepatotoxicity by altering lipid signatures in C57BL/6J mice. Chemosphere. 2024;347:140716. 37979802 10.1016/j.chemosphere.2023.140716

[216] Zhang X, Yin Z, Xiang S, Yan H, Tian H. Degradation of polymer materials in the environment and its impact on the health of experimental animals: a review. Polymers (Basel). 2024. 10.3390/polym16192807. 39408516 10.3390/polym16192807 PMC11478708

[217] Chang SH. Plastic waste as pyrolysis feedstock for plastic oil production: a review. Sci Total Environ. 2023;877:162719. 36933741 10.1016/j.scitotenv.2023.162719

[218] Su HY, Lai CS, Lee KH, Chiang YW, Chen CC, Hsu PC. Prenatal exposure to low-dose di-(2-ethylhexyl) phthalate (DEHP) induces potentially hepatic lipid accumulation and fibrotic changes in rat offspring. Ecotoxicol Environ Saf. 2024;269:115776. 38056127 10.1016/j.ecoenv.2023.115776

[219] Zhang Q, Zhang Y, Jing L, Zhao H. Microplastics induced inflammation in the spleen of developmental Japanese quail (*Coturnix japonica*) via ROS-mediated p38 MAPK and TNF signaling pathway activation1. Environ Pollut. 2024;341:122891. 37951530 10.1016/j.envpol.2023.122891

[220] Bishop CR, Yan K, Nguyen W, Rawle DJ, Tang B, Larcher T, et al. Microplastics dysregulate innate immunity in the SARS-CoV-2 infected lung. Front Immunol. 2024;15. 10.3389/fimmu.2024.1382655. 38803494 10.3389/fimmu.2024.1382655 PMC11128561

[221] Xiao M, Zhang Y, Zhang X, Zhang G, Jin C, Yang J, et al. Bisphenol A and Di(2-ethylhexyl) phthalate promote pulmonary carcinoma in female rats via Estrogen receptor beta: in vivo and in silico analysis. Ecotoxicol Environ Saf. 2023;250:114496. 36608567 10.1016/j.ecoenv.2022.114496

[222] Ma Y, Xu D, Wan Z, Wei Z, Chen Z, Wang Y, et al. Exposure to different surface-modified polystyrene nanoparticles caused anxiety, depression, and social deficit in mice via damaging mitochondria in neurons. Sci Total Environ. 2024;919:170739. 38340854 10.1016/j.scitotenv.2024.170739

[223] Elje E, Mariussen E, Moriones OH, Bastús NG, Puntes V, Kohl Y, et al. Hepato(geno)toxicity assessment of nanoparticles in a HepG2 liver spheroid model. Nanomaterials. 2020. 10.3390/nano10030545. 32197356 10.3390/nano10030545 PMC7153628

[224] Guo J, Liu N, Xie Q, Zhu L, Ge F. Polystyrene microplastics facilitate the biotoxicity and biomagnification of ZnO nanoparticles in the food chain from algae to daphnia. Environ Pollut. 2023;324:121181. 36736564 10.1016/j.envpol.2023.121181

[225] Wu Z, Nie R, Wang Y, Wang Q, Li X, Liu Y. Precise antibacterial therapeutics based on stimuli-responsive nanomaterials. Front Bioeng Biotechnol. 2023. 10.3389/fbioe.2023.1289323. 37920242 10.3389/fbioe.2023.1289323 PMC10619694

[226] Perumalsamy H, Xiao X, Kim H-Y, Yoon T-H. Scrna-seq analysis discovered suppression of immunomodulatory dependent inflammatory response in PMBCs exposed to silver nanoparticles. J Nanobiotechnol. 2024;22:118. 10.1186/s12951-024-02364-0 PMC10946150 38494495

[227] Bae J, Ha M, Perumalsamy H, Lee Y, Song J, Yoon T-H. Mass cytometry exploration of immunomodulatory responses of human immune cells exposed to silver nanoparticles. Pharmaceutics. 2022. 10.3390/pharmaceutics14030630. 35336005 10.3390/pharmaceutics14030630 PMC8954471

[228] Nosrati H, Nosrati M. Artificial intelligence in regenerative medicine: applications and implications. Biomimetics. 2023;8:442. 37754193 10.3390/biomimetics8050442 PMC10526210

[229] Adir O, Poley M, Chen G, Froim S, Krinsky N, Shklover J, et al. Integrating artificial intelligence and nanotechnology for precision cancer medicine. Adv Mater. 2020;32:e1901989. 31286573 10.1002/adma.201901989 PMC7124889

[230] Lonsdale DO, Lipman J. Global personalization of antibiotic therapy in critically ill patients. Expert Rev Precis Med Drug Dev. 2021;6:87–93.

[231] Haddad-Boubaker S, Mbarek H, Yassine HM. Editorial: personalized medicine and infectious disease management. Front Med. 2023;10. 10.3389/fmed.2023.1191147. 10.3389/fmed.2023.1191147 PMC10216473 37250651

[232] Ahmad F, Mahmood A, Muhmood T. Machine learning-integrated omics for the risk and safety assessment of nanomaterials. Biomater Sci. 2021;9:1598–608. 33443512 10.1039/d0bm01672a

[233] Vicente AM, Ballensiefen W, Jönsson J-I. How personalised medicine will transform healthcare by 2030: the Icpermed vision. J Transl Med. 2020;18:180. 32345312 10.1186/s12967-020-02316-w PMC7189458

[234] Futane A, Jadhav P, Mustafa AH, Srinivasan A, Narayanamurthy V. Aptamer-functionalized MOFs and AI-driven strategies for early cancer diagnosis and therapeutics. Biotechnol Lett. 2024;46:1–17. 38155321 10.1007/s10529-023-03454-z

[235] Siddique MM, Seraj MMB, Adnan MN, Galib SM. Artificial intelligence for infectious disease detection: prospects and challenges. In: Chowdhury MEH, Kiranyaz S, editors. Surveillance, prevention, and control of infectious diseases: an AI perspective. Cham: Springer Nature Switzerland; 2024. pp. 1–22.

[236] Peng J, Jury EC, Donnes P, Ciurtin C. Machine learning techniques for personalised medicine approaches in immune-mediated chronic inflammatory diseases: applications and challenges. Front Pharmacol. 2021;12:720694. 34658859 10.3389/fphar.2021.720694 PMC8514674

[237] Gadzhimagomedova ZM, Pashkov DM, Kirsanova DY, Soldatov SA, Butakova MA, Chernov AV, et al. Artificial intelligence for nanostructured materials. Nanobiotechnology Reports. 2022;17:1–9.

[238] Liu L, Bi M, Wang Y, Liu J, Jiang X, Xu Z, et al. Artificial intelligence-powered microfluidics for nanomedicine and materials synthesis. Nanoscale. 2021;13:19352–66. 34812823 10.1039/d1nr06195j

[239] Kopac T. Leveraging artificial intelligence and machine learning for characterizing protein corona, nanobiological interactions, and advancing drug discovery. Bioengineering. 2025. 10.3390/bioengineering12030312. 40150776 10.3390/bioengineering12030312 PMC11939375

[240] Rafiei M, Shojaei A, Chau Y. Machine learning-assisted design of immunomodulatory lipid nanoparticles for delivery of mRNA to repolarize hyperactivated microglia. Drug Deliv. 2025;32:2465909. 40028722 10.1080/10717544.2025.2465909 PMC11878168

[241] Skepu A, Phakathi B, Makgoka M, Mbita Z, Damane BP, Demetriou D, et al. AI and nanomedicine in realizing the goal of precision medicine: tailoring the best treatment for personalized cancer treatment. In: Dlamini Z, editor. Artificial intelligence and precision oncology: bridging cancer research and clinical decision support. Cham: Springer Nature Switzerland; 2023. pp. 181–94.

[242] Guo K, Yang Z, Yu CH, Buehler MJ. Artificial intelligence and machine learning in design of mechanical materials. Mater Horiz. 2021;8:1153–72. 34821909 10.1039/d0mh01451f

[243] Porter B, Grippa F. A platform for AI-enabled real-time feedback to promote digital collaboration. Sustainability. 2020;12:10243.

[244] Rawson TM, Peiffer-Smadja N, Holmes A. Artificial intelligence in infectious diseases. In: Lidströmer N, Ashrafian H, editors. Artificial intelligence in medicine. Cham: Springer International Publishing; 2020. pp. 1–14.

[245] de la Lastra JMP, Wardell SJT, Pal T, de la Fuente-Nunez C, Pletzer D. From data to decisions: leveraging artificial intelligence and machine learning in combating antimicrobial resistance - a comprehensive review. J Med Syst. 2024;48:71. 39088151 10.1007/s10916-024-02089-5 PMC11294375

[246] Hrvat F, Aleta A, Džuho A, Hasanić O, Spahić Bećirović L. Artificial intelligence in nanotechnology: recent trends, challenges and future perspectives. In: Badnjevic A, Gurbeta Pokvić L, editors. CMBEBIH 2021. Cham: Springer International Publishing; 2021. pp. 690–702.

[247] Nandipati M, Fatoki O, Desai S. Bridging nanomanufacturing and artificial intelligence-a comprehensive review. Materials. 2024. 10.3390/ma17071621. 38612135 10.3390/ma17071621 PMC11012965

[248] Zhu X, Li Y, Gu N. Application of artificial intelligence in the exploration and optimization of biomedical nanomaterials. Nano Biomed Eng. 2023;15:342–53.

[249] Jessop ZM, Al-Sabah A, Francis WR, Whitaker IS. Transforming healthcare through regenerative medicine. BMC Med. 2016;14:115. 27510095 10.1186/s12916-016-0669-4 PMC4980802

[250] Wong AC, Levy M. New approaches to microbiome-based therapies. mSystems. 2019. 10.1128/mSystems.00122-19. 31164406 10.1128/mSystems.00122-19 PMC6584878

[251] Wang Y, Jang YY. From cells to organs: the present and future of regenerative medicine. Adv Exp Med Biol. 2022;1376:135–49. 34327664 10.1007/5584_2021_657 PMC9301257

[252] Bhutkar MA, Sonawane RO. Translating nanomaterials from laboratory to clinic: barriers ahead. In: Pardeshi CV, editor. Nanomaterial-Based drug delivery systems: therapeutic and theranostic applications. Cham: Springer International Publishing; 2023. pp. 381–405.

[253] Williams KL, Enslow R, Suresh S, Beaton C, Hodge M, Brooks AE. Using the microbiome as a regenerative medicine strategy for autoimmune diseases. Biomedicines. 2023;11:1582. 37371676 10.3390/biomedicines11061582 PMC10295452

